# Möbius-strip-like columnar functional connections are revealed in somato-sensory receptive field centroids

**DOI:** 10.3389/fnana.2014.00119

**Published:** 2014-10-31

**Authors:** James Joseph Wright, Paul David Bourke, Oleg Vyachesslavovich Favorov

**Affiliations:** ^1^Department of Psychological Medicine, Faculty of Medicine, The University of AucklandAuckland, New Zealand; ^2^iVEC@UWA, University of Western AustraliaPerth, WA, Australia; ^3^Department of Biomedical Engineering, The University of North Carolina at Chapel HillChapel Hill, NC, USA

**Keywords:** cortical column, cortical development, synaptic organization, cortical response properties, neuromicrocircuitry, S1 segregates

## Abstract

Receptive fields of neurons in the forelimb region of areas 3b and 1 of primary somatosensory cortex, in cats and monkeys, were mapped using extracellular recordings obtained sequentially from nearly radial penetrations. Locations of the field centroids indicated the presence of a functional system in which cortical homotypic representations of the limb surfaces are entwined in three-dimensional Möbius-strip-like patterns of synaptic connections. Boundaries of somatosensory receptive field in nested groups irregularly overlie the centroid order, and are interpreted as arising from the superposition of learned connections upon the embryonic order. Since the theory of embryonic synaptic self-organization used to model these results was devised and earlier used to explain findings in primary visual cortex, the present findings suggest the theory may be of general application throughout cortex and may reveal a modular functional synaptic system, which, only in some parts of the cortex, and in some species, is manifest as anatomical ordering into columns.

## Introduction

It is widely acknowledged that Mountcastle's (Mountcastle et al., 1955; Mountcastle, 1997) concept of the cortical column was critical to subsequent development in the theory of the cerebral cortex (Hubel, 1981; DeFelipe et al., 2012). However, the concept has proved problematic (Purves et al., 1992; Horton and Adams, 2005). The terminology describing supposed variants and orderings into columns has become confused (Rakic, 2008; Da Costa and Martin, 2010). DeFelipe et al. (2012) have concluded that a comprehensive framework of the function and structure of columns remains elusive, the classical criteria (shared response properties, shared input and common output) may need to be modified, and area-specific and species-specific variations more adequately defined, to achieve fundamental understanding of what columns are, and how they are used in cortical processes.

Opinions diverge on whether or not structural modularity is of functional importance at all. The utility of modularity, both for the actual operation of cognitive processes, and for our task of analysis of brain function, has been emphasized by Szentagothai (1983). A contrary opinion was presented explicitly by Purves et al. (1992) when they argued that “[columnar] patterns arise not because the functional organization of the brain demands them, but as an incidental consequence of the rules of synapse formation.” The latter interpretation, forced to its limit, suggests that no functional modularity exists in the cortex, and therefore the task of its analysis is greatly multiplied. However, there might exist modularity in the patterns of cortical synaptic connections, that become manifest as cellular anatomical order only under some circumstances. That is the position that will be argued here.

In this paper we apply a recent model for the organization of columnar structure in primary visual cortex (V1) to primary somatosensory cortex (S1), in areas anatomically not obviously columnar, to attempt to solve some of the dilemmas posed above. We will place emphasis on horizontal connectivity within cortex (Boucsein et al., 2011) and on organized, modular, patterns of synaptic connections, rather than on spatial clustering of cell bodies into defined columns. To avoid the many terminological difficulties, we will simply define “macrocolumns” as variably resolved clusters of neurons sharing relatively dense short-range lateral connectivity, and distinguish separately the long-range horizontal patch connections, which also have clustered cell bodies, but have patchy areas of synaptic termination, both patch-reciprocal and also terminating on the peripheries of macrocolumns. Reasonably clear macrocolumns in this sense are apparent only in V1 and in some other locales such as the barrel-based cortical columns representing vibrissae in S1 (Woolsey and Van der Loos, 1970)—sensory systems in which the organization of inputs is clear-cut—although even this degree of orderliness is only apparent in some species, and not in all situations with clear input demarcation (Purves et al., 1992).

Further adding to the difficulties of analysis, attempts to understand the relation between columnar organization and the response properties of single neurons have also struck problems (Blakemore and Cooper, 1970; Blakemore and Van Sluyters, 1975; Swindale, 1996). In V1, visual line orientation has long been considered a primary property (Hubel and Wiesel, 1959), but Fitzpatrick et al. (Basole et al., 2003) have shown that orientation preference (OP) is a function not only of visual line orientation, but also of the angle of the line to the direction of motion, the speed of motion, and the length of the line. So long as stimulus properties are clearly defined, individual neurons show clearly tuned specific responses, whether or not response maps can be clearly resolved, as can be shown when OP tuning is contrasted in macaque and rat (Girman et al., 1999). In V1, where response maps are relatively well resolved and continuous, Issa et al. (2008) have shown that the combined cell responses to orientation, spatial frequency, temporal frequency, and the bandwidths of each, define the response maps. In S1, although macrocolumns are not clearly defined anatomically outside the cortical barrels, organization of responses into combinations of stimulus features is also indicated by the way that receptive field (RF) boundaries form “segregates,” as next discussed.

Via dorsal-column-medial-lemniscus-cortical pathways, a homotypic representation of the skin surface is projected to the horizontal plane of the S1cortex (e.g., Marshall et al., 1941) and in Mountcastle's conception of the columnar organization the homotypic map has depth from the pial surface to the white matter, with different afferent submodalities in a mosaic ordering. Exploring this concept in S1 forelimb representations, Favorov et al. (1987); Favorov and Whitsel (1988a,b); Favorov and Diamond (1990) advanced extracellular recording microelectrodes completely through the cortex at near-radial angles deviating on average about 22.5 degrees from the vertical. As expected, the more closely the path of descent approached the vertical (radial) track, the more similar became the RFs of the sequentially recorded single units. With the slight oblique drift of the electrode path they commonly encountered sequences of RFs that approximated an homotypic map, but there were also sudden discontinuities, where the RF of the next neuron found in the electrode's progress leapt to a relatively distant position on the skin surface—sometimes returning back closer to the prior localities as the electrode was still further advanced.

Using the skin locations of the maximum response (the “minRF”) to tactile stimulation of local groups of neurons in a close vicinity to the tip of the recording electrode—rather than the full-extent RFs (maxRFs) of single neurons, Favorov et al. (1987), Favorov and Diamond (1990) inferred the existence in S1 of functional macrocolumnar organization in a form of discrete, 300–600 μm wide columns, “segregates.” In such a mosaic arrangement, a somatotopic map of minRFs is represented in a discontinuous fashion, with essentially unchanging minRFs within the 300–600 μm wide cortical zones and abrupt jumps of minRFs to new skin locations when the electrode crosses a sharp boundary separating any two such zones.

Favorov et al. (1987) also found that when maxRFs sequentially recorded along a near-radial penetration were superimposed, they often shared a small area of common overlap. We will refer to these maxRF groups with common center, forming nests of RFs indicating neurons with spatial correlation of their inputs, as “nested RFs,” “RF nests,” or simply “nests.” Such nests were related to segregates defined on the basis of minRF mapping: all the neurons sampled within the same segregate shared a common skin locus in their maxRFs and thus belonged to a single nest, whereas only about half of neurons sampled in a near-radial penetration across a segregate boundary shared a common skin point. Thus, it appears that RF nests are centered on segregates but are larger than a single segregate.

We intend to show that the breaks in continuity of minRF positions found in S1 by Favorov et al. are explicable by a model of embryogenesis and synaptic self-organization independently developed to account for V1 columnar organization, and the nested RFs are an overlay of this primary order.

## The Möbius model of cortical development and its application in V1

During embryogenesis cells becoming cortical neurons divide and migrate to their mature positions while undergoing apotosis (Rakic, 1988, 2009). Wright and Bourke (2013) devised a theory for embryonic selection of neurons and their strongest synaptic connections that leads to a modular description of cortical organization—one in which the modular organization is that of the pattern of synaptic connections, but in which cellular order need be only variably apparent. The basic assumptions of the proposal rested on two experimental findings—firstly, *in vitro*, embryonic neurons fire synchronously and self-organize into “small worlds” (Downes et al., 2012) and secondly, synchronous firing of neurons prevents their apoptosis (Heck et al., 2008). We assumed synchrony and cell survival are directly causally linked—that by some mechanism, synchrony of pre-synaptic activity or of action potentials promotes the uptake of a critical resource. Calcium is a likely candidate as that resource (Montague, 1996) but many different metabolites might be critical at different stages of neurogenesis. But overall, as a consequence of the balance between uptake of, and demand for, the critical resources, the emergent cell network would be that selection of cell types, and their arrangement, maximizing the amplitude of synchrony while minimizing the metabolic demand per cell. Consequent steps in our argument were as follows:

(1) Synchrony, measured as either amplitude of local field potentials, or as variance of pulse rates of excitatory cells, we considered generated as described in our simulations of electrocortical activity (Wright, 2009, 2010). These simulations show gamma oscillation to arise from an oscillating equilibrium of excitatory/inhibitory interactions, and synchrony of the excitatory cells from summation of in-phase pre-synaptic signals vs. cancelation of out-of-phase pre-synaptic signals.
(2) Uptake of the resource from the extracellular environment we take to be proportional to the magnitude of presynaptic synchrony, while consumption of the resource is proportional to neuron size/length. Competition thus leads to selection of an ensemble of cells with the maximum number of pre-synapses and the shortest possible set of connections permitting interaction of all cells in the ensemble—that is, the ensemble must have the topology of a “small world” (Watts and Strogatz, 1998).
(3) In the dilute network of neuronal connections, metric distance of soma separation is analogous to “degree of separation” in the topological sense, whereas range of fiber connections, and thus the number of cells reached by connections from a given cell, is analogous to degree distribution. Therefore, on average, the probability density of synaptic connectivity between excitatory cells should decline with distance of their somas as a power function, as required in “ultra-small-worlds” (Cohen and Havlin, 2003).
(4) A power function is the sum of exponential functions, and pre-synaptic densities of cortical neurons decline roughly exponentially (Braitenberg and Schüz, 1991). Therefore, ultra-small-world connectivity can be approximated by selection of subpopulations of neurons with suitable axonal ranges. In reality a considerable variety of excitatory cell types is encountered. For simplicity we treat these as only two ideal populations—a local type, with dense short-range horizontal connectivity, roughly corresponding to spiny stellate and pyramidal neurons with short axonal trees, and a type with long intracortical axons—the superficial patch cells. Cortico-cortical connections are excluded from present consideration, but can be accommodated within the same theory. With only two subpopulations, approximation of a power function takes the form
  (1) $$\rho ({\left|q-r\right|}^{-A})\approx N_{\alpha }\lambda_{\alpha }e^{-\lambda_{\alpha }\left|q-r\right|}+N_{\beta }\lambda_{\beta }e^{-\lambda_{\beta }\left|q-r\right|}$$

where A is an exponent between 2 and 3 (Cohen and Havlin, 2003),

ρ is the optimal pre-synaptic density/distance relation, **q,r** are positions of excitatory cortical neurons, *N*_α_ and *N*_β_ are numbers of patch cells and local neurons respectively, and λ_α_ and λ_β_ are the corresponding inverse length constants of their axonal trees. For given λ_α_ and λ_β_, selection of cells in a specific ratio *N*_α_ / *N*_β_ must occur, to best approximate a power function.

(5) Any two populations of neurons conforming to Equation (1) sustain a magnitude of synchronous oscillation, *J*,
  (2) $$J\propto \int_{q}\int_{r}\left(N_{\alpha }\lambda_{\alpha }e^{-\lambda_{\alpha }\left|q-r\right|}+N_{\beta }\lambda_{\beta }e^{-\lambda_{\beta }\left|q-r\right|}\right)dqdr$$

Maximization of *J* requires cells occur in clusters of each type. Two limiting cases arise: At the limit where one cell type has markedly longer axons than the other, λ_α_ < < λ_β_ Equation (1) requires *N*_β_ > > *N*_α_, and therefore maximization of *J* in Equation (2) requires cells with relatively short axons be closely situated, causing clustering into a macrocolumn. Consequently the cells with long-range axons must form connections at longer range, enforcing a “patchy” connection system. In the limiting case arrangement into a fully demarcated hexagonal periodic system must emerges, where

(3) $$\frac{local\;cells}{local\;cells+patch\;cells}=\frac{N_{\beta }}{N_{\alpha }+N_{\beta }}\geq \frac{\pi }{2\sqrt{3}}$$

The relation in Equation (3) is that of the most efficient packing, and follows from the ratio of area of a circle to a hexagon.

**Figure 1:**
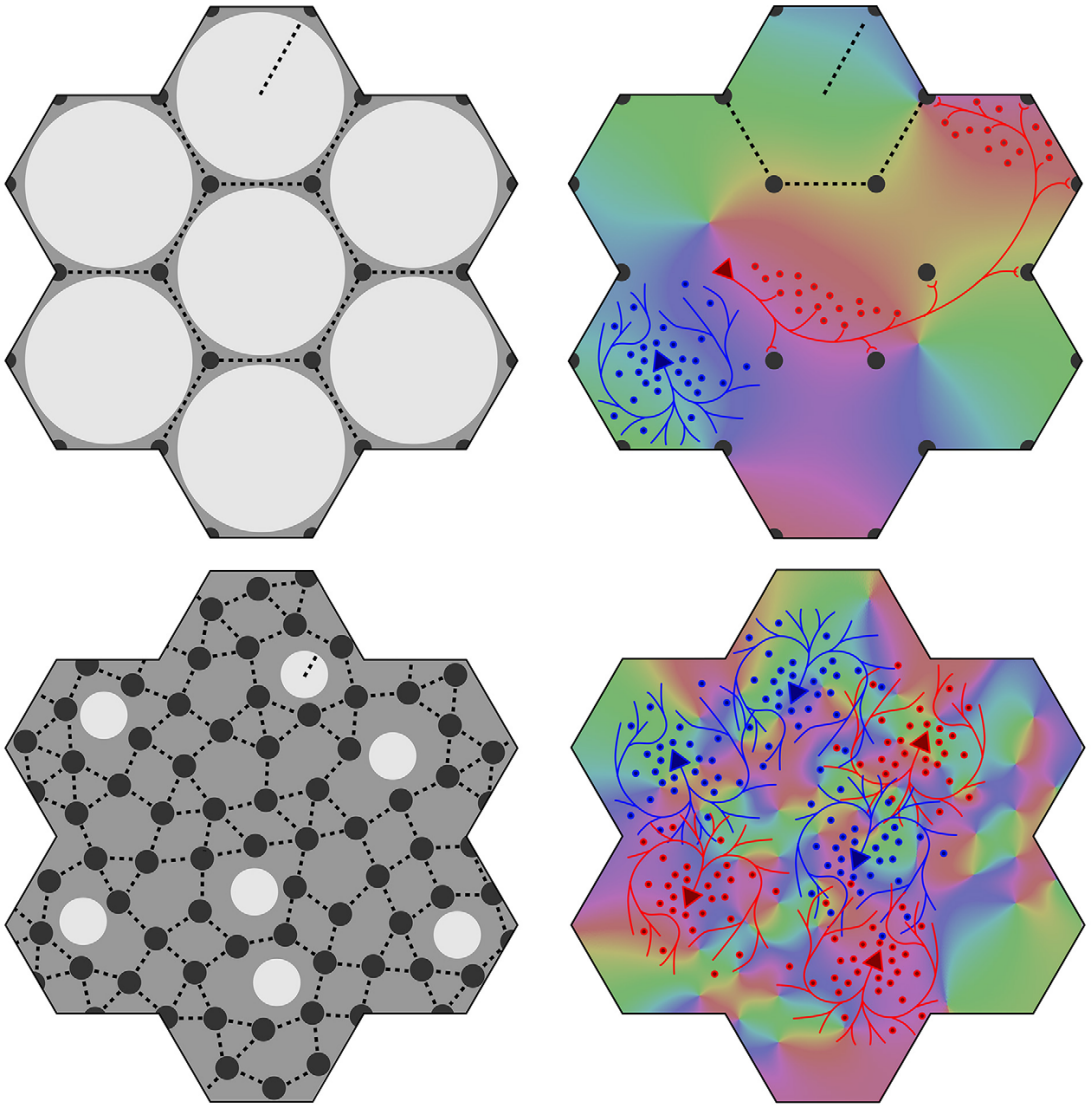
**Variation of the structure of macrocolumns at limiting extremes of the axonal lengths and cell numbers**. **Top left**: With large long/short axon length ratio, clearly resolved hexagonal organization emerges, with macrocolumns (white) surrounded by groups of patch cells (black dots). **Top right**: long (red line) patch connections link “like to like” OP (shown as background color wheel) in contrast to highly clustered short intracortical axons (blue lines). **Bottom left** and **right**: near-complete loss of resolution when long/short axon ratio approaches 1.

At an opposite limit, where λ_α_ ≈ λ_β_ Equation (1) requires *N*_β_ ≈ *N*_α_, and there is effectively only one cell type. Cell bodies can be intermingled, although Equations (1) and (2) are still met, and the absence of a clearly columnar arrangement does not imply a loss of the small world organization. The left hand images in Figure 1 show these limiting cases, suggesting how variations of macrocolumnar orderliness might arise as intermediates between the limiting cases, in different species and cortical areas.

**Figure 2:**
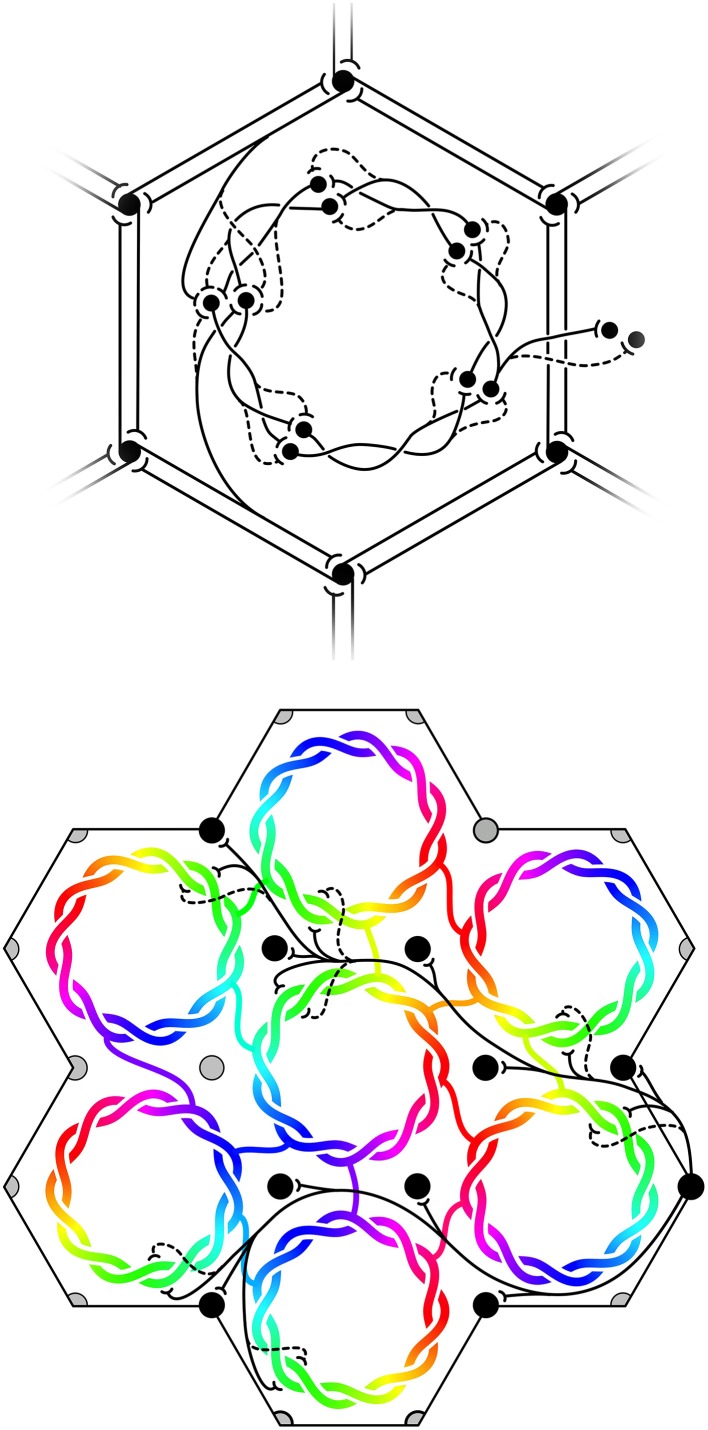
**Maximization of synchrony with local synaptic competition leads to Möbius ordering, within macrocolumns**. **Top**: Disposition of strong (solid) and weak (dashed) synapses in the developing neocortex. **Bottom**: “Like to like” patchy connections map the same part of the surrounding cortical field onto homologous cell positions on the Möbius configuration. Adjacent macrocolumns tend to mirror organization of OP.

(6) Maximization of *J* also requires that the patch connections project a 1:1 map of activity in the surrounding cortex onto each macrocolumn. Competition between closely situated presynaptic terminals arising from the same cell but terminating on different cells, would force strong synaptic links to some neighbors and weak links to others. The requirement to form a 1:1 map can then be met only if the strong local connections within the macrocolumn form a closed network analogous to a Möbius strip, in the sense that a series of strong connections tracing a continuous loop circling the center of the macrocolumn would need to circle the center twice before the loop was closed, as does a line traced on the familiar Möbius strip of a paper strip connected at its ends with a half-twist. The long-range patchy connections would then project the surrounding cortex onto this system as a Euclidean plane projected onto a Möbius strip. The right hand images in Figure 1 indicate how, in V1, this would force classical OP from 0 to 180° to be arranged from 0 to 360° about a singularity, and Figure 2 (top) shows how the strong and weak synaptic connections must be organized.
(7) Interactions between local maps must also maximize *J*. Therefore, patch connections must link “like to like” OP while forming multiple 1:1 maps, and adjacent macrocolumnar “maps” must be arranged so “like” map positions on adjacent macrocolumns are as closely situated as possible within the hexagonal frame, thus approximating a mirroring with continuity among adjacent maps. Figure 2 (bottom) shows these effects.

Applied to V1 before eye-opening, this model is able to explain:

- The emergence of macrocolumns (in some species) in V1 and OP response maps, their singularities, and continuity at column margins (e.g., Bosking et al., 1997).
- V1 approximation to hexagonal rotational periodicity (Muir et al., 2011; Paik and Ringach, 2011).
- Interspecies and inter-areal variation in superficial patch organization (Horton and Adams, 2005; Muir et al., 2011) and relative species invariance of intersingularity distances (Kaschube et al., 2010; Keil et al., 2012).
- Emergence (in some circumstances) of OD columns (Obermayer and Blasdel, 1993).
- Emergence of superficial patch connections, “like to like” (Gilbert and Wiesel, 1989; Muir et al., 2011) with patch-sparing of macrocolumn centers (Sharma et al., 1995; Muir and Douglas, 2011).
- Variation of apparent OP with stimulus orientation, angle of movement relative to orientation, object speed, and object length (Basole et al., 2003).

And after eye-opening, consequent to visual stimuli and Hebbian learning:

- Later absence of cortical responses to stimuli of which the subject is deprived (Blakemore and Cooper, 1970).
- The consolidation of learned cortical responses, producing selective tuning of cells stimulus orientation, spatial frequency, and temporal frequency (Issa et al., 2008).
- Complex stimulus responses in higher visual areas.

The theory implies both the retention of pre-natal Möbius ordering into post-natal life (evidenced by reproduction of the results of Basole et al.) and the overwriting of the earlier Möbius order by later learning, (accounting for the bandwidth tuning to classes of features shown in the work of Issa et al.).

It is not proposed that variation of genetically prescribed cell types is the only source of variation of columnar order. Spatial and temporal resolution of signals in the input pathways, degree of approximation to “brown” cross-correlation in inputs, amount of convergence and divergence of axo-synaptic connections, and Turing pattern formation can all be expected to contribute to the apparent order, and might in principle be included in more developed forms of this model.

We turn now to the application of the model to S1, in areas where no clear anatomical macrocolumnar order has been shown.

## Application to S1

### Mathematical considerations

Viewed from above the V1 cortical surface, the Möbius-like arrangement of OP resembles a 2:1 map formed by squaring a complex vector, creating a local representation of global positions,

(4) $$\pm P\to \frac{p^{2}}{\left|P\right|}$$

where *P* is a position in the visual field (and on the surface of V1), *p* is its mapped representation in the macrocolumn, the global and local maps have a common origin (*P*_0_ and *p*_0_) at the OP singularity, and are equivalently scaled.

However, this apparent 2:1 map conceals a 1:1 map in three dimensions, and application to sequential extracellular recordings on a trajectory passing from surface to depth in S1 requires consideration of three dimensions.

Applied in 3D, Equation (1) and maximization of *J* in Equation (2) requires local neurons with strong short-range synaptic connections to be packed closely together, forcing separation of the somata into two groups, one superficial and one deep, which we term “skeins”—the analog of each side of the paper in a paper Möbius strip. Onto these skeins of local cells projections conveyed by patch connections wind clockwise at one cortical level, and anti-clockwise at the other, with the projections circling a central zone, analogous to an OP singularity in V1.

**Figure 3:**
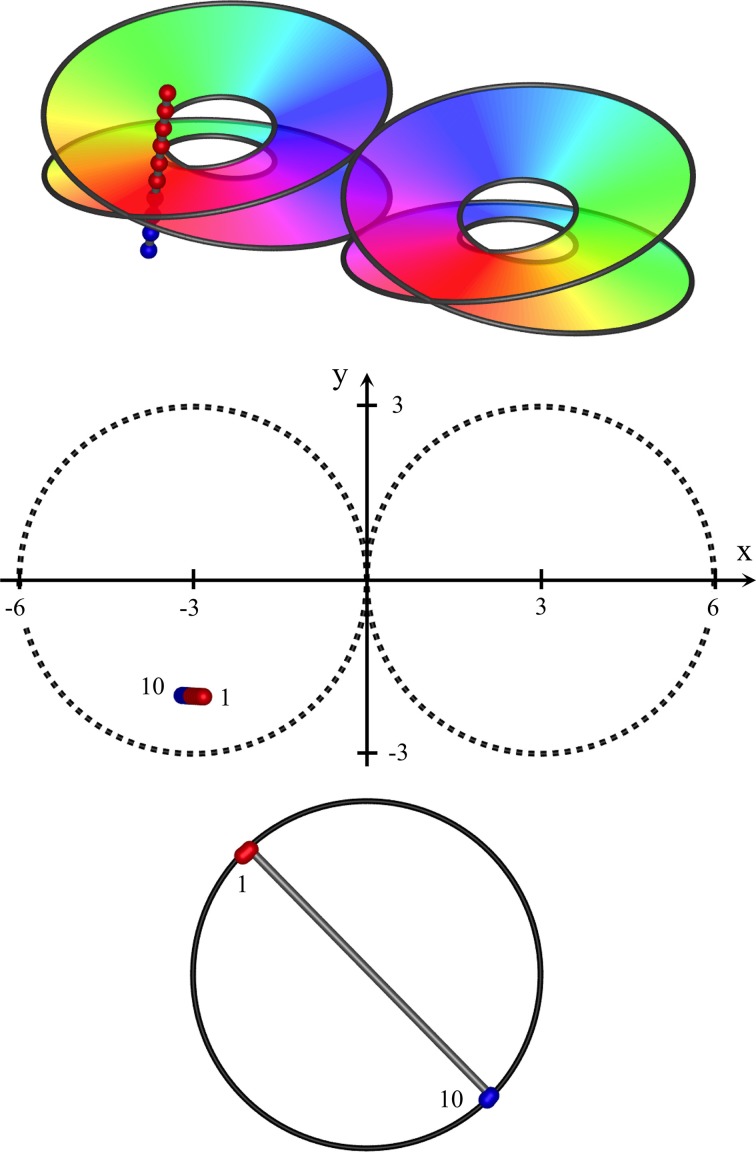
Near-vertical passage of a recording electrode through S1, from (*x, y, z*) = (−2.8, −2, 2) to (−3.2, −2, −1.2), *K* = 0.4. **Top**: Interfaces demarcating synaptic skeins in two adjacent macrocolumns, viewed from obliquely above cortical surface. Each of the left and right pairs of disks represent the interfaces between an upper skein and a lower skein of local cells, receiving projections via patchy connections from surrounding cortex, with a mixed area between upper and lower disks. The projections wind clockwise in one skein, and anticlockwise in the other. Colors of the spectrum on the disks indicate the direction of winding, reversed in the pair of macrocolumns. The track of the recording electrode is marked with red and blue dots to distinguish neurons in clockwise and anticlockwise skeins. **Middle**: positions of recorded neurons in plan view. First and last neurons discovered by the advancing recording electrode numbered accordingly. **Bottom**: RFs centroids, strongly clustered into two groups, associated with clockwise and anticlockwise skeins respectively.

Since complete skein separation could only be approached as ideal, at an intermediate level mixing and intertwining of the skeins is anticipated. The skeins are necessarily continuous at some angle directed from the central singularity, so mixing at the skeins would be lesser at the continuity. (See Figure 3, Top panels).

**Figure 4:**
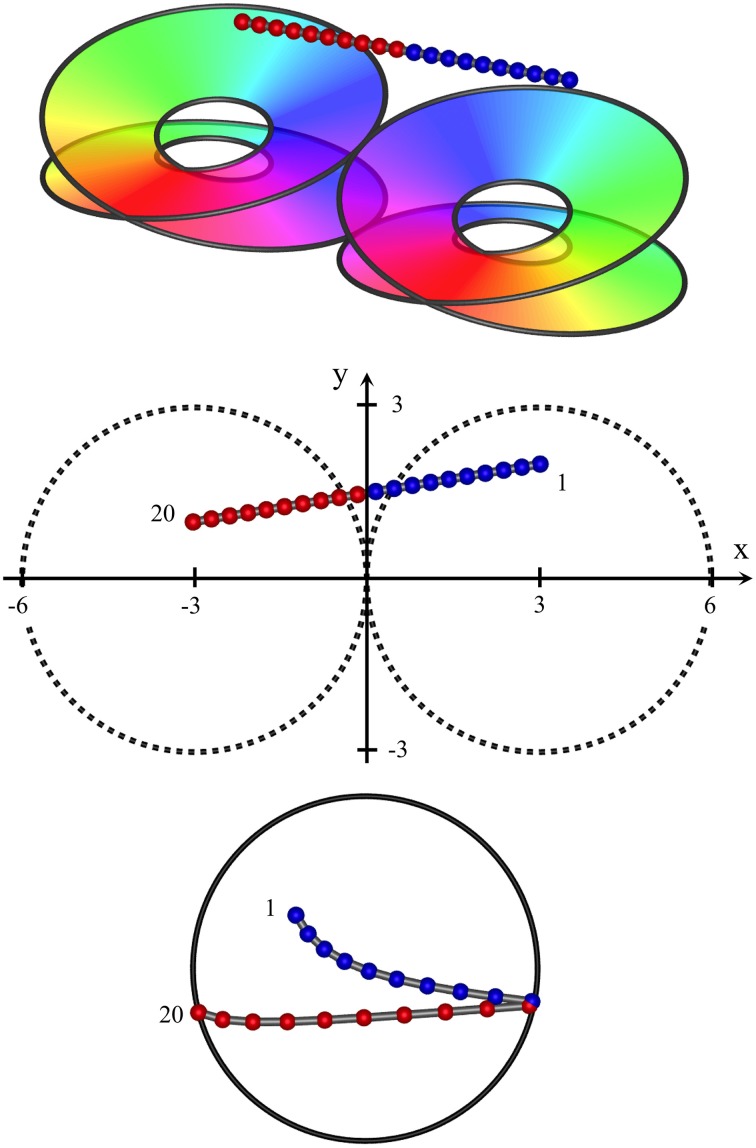
High horizontal passage of a recording electrode, crossing the junction of two adjacent macrocolumns, from *(x, y, z)* = (3, 2, 2) to (−3, 1, 2). RF centroids form columns of progressing sequentially in one direction, until the junction of the two macrocolumns is crossed, after which point the direction of sequential progression is reversed.

**Figure 5:**
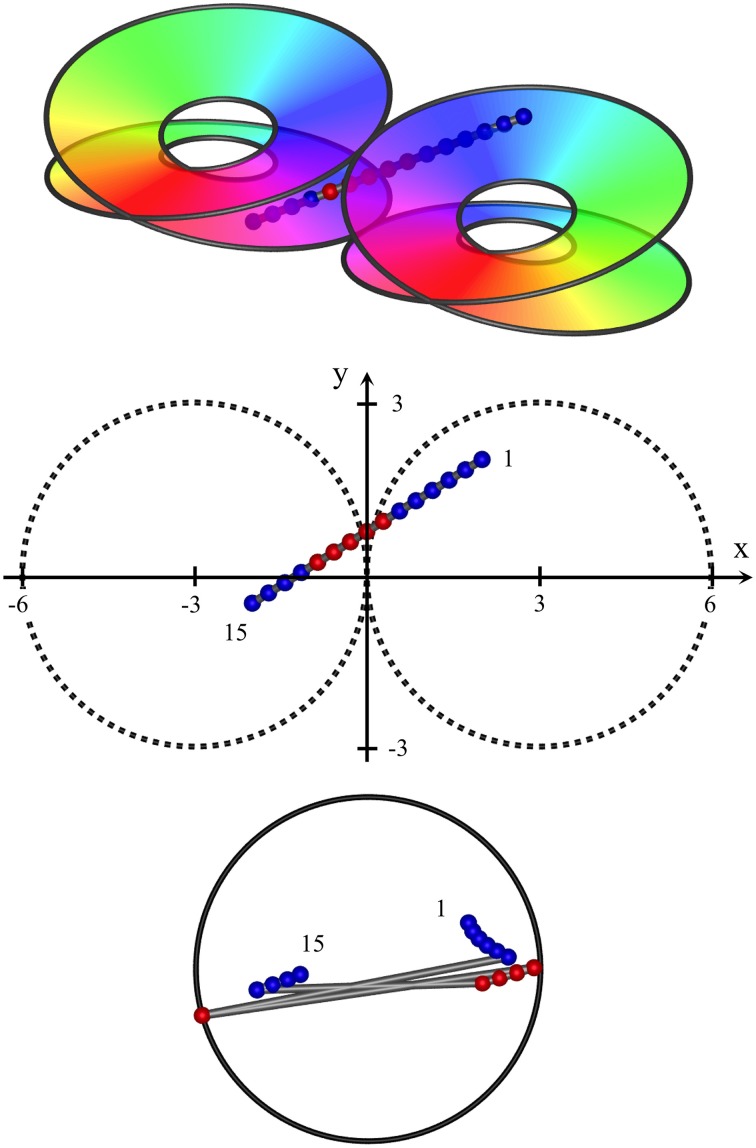
Oblique passage of the electrode, from (*x, y, z*) = (2, 2, 1) to (−2, −0.5, −1). The path of penetration both crosses the junction of adjacent macrocolumns and passes through zones in which clockwise and anticlockwise skeins of synaptic connections are poorly demarcated. Centroids of RFs associated with clockwise and anticlockwise skeins are entangled, but sequential progression of RFs associated the clockwise skein is still in the reverse direction to those associated with the anticlockwise skein.

The interface between the skeins can be represented by a 3D representation of a Möbius strip in Euclidean space, as is used in the top images of Figures 3–**5**, and is given by:

(5a) x=|p|cos (±ϑ+φ)

(5b) y=|p|sin (±ϑ+φ)

(5c) $$z_{M}=K\left|p\right|\sin\;((\pm \vartheta +\varphi )/2)$$

where *x, y* are Euclidean horizontal co-ordinates on the cortical surface, and *z_M_* is the depth of the interface between the skeins. ϑ is polar angle, ϕ the angle of orientation of the macrocolumnar map vs. the global coordinates; (ϑ + ϕ) ϵ [0, 4π], and ± indicates chirality of the macrocolumnar map. *K* determines the stretching of the skein interface in cortical depth.

Interpenetration and entanglement of the upper and lower skeins is presumed at the intermediate zone of cortical depth, between +*z_M_* to −*z_M_*, with greater separation of the skeins above and below.

The track followed by a recording electrode is:

(6) $$z_{D}=ax+by+c$$

where *z_D_* is the depth in the cortex reached by the probe, and *a, b, c* parameters of the track.

Thus, the positions at which maximum RF responses (subsequently treated as centroids of maxRFs) are discovered on the skin as the recording electrode advances follow a reverse mapping to that in Equation (4)—viz:

(7) $$\left|\sqrt{p}\right|\sqrt{p}\to \pm P$$

The sign (±) in Equation (7) is assigned positive if *z_D_* (*x, y*) > +*z_M_*(*x, y*), negative if *z_D_*(*x, y*) < −*z_M_*(*x, y*), and randomly assigned if +*z_M_* ≥ *z_D_* ≥ −*z_M_*. That is to say, according to where the cell lies in relation to the skeins.

Specifying Equation (7) for a pair of adjacent macrocolumns

(8a) $$\left|\sqrt{p-p_{1}}\right|\sqrt{p-p_{1}}\to \pm (P-P_{1})\text{ for }\mu_{1}(p)\ldots \mu_{trans}(p)$$

and

(8b) $$\left|\sqrt{p-p_{2}}\right|\sqrt{p-p_{2}}\to \mp (P-P_{2})\text{ for }\mu_{trans}(p)\ldots \mu_{n}(p)$$

μ_1_ … μ_*trans*_ and μ_*trans*_ … μ_*n*_ are positions on the line of advance of the probe Equation (6), and in cases where the track is transiting between the adjacent macrocolumns, the position of transit is designated μ_*trans*_. Positions *p*_1_ and *p*_2_ are the centers of the two maps, separated by a distance equal to the diameter of a macrocolumn. Adjacent macrocolumns each have representation, via the patchy connections, of extensive areas of the skin, and overlap of the skin area represented is considerable, and so to first approximation the skin area representations can be treated as identically centered, so that *P*_1_ ≡ *P*_2_.

The signs ± and ∓ in Equations (8a,b) carry the same meanings as in Equation (7) with the reversal ± to ∓ indicating the macrocolumns are so arranged that the entire Möbius mesh of one has opposite chirality to the other, forming mirror images, each of the other. For any pair of adjacent macrocolumns randomly selected, the axis of mirroring will be random relative to ϑ = 0, but the patterns of RF centroids arising with variation of verticality of the recording electrode would be little affected. So, for simplicity, only mirroring along the ϑ = 0 axis is considered in the next section.

### Positions of sequential RF centroids, predicted for electrode trajectories through S1

Figures 3–**5**, show expected results for the RFs sequentially discovered as an extracellular recording electrode passes through S1, from superficial to deep, through clockwise and anticlockwise skeins. Results vary depending on whether the electrode descends vertically, near-horizontally, or obliquely, and on whether the electrode remains within a single macrocolumn, or passes from one macrocolumn into its neighbor.

In each of the three figures, the top frame shows the skein interfaces in a pair of macrocolumns, viewed obliquely from above the cortical surface. Colors on the color wheel indicate equivalent polar angles on the clockwise and anticlockwise winding of the skeins. The passage of the recording electrode is shown as a line of dots passing through the cortex, and thus penetrating the connection systems from top to bottom. Red dots represent positions in which the patch projections are winding clockwise, and blue dots positions in which the projections are winding anticlockwise.

The middle frames show the pair of macrocolumns and the electrode in horizontal plane view. Although the individual macrocolumns are shown as circular, we have assumed columnar continuity at their margins.

The bottom frame shows the positions of the centroids of RFs of the neurons found sequentially by the penetrating electrode. (Supplementary movies provided with this paper show the sequential development, as the electrode is advanced.) RF centroids associated with positive-valued solutions on the right-hand side in Equations (8a,b) are marked red, while negative-valued solutions are marked blue. In the intermediate region where the skeins may mix, RF centroids are randomly assigned red or blue.

In these images no allowance has been made for cortical anisotropy, nor recording noise, and the bottom images have been normalized in scale, and centered.

Figure 3 shows results typical of a near-vertical electrode penetration remaining within a single macrocolumn (a type I penetration). Sequential RF centroids as the electrode descends are tightly clustered until a “jump” occurs in the sequence, as the electrode passes from one skein to the other, and a second cluster of RF centroids begins and persists until the cortex is completely penetrated. If the electrode track is slightly off vertical, the RF centroids may be less tightly clustered, and progress in one direction until the jump occurs, and in the opposite direction after the jump. If the electrode track passes through the less demarcated zone between skeins sequential centroids may jump back and forth between the clusters.

Figure 4 shows results typical of the passage of the recording electrode at an angle approaching the horizontal, remaining high enough in cortex to pass partly across the border of a pair of macrocolumns (a type II penetration), with only the upper skeins of both columns penetrated. In this case the sequential progression of RF centroids is in well-differentiated steps in one direction, then the reverse direction, as the junction between macrocolumns is crossed, encountering the mirror-image reversal of skeins in adjacent macrocolumns. Were the electrode to pass horizontally through deep cortex, a similar result (but with reversed positions of sequential RF centroids) would be obtained.

Figure 5 shows an intermediate result, also a type II penetration, where the electrode has passed obliquely through the system, encountering a substantial fraction of neurons lying in the poorly demarcated zones between skeins. A mixture of effects arises, all producing “jumps” backwards and forwards.

This continuum of outcomes permits model fitting to experimental data.

## Methods

All data were obtained by digitizing the original maxRF drawings of single neurons studied by Favorov, Whitsel and Diamond in 1980s at the University of North Carolina at Chapel Hill and published in Favorov et al. (1987), Favorov and Whitsel (1988a) and Favorov and Diamond (1990).

### Physiological RF recordings

Experimental methods are described for monkeys in Favorov and Whitsel (1988a) and for cats in Favorov and Diamond (1990). All experiments were conducted in accord with ethical standards then current at the host institutions.

A recording chamber was installed over a small opening in the skull over the S1 forelimb region of *Mucaca fascicularis* monkeys or domestic cats. Dura was removed under general anesthesia, surgical sites infiltrated with local anesthetic, then halothane anesthesia was discontinued, and the subjects were immobilized with gallamine triethiodide, placed on positive pressure ventilation, and EEG monitored.

Using electrolytically sharpened, glass-insulated tungsten electrodes in arrays, closely spaced radial penetrations each yielded extracellular records from 11–43 neurons. The effective sampling radius of the electrodes was estimated at <50 μm. Single units were isolated on the basis of the amplitude and the shape of the recorded action potentials; multi-unit recordings were excluded. For each isolated single unit demonstrated to receive input from the skin, the modality (hair movement, skin contact, or movement over the skin) of the effective peripheral stimuli, and the rate of adaptation of the neuronal response to maintained adequate stimulation were determined. The stimulus that evoked the most vigorous response when applied to the most sensitive part of the RF was used to map RF boundaries. Cells responding primarily to deep tissue stimuli were excluded.

Maps of skin RF boundaries were transferred to a standard forelimb drawing and subsequently digitized for analysis. Electrolytic lesions were placed in selected penetrations to enable reconstruction of electrode tracks in the serial histological sections prepared for each brain.

Centroids of RFs were computed from their boundaries, and the centroids plotted on standard forelimb drawings. In a few instances divided RFs were found, that could not be defined within a single enclosing boundary, so in these cases the centroid was calculated for the overall RF.

### Preliminary identification of RF centroid patterns before model fitting

In each dataset, progressions of the centroid positions in the order they were recorded were noted. Three typical classes of results were found, corresponding to those described by Figures 3–5, with an overlay of spatial noise.

1. Cases in which there is one cluster of points, and a few outliers or a smaller cluster. For model fitting, centroids in the first cluster (in numerical order of the neurons recorded) were designated red, and those of the outliers/second cluster blue, in accord with colors ascribed in Figure 3.
2. Cases in which there is a clear sequence of points going continuously in one direction, then reversing back in the other direction. The initial direction of progress of the centroid progress in order was designated red, the reverse progress blue, in accord with the colors ascribed in Figure 4.
3. Cases in which the points in sequence “jump” back and forth irregularly, without distinct clusters. In accord with Figure 5, points following a jump in one direction were designated red, jumps in the opposite direction blue.

Since these data were to be fitted to a theoretical model utilizing a maximum of two adjacent macrocolumns, the model can be fitted only to datasets in which there is a maximum of three distinct clusters or sequential runs of red and blue points in a single direction. More than three clusters or runs were apparent in some datasets obtained in a single electrode penetration. Then the dataset was split into sections each of which could be fitted to the model individually, and these subsets are subsequently referred to as “splits.”

A few datasets were obtained in which the centroids progressed in jumps both proximal and distal as well as ulnar and radial, preventing orderly numbering. These data were set aside, considered as artefactual or arising from situations outside the theoretical model to be used in fitting—e.g., they might arise from electrode penetration of cortical areas on junctions between three macrocolumns.

**Figure 6:**
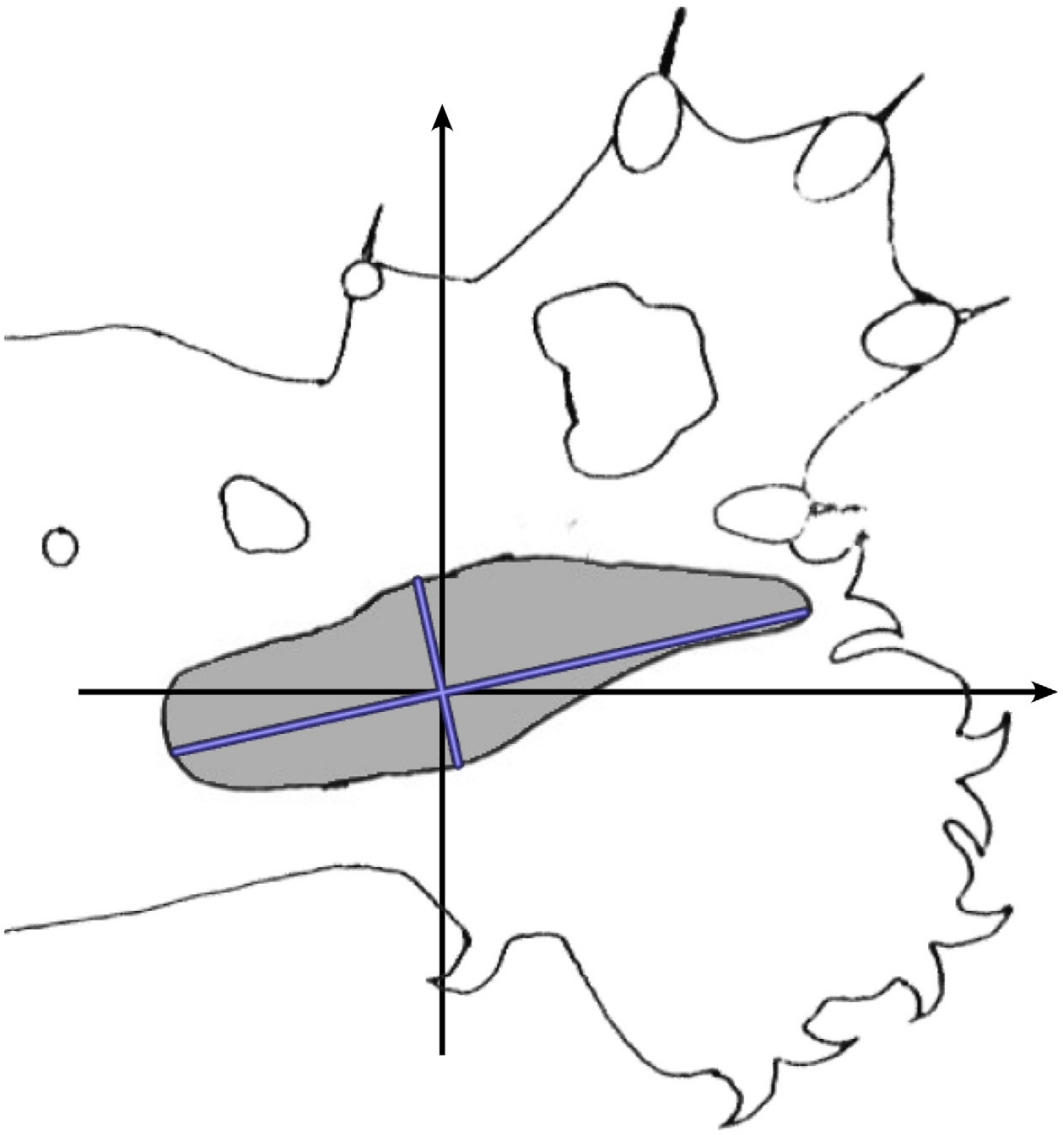
**Calculation of cortical anisotropy for an RF**. Against an outline of the cat's standard forelimb with reference axes, the blue lines indicate the longest and broadest axes of the RF.

Calculations were then made to allow for effects of cortical anisotropy. Figure 6 shows a typical RF against a cat's forepaw outline. The size and elliptical shape of the RF reflects the anisotropic representation of the skin surface in S1 (e.g., Kaas et al., 1979) in which cortical representation is of roughly inverse size to skin extension. Length of the longest axis of each RF and its angle to a reference frame on the animal's limb, and length on the broadest axis orthogonal to the long axis, were measured and averaged over the RFs in a dataset. Subsequently the average anisotropy for the dataset was applied to the spatial distribution of theoretical points at each trial of fit.

### Model fitting

Best fit of theoretical model to experimental data was found for each experimental dataset of *n* RF centroid positions, by random matching of experimental data to results possible according to the theoretical model. This was done by:

- Translating the experimental centroid points so that their combined center-of-mass was located at the origin, and normalizing the scale of the translated plot to the unit circle.
- Computing sets of theoretical RF centroid point sets *P*_1_ … *P_n_* —each set associated with one of all possible probe tracks, and of all equidistant neuron positions from μ_1_ … μ_*n*_, along each possible subset of each track—thus choosing random neuron point sets over the full range of possibilities for parameters *a, b*, and *c*, in Equation (6), and generating theoretical centroid point sets for each of these, in accord with Equations (8a,b).
- Transforming each theoretical centroid point set to eliminate the effects of cortical anisotropy, as described in the prior section, normalizing their scaling and translation as for the experimental data and randomly rotating each set.
- For each of these anisotropy-corrected, normalized, scaled and translated theoretical point sets, calculating the sum of squared deviations of theoretical *P*_1_ … *P_n_* from their experimental equivalents in each trial.
- Selecting the best fit. For each random match, goodness of fit was calculated as RMS noise/signal ratio—that is, the sum of squared distances of separation of each of the matched theoretical and experimental centroids was divided by the sum of squared distances of the experimental centroids from the origin, and the square root of the result then taken.

For each dataset 120 billion random matches were tried, and their best-fit results tabulated.

### Construction of nested RFs

The RFs were superimposed and the number of RFs overlapping at each position on the standard forelimb counted, to obtain local maximum counts. Local maxima thus indicated nest centers, as those with the largest number of contributing RFs. We then superimposed upon each of the centers all those RFs within which that center lay, thus obtaining all nested RFs for the dataset. Each nest was plotted on the standard forelimb outlines, and the color spectrum from red to blue used to indicate their density of overlap.

## Results

### Datasets and exclusions

From the existing monkey data, we excluded experiments in which RF outlines were confined to fingers, as this distribution made calculation of RF centroids problematic. From cats we excluded datasets in which a large fraction (about 50%) of RFs were non-sequential, because interposed neurons had not had RF boundaries recorded but were used for a different purpose. We retained one dataset (CAT8615p1) that had only two neurons' recordings missing in sequence, and in that instance interpolated blank, unweighted positions during model fitting.

The only other exclusions were two monkey datasets in which the plot of centroids progressed in steps proximal and distal as well as radial and ulnar, preventing orderly numbering, and were therefore considered artefactual or obtained from an irregular cortical area.

This left 22 datasets, composed of either complete sequences passing through the cortex (10 datasets) and 12 “splits,” divided as stated in Methods (6 complete sequences, each divided into 2 split datasets).

The 22 datasets were obtained from 7 animals (3 cats and 4 monkeys, 7 datasets from cats and 15 from monkeys) providing RF outline data from 310 neurons.

### Electrode track histology

Penetrations were never perfectly perpendicular to the cortical surface. A 200 μm advance of the electrode resulted in an average displacement of 50 μm in the horizontal plane. In comparing experimental data with the theoretical model, we have made no attempt to directly relate histological measurement of obliquity of the electrode track to the outcomes, because track variation was markedly overshadowed by the effect of part of the limb in which the measured RFs were clustered. If located distally in the limb, results approximated expectation for a vertical electrode passage. If in the proximal limb, the results approached expectation for oblique or almost horizontal electrode passages. That is, cortical anisotropy, by affecting relative size of cortical representation of equal skin areas, predominated in determining the apparent obliquity of the electrode track, because the larger the cortical representation the less the apparent lateral drift across the skin representation.

### Representative results of model fitting

Figures 7–12 show representative results. Complete analyses of all datasets are supplementary to this paper, and are commented on further at the end of this section. An example from each of cat and monkey is shown, for each of the three types on the continuum of possibilities forecast in Figures 3–5.

**Figure 7:**
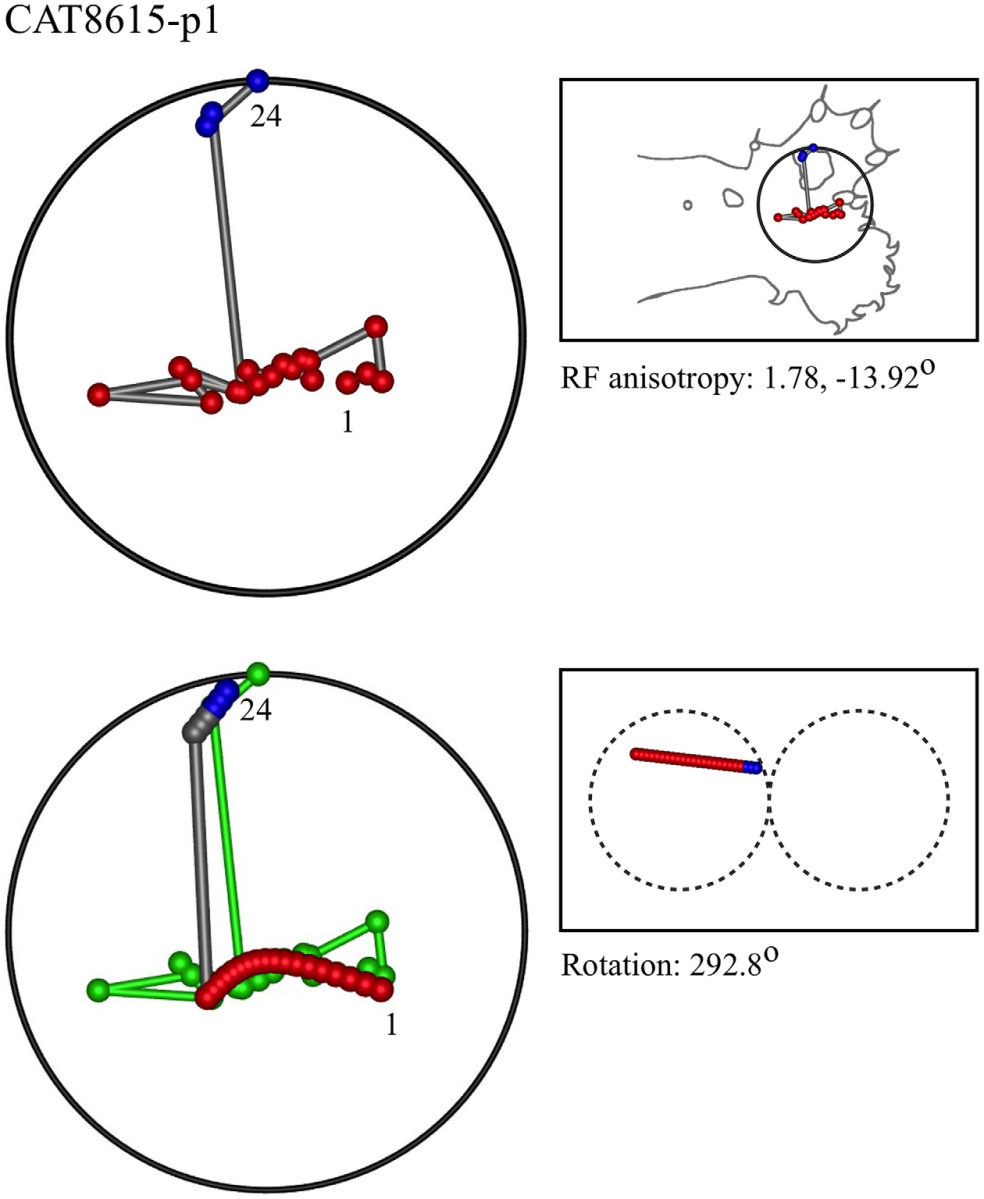
**A “vertical” penetration of cortex in a cat. Top Left**: Experimental data—centroids of RFs, red and blue colors indicate hypothetical position within Möbius organization of macrocolumn. **Top Right**: RF centroids on cat forelimb outline. **Bottom Left**: Best fit of theoretical model to experimental data. Background green line is outline of experimental plot. **Bottom Right**: Theoretical construction of passage of recording electrode, on plane view of cortical surface.

**Figure 8:**
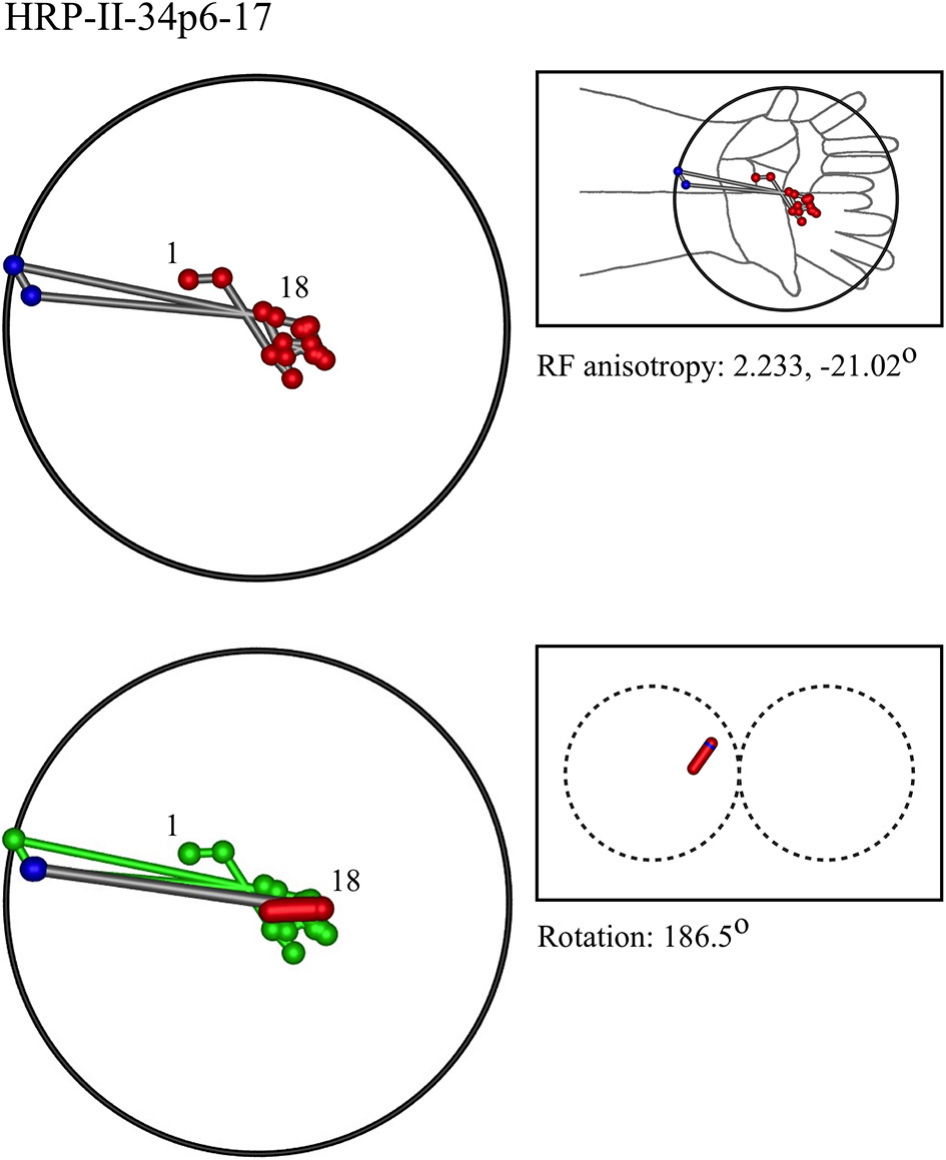
**A “vertical” penetration of cortex in a monkey**.

**Figure 9:**
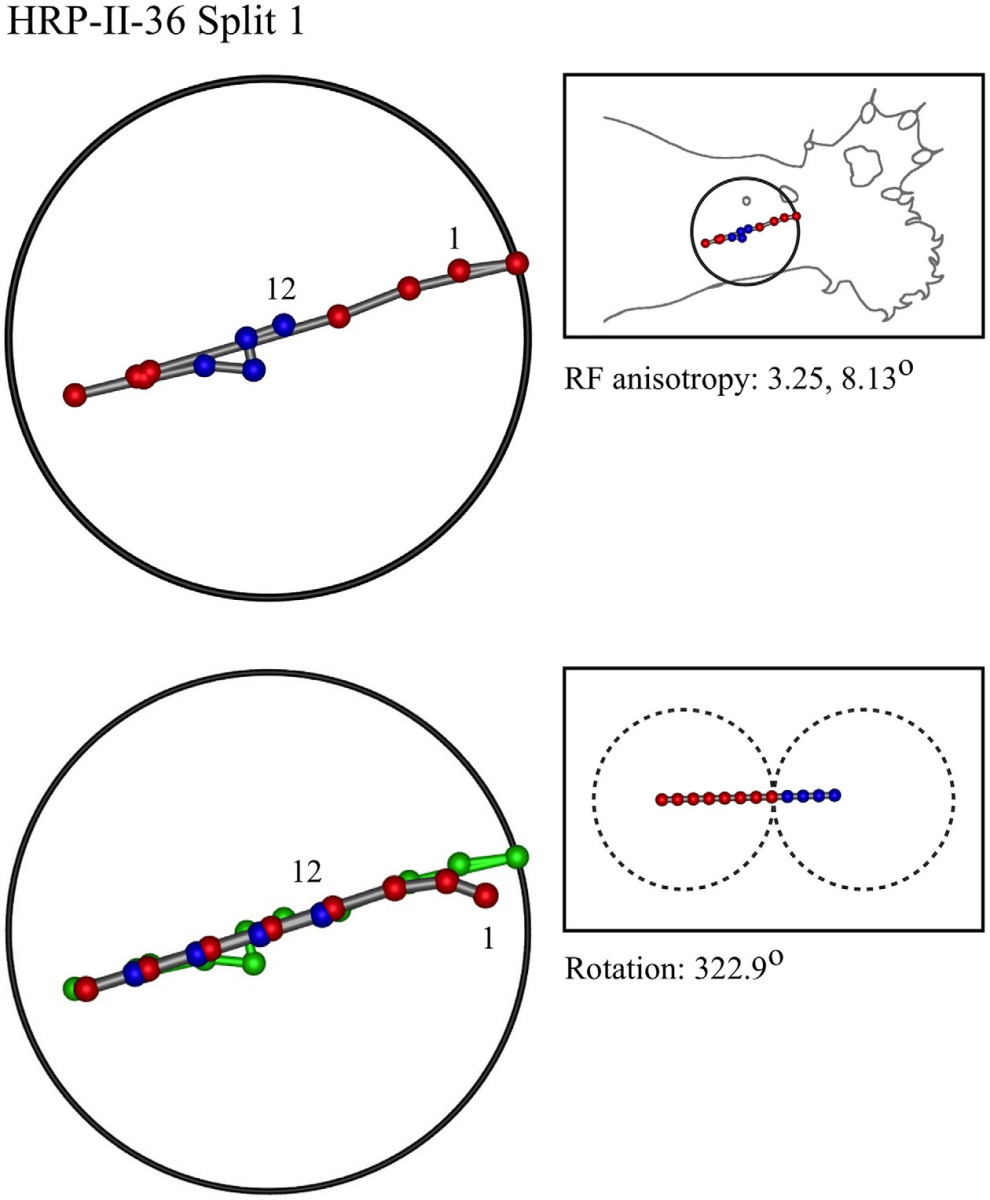
**A “horizontal” penetration in a cat**.

**Figure 10:**
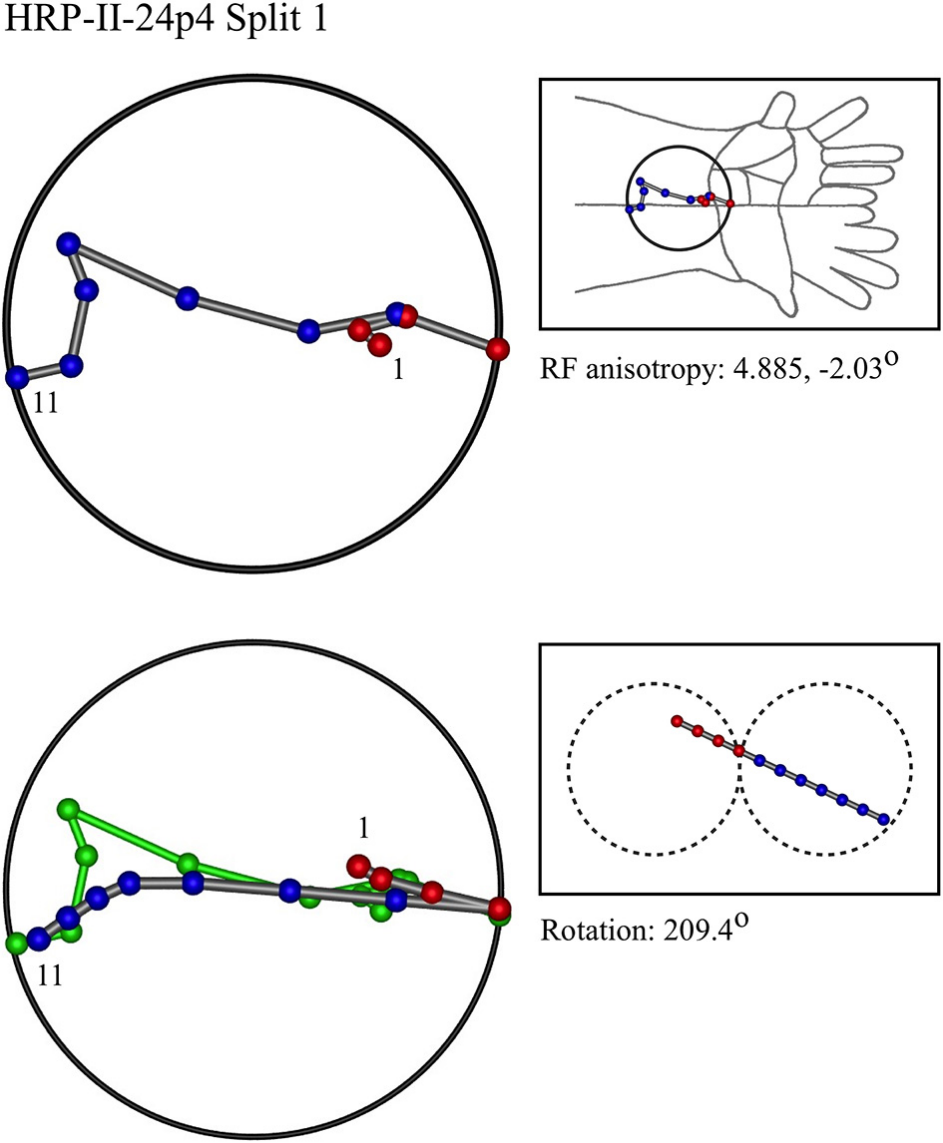
**A “horizontal” penetration in a monkey**.

**Figure 11:**
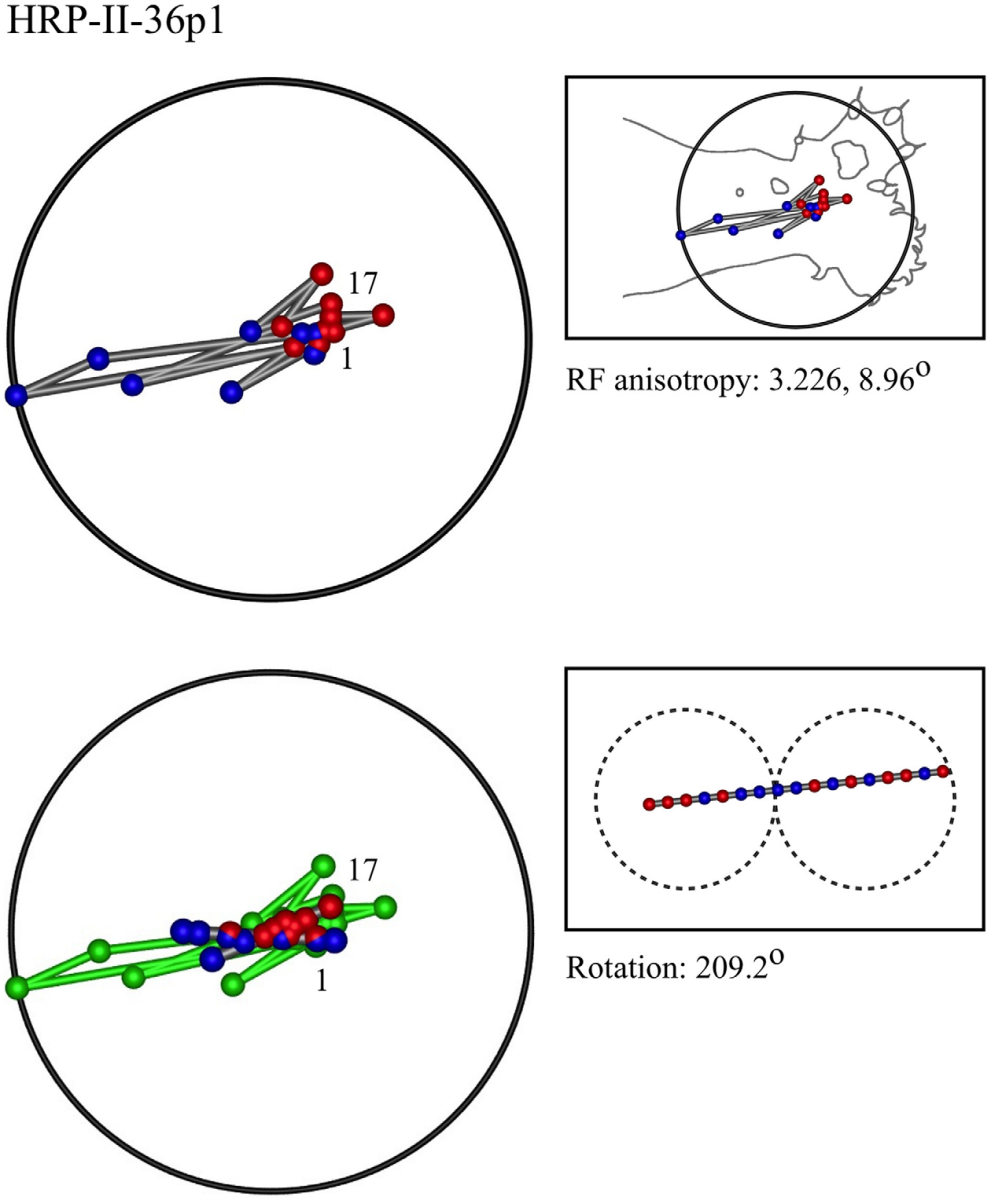
**An “oblique” penetration in a cat**.

**Figure 12:**
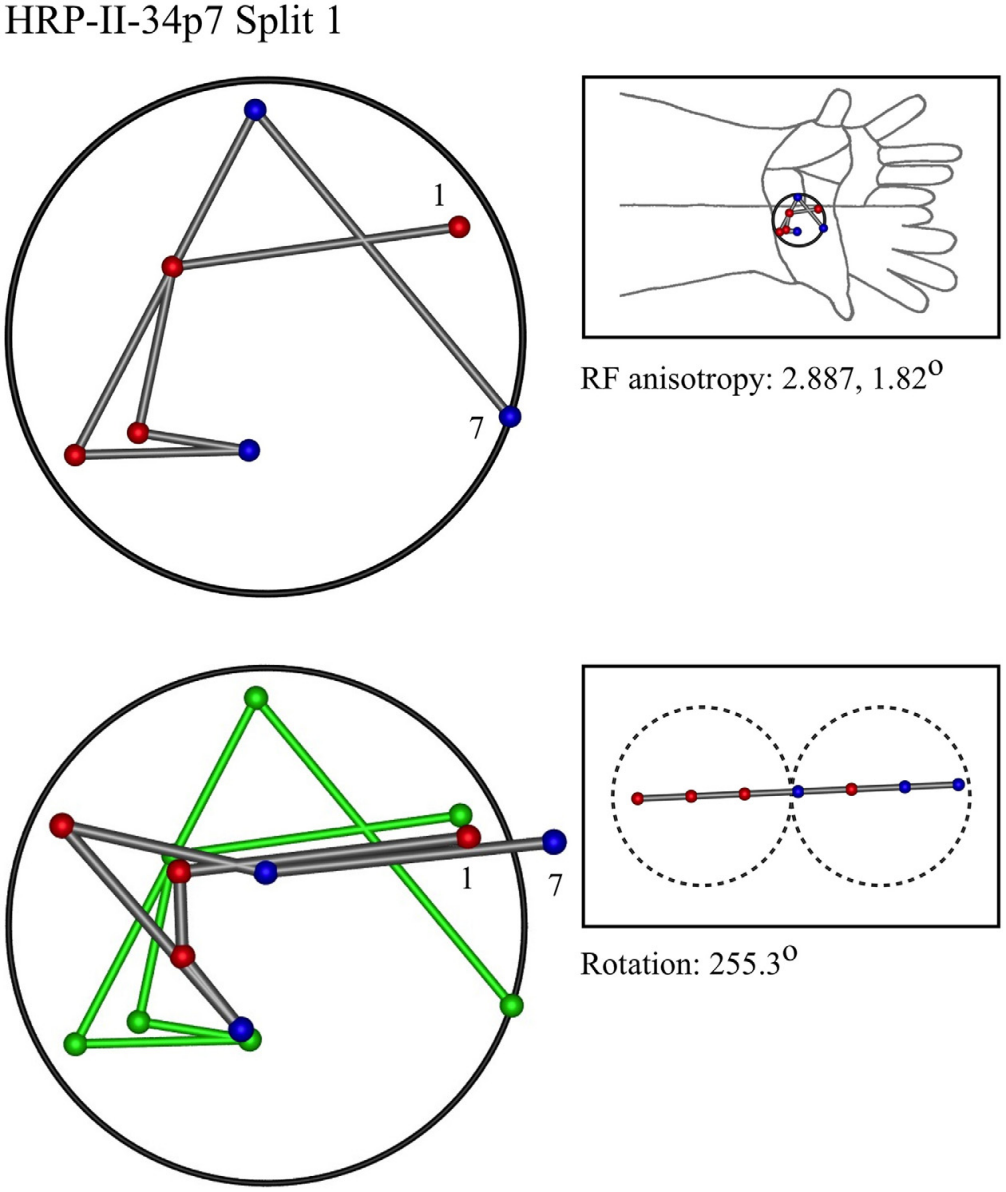
**An “oblique” penetration in a monkey**.

In the top left corner, each figure has the label given the data in the original experiments of Favorov et al. Top left is the plot of RF centroids, and top right the plot is referred to the cat or monkey forelimb outline. Centroids are colored red or blue according to the conventions described in Methods. Bottom left is the best-fit theoretical result, with, shown in green, the corresponding connected line through the experimental centroids. Bottom right, the two circles show the pair of theoretical macrocolumns and needle track in plane view.

The values shown in each figure as “RF anisotropy” give the average ratio of the long to the short axes of the RFs in a dataset, and the angle of the long axis to the reference axes. “Rotation” is the angle through which the theoretical best fit was rotated during matching of model to experiment. It is, therefore, a measure of the orientation of the hypothetical local map.

RMS noise/signal ratios (goodness of fit) ranged from 0.23 to 1.06, the lower values tending to occur with more proximal RF datasets. Mean RMS noise/signal over all datasets was 0.62. Tabulated results are provided as Supplementary to this paper.

Considerations relevant to interpreting these results are as follows:

- No other theoretical model exists, to the best of our knowledge, which can explain the positions of the RF centroids found in these experiments. The earlier segregate analysis of Favorov et al., cited in the Introduction, is based on different experimental methods, and is related to the RF nests described in a subsequent section.
- The experimental datasets vary markedly in their apparent orderliness, but are not random patterns. Each dataset resembles one or other of the patterns predicted in Figures 3–5.
- The theoretical model finds best fits in accord with the patterns predicted in Figures 3–5, and the model could not be fitted adequately to most geometric configurations—with the proviso that interference by spatial noise, and ambiguity in some centroid configurations makes alternative fits of the model, with similar goodness of fit, possible in some cases. Trials were made in which datasets where fitted with red and blue designations other than as described in section Preliminary Identification of RF Centroid Patterns Before Model Fitting, to illustrate these properties and limitations (See Supplementary Material).
- There is mutual consistency of the findings of the model-fits across separate datasets. Over all datasets, including those provided as supplementary material, the following trends were apparent:
  - In 9/22 datasets, a pattern of RFs similar to the “vertical” outcome exemplified in Figure 3 was found, all with a type I best-fit configuration. In 8 of these, the RFs were located in the distal limb. This is the type of outcome expected in distal skin areas, which have larger cortical representation.
  - In 6/22 datasets, a pattern of RFs similar to the “horizontal” outcome exemplified in Figure 4 was found, all with a type II best-fit configuration. In 5 of these the RFs were located in the proximal limb. Conversely, this is the type of outcome expected in cortical areas with smaller cortical representation.
  - In 7/22 datasets, a pattern of RFs similar to the “oblique” outcome exemplified in Figure 5 was found, 5 of which were of type II, and one of type I. All were in middle dispositions on the limb, except for the type I instance, which was proximally located. They are thus deployed in the skin area that has intermediate levels of cortical representation.
  - Where sequentially discovered RFs in a single electrode penetration were “split” so as to be able to be fitted to the two-macrocolumn theoretical model, then in 4/6 cases, a type II fit was found in the first “split” followed by a type II fit to the second split, and in 2/6 a type II fit was followed by a type I fit—showing the model gives consistent results in all cases, indicative of continued horizontal straight-line passage of the electrode.
  - Best-fit noise/signal ratios tended to increase proximally to distally. This is also consistent with expectation for the effects of cortical anisotropy, since larger cortical representation compresses the distribution of the RFs, without reducing recording uncertainty (spatial noise) regarding the exact position of the receiving neuron.
  - The mean RMS noise/signal of 0.62, given the effective electrode sampling radius of <50 μm, implies mean horizontal drift of the recording electrode in passage through some or all of the cortical depth, was approximately 80 μm. This appears close to actual electrode drift.

### Comparison of Möbius ordering with nested RFs

Nested RFs were obtained for all 22 datasets, and also for all 16 complete full-cortical thickness sequential recordings, so that nests in the “splits” were considered both combined and separately.

In one dataset we found 4 nests, in one 3 nests, in four 2 nests, and only 1 nest in all others. The datasets that had been divided into “splits” for model fitting, in all cases but one, had only a single nest, and the exceptional case was composed of 3 nests, 2 in one split, and 1 in the other.

There was no one-to-one correspondence between nested RFs and Möbius model constructions. However, where multiple nests were found in a dataset, the RF centroids of the set were relatively distally located on the limb—the first and largest nest more proximally, and the smaller nests more distally on the cat's paw, or on individual fingers of the monkey. In the proximal forearm of both species, large single nests predominated. The proximal/distal differentiation into fewer and more nests corresponded to the proximal/distal trend of type II vs. type I model fits.

**Figure 13:**
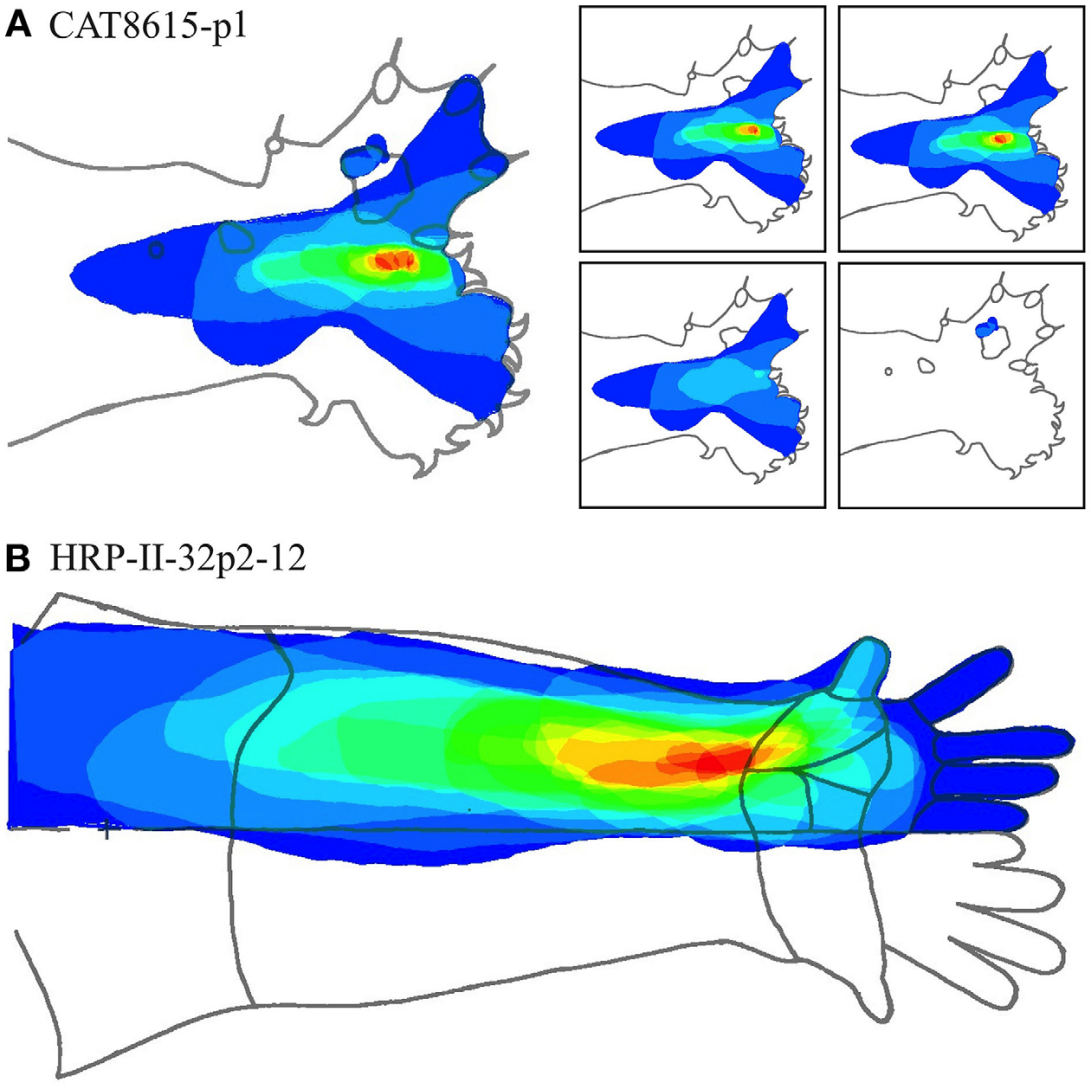
**(A)** Nested RFs in the distal paw. (Type I -single macrocolumn). **Left**: Superposition of all RFs in a dataset. Color spectrum represents density of overlap of RFs; red most dense. **Right**: Separate RF nests. Retaining the same color coding in the complete dataset, these are arranged in order of the number of RFs in each nest—15, 14, 4, and 3, respectively. **(B)** RF nest in the forearm. (Types II—three macrocolumns). All RFs surround a single centroid.

Figures 13A,B show two cases at these extremes.

Figure 13A shows the entire overlapping RF set, and the four nests obtained from this dataset. Two of the four nests are each contained within the largest nest, and one nest falls outside all the others. Comparison with Figure 7, which is the same dataset, shows the modeling result was Type I, vertical—that is, the electrode appears to have passed down through one hypothetical macrocolumn, penetrating first the “clockwise” and top skein of connections, and then the “anticlockwise,” bottom skein, as shown by the red and blue dots respectively. It can be seen that the nest that overlapped with no other nest, corresponds to the anticlockwise, bottom, skein.

Figure 13B shows a result from the forearm that is all one nest. The Möbius model had to be fitted in two “splits” implying the dataset was obtained from penetration of three macrocolumns.

## Discussion

As originally recognized by Favorov et al., the RFs do not conform to an homotypic map, nor are they wholly random, nor a mixture of homotypic map with uniformly distributed spatial noise. Other than the present model, we know of no other candidate geometry that could explain these results. The expected variant patterns with path of descent of the recording electrode were all encountered in roughly equal numbers, average noise/signal ratios conformed to expectation for spatial noise of recordings, other expectations arising from the effects of position on the limb, and cortical anisotropy were consistently obtained, including mutually consistent modeling of recording sequences that were split for analysis.

Since the theoretical model requires optimization of several parameters, and normalization of scale, rotation and translation, it might be objected that the model could fit any dataset whatsoever, but for the reasons given in association with Results, this objection does not hold.

We propose that the Möbius structure reflects the antenatal organization of synaptic connections, and the RF nests reveal the development, post-natally, of learned functional connections superimposed over the antenatal connection system, in the manner of a palimpsest. Since neurons receiving inputs from an RF nest all receive spatially correlated inputs, neurons in different nests have partially correlated inputs if the nests overlap, while those neurons each at the common center of nests each have unique inputs distinguishing them each from the others. Neurons receiving inputs from non-overlapping nests have no common input. That is, non-overlapping nests represent cells responding to principal components of input spatial cross-correlation, and the RF shapes represent the spatial response tuning of the cortical neurons—analogous to the frequency-domain spatial and temporal tuning seen in V1 neurons, reported by Issa et al.

The applicability of the Möbius model to both V1 (where anatomical macrocolumns are apparent) and S1 (where they are not) supports the notion that the principle of organization may be general throughout the cortex. All sensory data reach the cortex as some form of primary spatial map, regardless of how the sensory material may be transformed in space, time, or frequency on the sensory pathway, and consequently might be similarly organized for all modalities. We have also argued previously (Wright and Bourke, 2013) that similar principles may enable mutual self-organization of synapses and interactions between cortical areas.

Continued successful application of this model would enable reformulation of the column concept, not as an anatomical entity dependent on the positions of cell bodies and the overall deployment of axons, but as a functional entity, constructed from the patterning of synaptic connections, whether or not the cells composing the system are separated into obvious columns, or intertwined more completely. This places emphasis much more strongly on horizontal connections (Boucsein et al., 2011) and moves away from Mountcastle's minicolumn as a structural entity. The minicolumn, in this view, is the necessary result of order in the horizontal connections, and in this revised concept, the minicolumn does not retain response identity throughout surface to depth of the cortex.

Further experimental testing of the model appears practicable. Using other sensory modalities, testing, similar to the present work, using multiple or single unit analyses, and electrode trajectories exploring small cortical areas, could seek to determine positions of sudden “jumps”—the breaches of continuity, or reversal of RF centroids, that are the crucial model signature. These are likely to be found most clearly with electrode trajectories very close to vertical, and nearly horizontal. Primary sensory cortices of all modalities might be thus explored, as may other cortical areas for which the sources of inputs can be defined. More accurate quantitative modeling would need to take into account the local distributions of local cell axonal lengths, and the spatial resolution in inputs, relating these to the relative clarity of definition of anatomical columnar definition. Direct anatomical demonstration of Möbius-strip-like organization of synaptic connections would place high demands on the capacity of existing techniques to specify detailed synaptic connections by the thousands. The task is made even more difficult where primary embryonic order is overwritten during subsequent learning, as must be the case with continuing pre-synaptic pruning and apoptosis in the early post-natal period. The discovery, made by Markram and colleagues, of “lego” assemblies of strongly connected pyramidal cells that form interdigitated networks (Perin et al., 2011) may reflect the establishment of the cross-correlations revealed in RF nests, and the “lego sets” might also be regarded as subassemblies capable of connection into Möbius meshes. The very large-scale analysis of detailed synaptic connectivity intended in the Human Brain Project and related proposals (Kandel et al., 2013) might definitively confirm or refute the model.

Theoretical development of this connectivity concept might exploit the long-sought advantages of decomposition of the cortex into modular information processing units. There are other implicit and attractive properties. The model explains how neural connections, self-organized into ultra-small-world configuration, may approach a maximum for speed and energy-efficiency, and indicates how spatio-temporal order may underpin the organization of signal traffic and learning, since local maps within each macrocolumn have inputs organized in accord with distance and delay from central points—of which the OP singularity is archetypal. The principle of synaptic resource competition, upon which the model is based, also suggests a form of synaptic metabolic entanglement, permitting signal processing complexity, and synaptic information storage, to approach theoretical maxima.

### Conflict of interest statement

The authors declare that the research was conducted in the absence of any commercial or financial relationships that could be construed as a potential conflict of interest.
