# Supplementary material for: Möbius-strip-like columnar functional connections are revealed in somato-sensory receptive field centroids
Source: Front Neuroanat. 2014 Oct 31;8:119. doi: 10.3389/fnana.2014.00119 (PMC4215792; doi:10.3389/fnana.2014.00119)
Supplement: Supplementary file 1 [file SupplementaryMaterial.ZIP › Supplementary/All RF Centroid Plots and Model Best Fits/CAT8615-p1.pdf]

CAT8615-p1

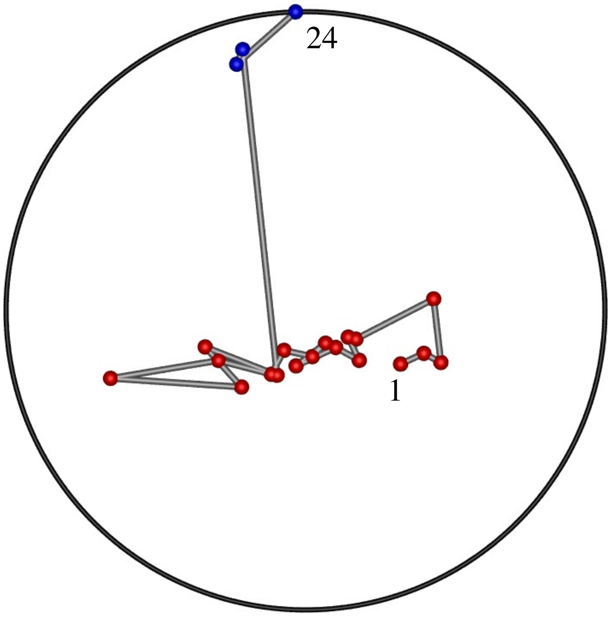

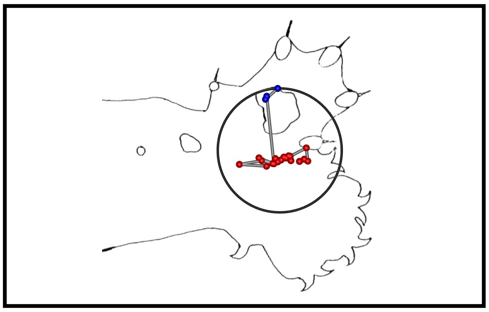

RF anisotropy: 1.78, -13.92^{o}

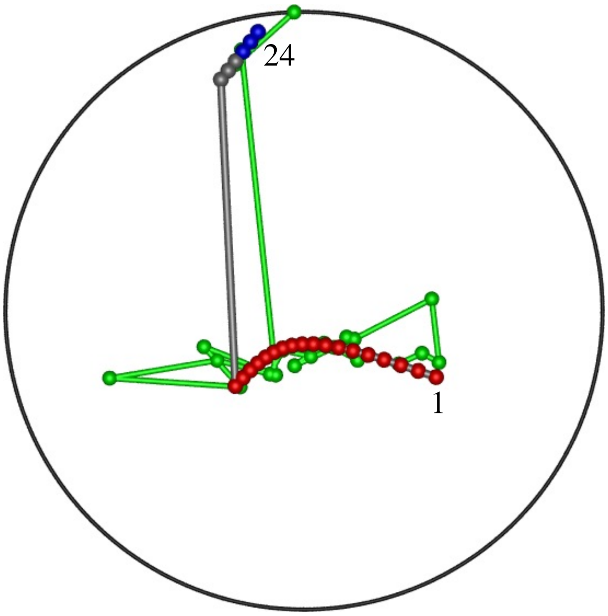

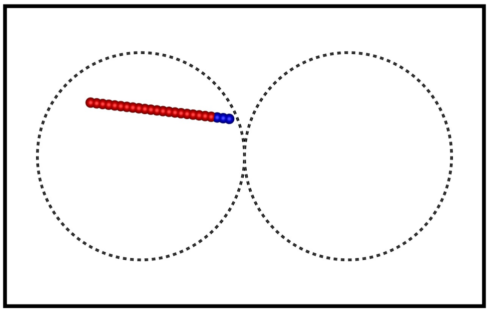

Rotation: 292.8^{o}

-------------------000+++
Type 2, N = 24, theta: 353.3, yinter: 1.030, std: 0.000, mu: 0.090 -> 0.440
zrotate: 292.8, scale: 0.750, stretch (r: 1.780,theta: -13.92), dxy: (-0.800,0.740)

CAT8615-p1/processed
Centroid: (1137.87,702.153)
-------------------000+++
r average: 0.56158, std: 0.180924
a average: -13.9169, std: 28.9104
