# Supplementary material for: Möbius-strip-like columnar functional connections are revealed in somato-sensory receptive field centroids
Source: Front Neuroanat. 2014 Oct 31;8:119. doi: 10.3389/fnana.2014.00119 (PMC4215792; doi:10.3389/fnana.2014.00119)
Supplement: Supplementary file 1 [file SupplementaryMaterial.ZIP › Supplementary/All RF Centroid Plots and Model Best Fits/CAT8615-p2.pdf]

CAT8615-p2

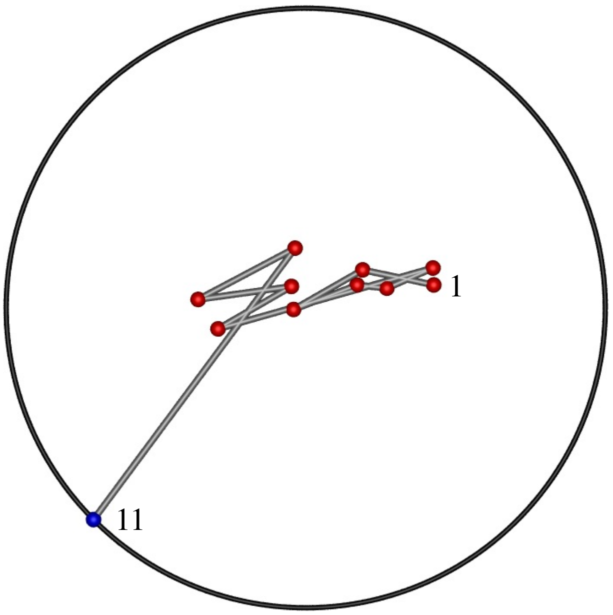

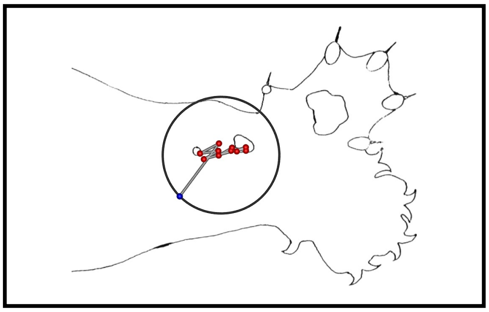

RF anisotropy: 1.655, 11.59^{o}

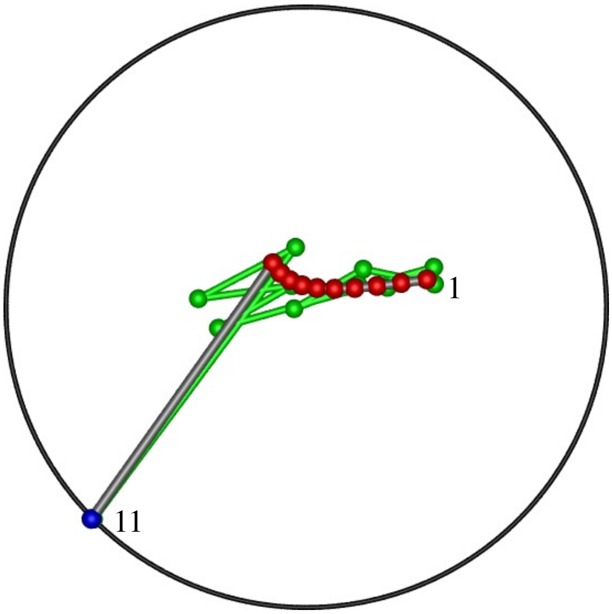

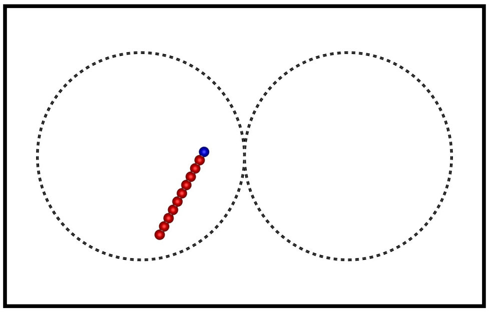

Rotation: 291.4^{o}

----------+
Type 2, N = 11, theta: 241.8, yinter: 2.310, std: 0.000, mu: 0.160 -> 0.690
zrotate: 291.4, scale: 0.720, stretch (r: 1.655,theta: 11.59), dxy: (-1.270,-0.780)

CAT8615-p2/processed
Centroid: (932.955,693.797)
----------+
r average: 0.604311, std: 0.203789
a average: 11.5888, std: 36.8378
