# Supplementary material for: Möbius-strip-like columnar functional connections are revealed in somato-sensory receptive field centroids
Source: Front Neuroanat. 2014 Oct 31;8:119. doi: 10.3389/fnana.2014.00119 (PMC4215792; doi:10.3389/fnana.2014.00119)
Supplement: Supplementary file 1 [file SupplementaryMaterial.ZIP › Supplementary/All RF Centroid Plots and Model Best Fits/CAT_874_p_2.pdf]

CAT_874_p_2

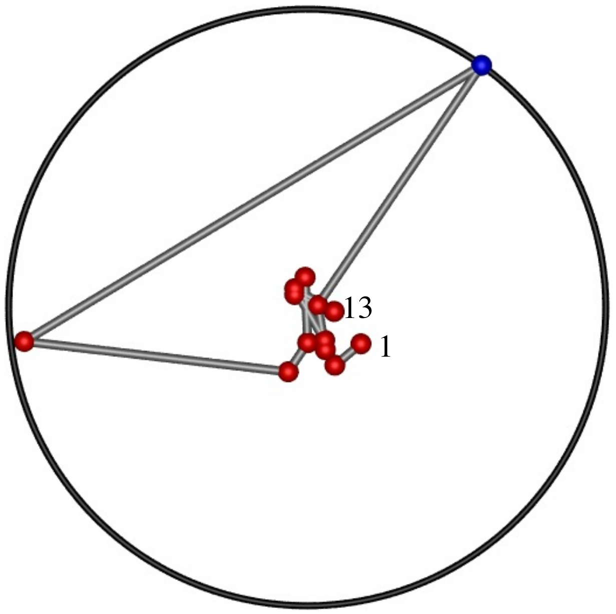

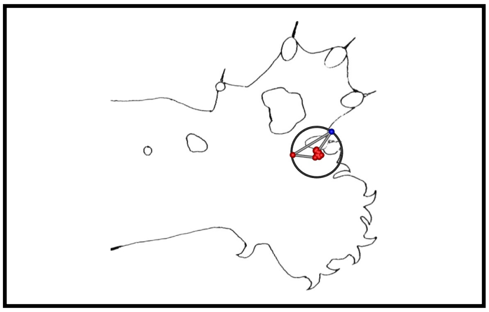

RF anisotropy: 1.385, 41.77°

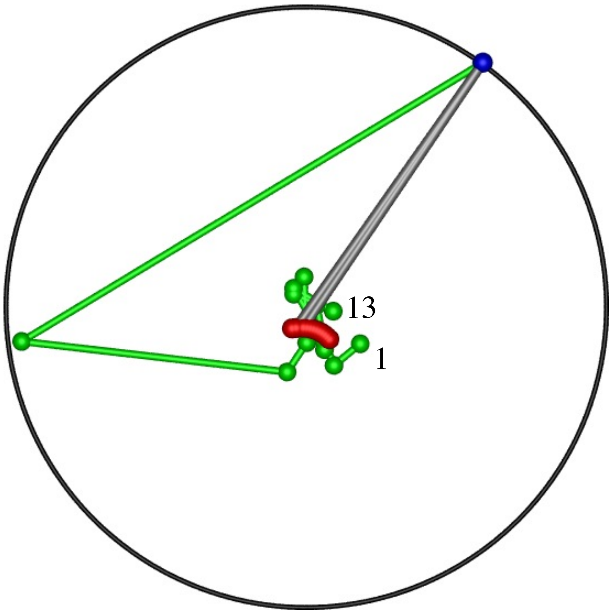

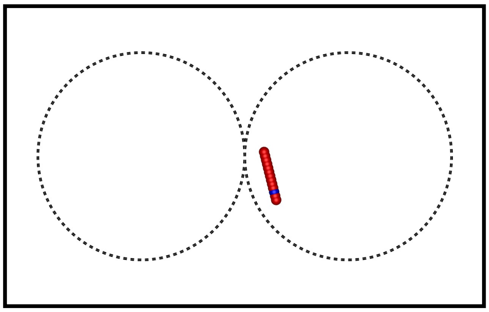

Rotation: 106.8°

----------+--
Type 2, N – 13, theta: 284.1, yinter: 2.350, std: 0.000, mu: 0.310 > 0.690
zrotate: 106.8, scale: 0.500, stretch (r: 1.385,theta: 41.77), dxy: (0.850,1.120)

CAT 874 p 2/processed
Centroid: (1194.35,698.864)
----------+--
r average: 0.721826, std: 0.243601
a average: 41.7665, std: 36.8814
