# Supplementary material for: Möbius-strip-like columnar functional connections are revealed in somato-sensory receptive field centroids
Source: Front Neuroanat. 2014 Oct 31;8:119. doi: 10.3389/fnana.2014.00119 (PMC4215792; doi:10.3389/fnana.2014.00119)
Supplement: Supplementary file 1 [file SupplementaryMaterial.ZIP › Supplementary/All RF Centroid Plots and Model Best Fits/HRP-II-24p2.pdf]

HRP-II-24p2

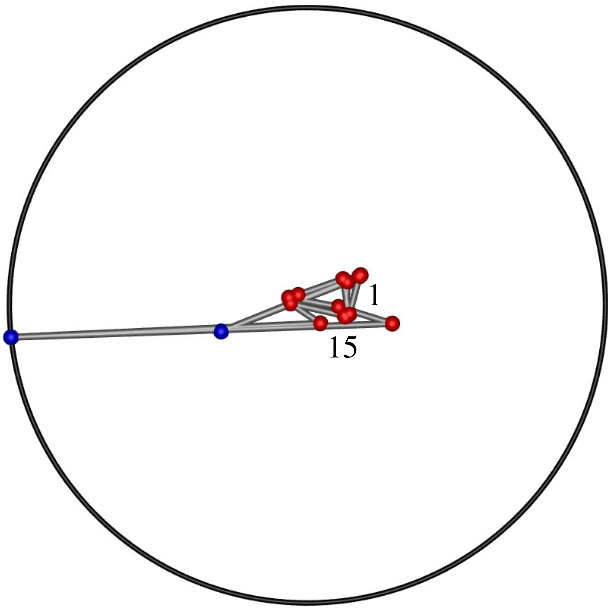

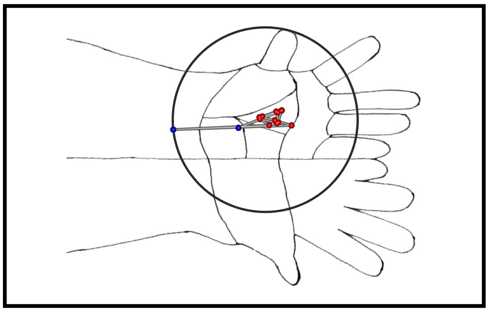

RF anisotropy: 1.991, 4.65^{o}

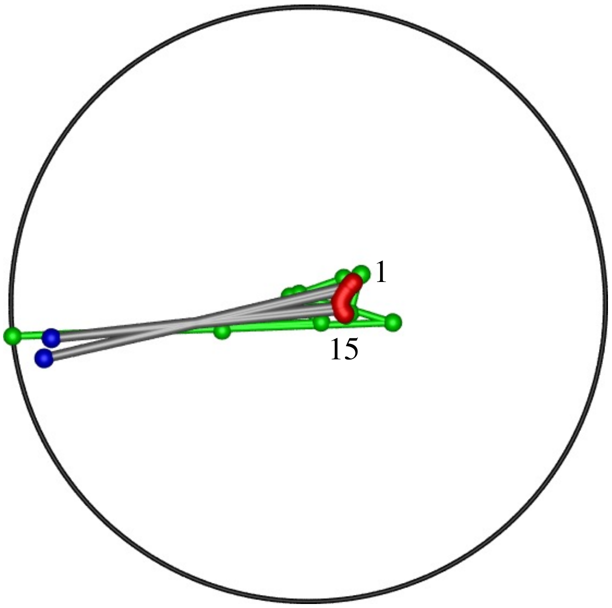

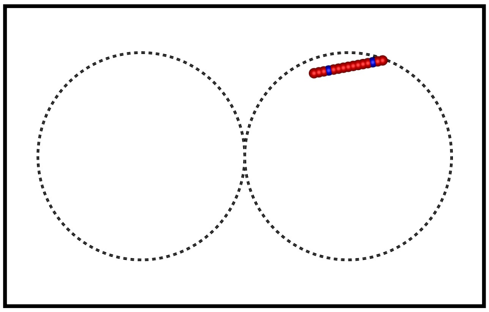

Rotation: 31.3^{o}

---+--------+--
Type 2, N – 15, theta: 190.5, yinter: 2.030, std: 0.000, mu: 0.790 > 0.990
zrotate: 31.3, scale: 0.290, stretch (r: 1.991,theta: 4.65), dxy: (-1.130,-0.190)

HRP-II-24p2/processed
Centroid: (1097.93,665.963)
---+--------+--
r average: 0.502393, std: 0.151989
a average: 4.65181, std: 15.6406
