# Supplementary material for: Möbius-strip-like columnar functional connections are revealed in somato-sensory receptive field centroids
Source: Front Neuroanat. 2014 Oct 31;8:119. doi: 10.3389/fnana.2014.00119 (PMC4215792; doi:10.3389/fnana.2014.00119)
Supplement: Supplementary file 1 [file SupplementaryMaterial.ZIP › Supplementary/All RF Centroid Plots and Model Best Fits/HRP-II-24p4_split1.pdf]

HRP-II-24p4 Split 1

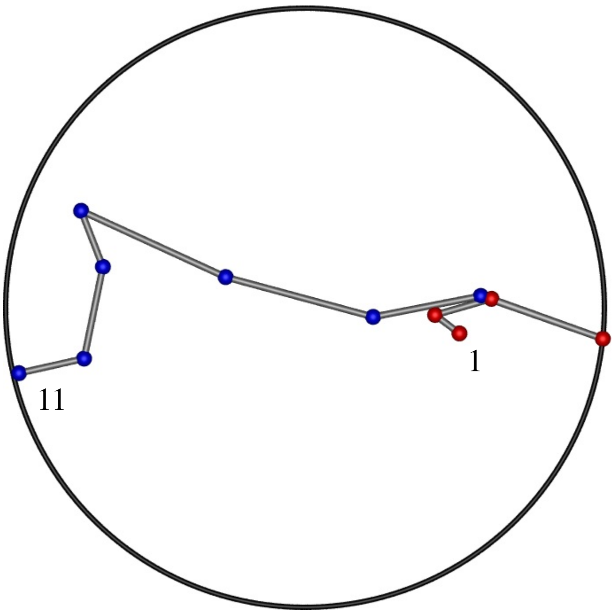

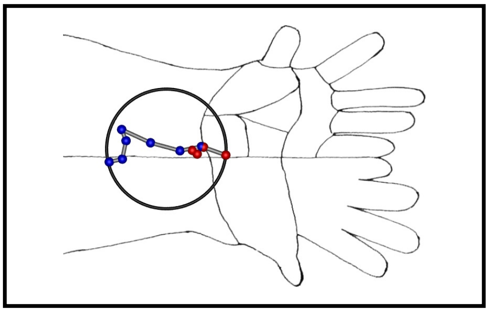

RF anisotropy: 4.885, -2.03^{o}

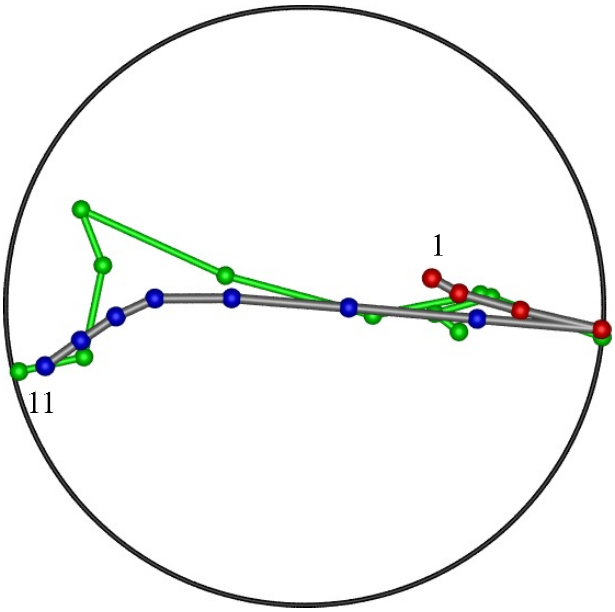

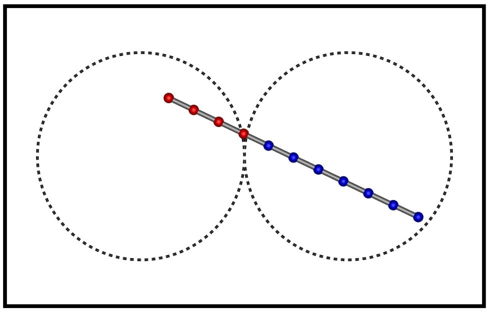

Rotation: 209.4^{o}

----+++++++

Type 2, N – 11, theta: 154.5, yinter: 0.640, std: 0.000, mu: 0.220 > 0.970
zrotate: 209.4, scale: 0.360, stretch (r: 4.885,theta: -2.03), dxy: (-2.000,0.340)

HRP-II-24p4/processed
Centroid: (920.265,610.892)
----+++++++
r average: 0.204695, std: 0.0440379
a average: -2.0282, std: 2.94324
