# Supplementary material for: Möbius-strip-like columnar functional connections are revealed in somato-sensory receptive field centroids
Source: Front Neuroanat. 2014 Oct 31;8:119. doi: 10.3389/fnana.2014.00119 (PMC4215792; doi:10.3389/fnana.2014.00119)
Supplement: Supplementary file 1 [file SupplementaryMaterial.ZIP › Supplementary/All RF Centroid Plots and Model Best Fits/HRP-II-24p4_split2.pdf]

HRP-II-24p4 Split 2

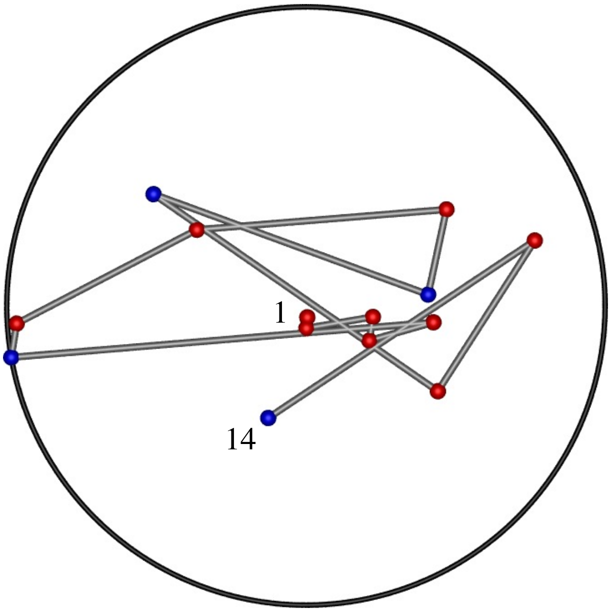

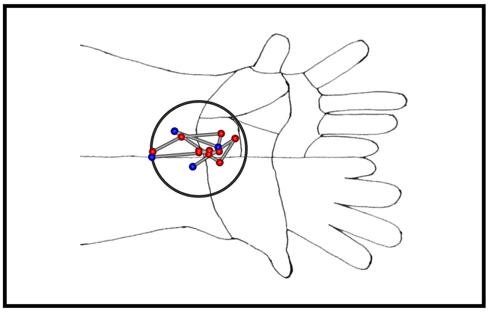

RF anisotropy: 3.464, -0.85^{o}

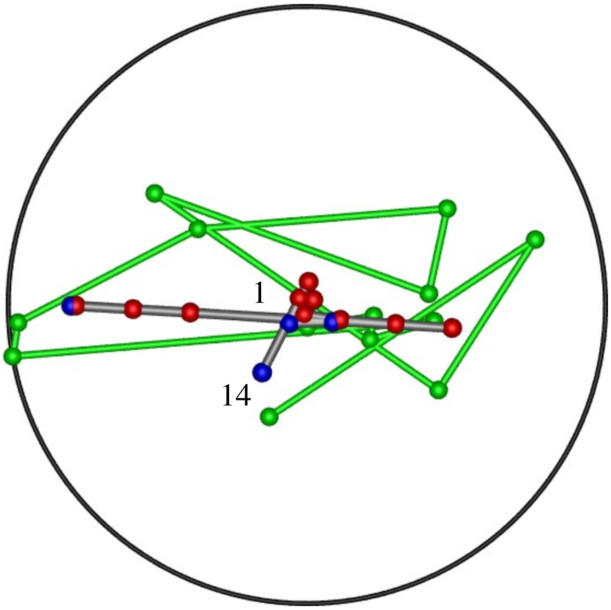

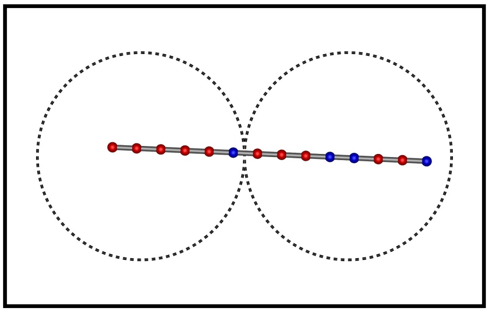

Rotation: 190.9^{o}

-----+---++--+
Type 2, N – 14, theta: 177.4, yinter: 0.090, std: 0.000, mu: 0.180 > 0.940
zrotate: 190.9, scale: 0.240, stretch (r: 3.464,theta: -0.85), dxy: (-0.170,-0.140)

HRP-II-24p4/processed
Centroid: (985.303,611.682)
-----+---++--+
r average: 0.288691, std: 0.0815612
a average: -0.852339, std: 2.15785
