# Supplementary material for: Möbius-strip-like columnar functional connections are revealed in somato-sensory receptive field centroids
Source: Front Neuroanat. 2014 Oct 31;8:119. doi: 10.3389/fnana.2014.00119 (PMC4215792; doi:10.3389/fnana.2014.00119)
Supplement: Supplementary file 1 [file SupplementaryMaterial.ZIP › Supplementary/All RF Centroid Plots and Model Best Fits/HRP-II-24p5.pdf]

HRP-II-24p5

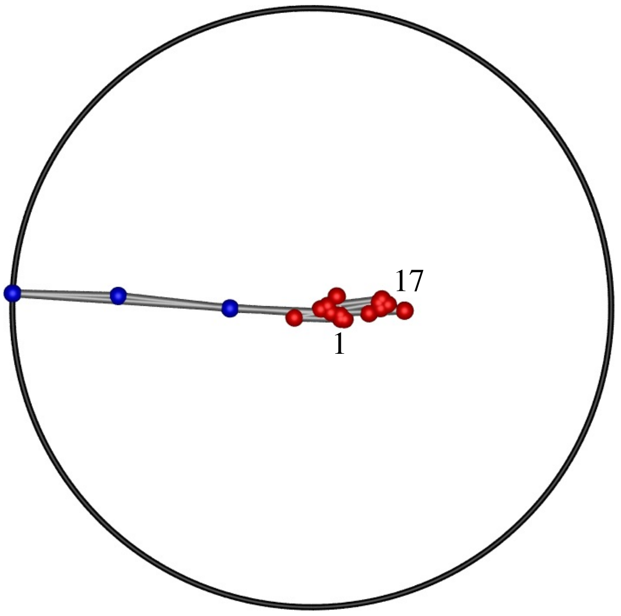

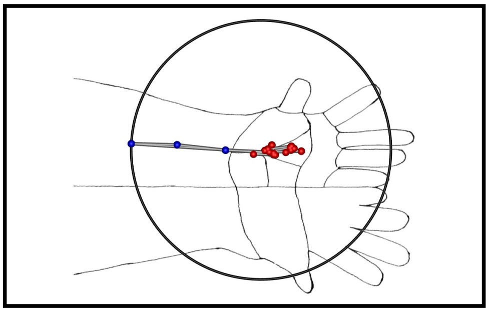

RF anisotropy: 2.213, 1.33^{o}

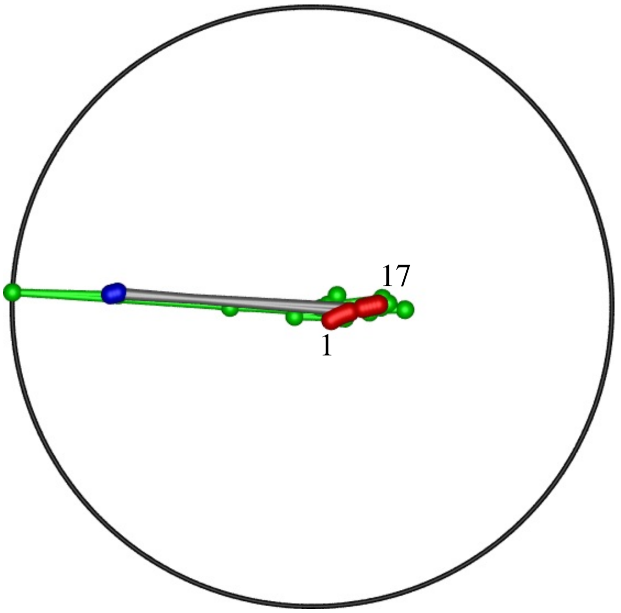

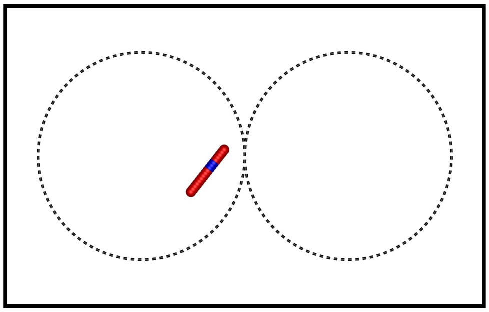

Rotation: 6.2^{o}

----------+++-----
Type 2, N – 17, theta: 51.7, yinter: 0.940, std: 0.000, mu: 0.370 > 0.600
zrotate: 6.2, scale: 0.270, stretch (r: 2.213,theta: 1.33), dxy: (-0.770,0.050)

HRP-II-24p5/processed
Centroid: (1049.25,675.083)
---------+++-----
r average: 0.451879, std: 0.14939
a average: 1.33353, std: 9.60172
