# Supplementary material for: Möbius-strip-like columnar functional connections are revealed in somato-sensory receptive field centroids
Source: Front Neuroanat. 2014 Oct 31;8:119. doi: 10.3389/fnana.2014.00119 (PMC4215792; doi:10.3389/fnana.2014.00119)
Supplement: Supplementary file 1 [file SupplementaryMaterial.ZIP › Supplementary/All RF Centroid Plots and Model Best Fits/HRP-II-24p7-9.pdf]

HRP-II-24p7-9

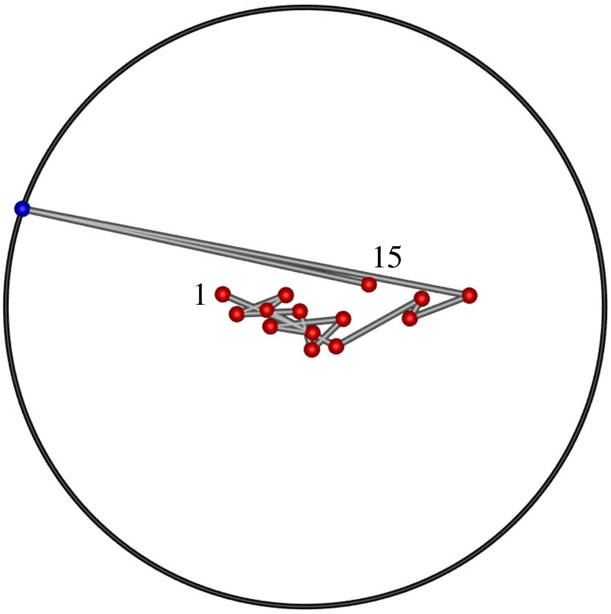

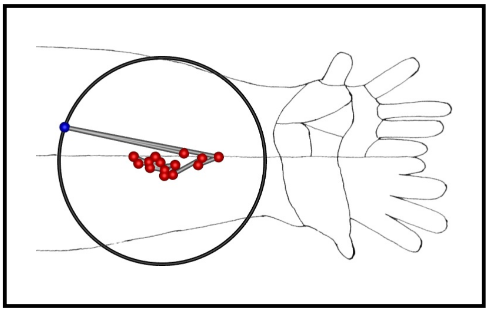

RF anisotropy: 4.605, -0.15^{o}

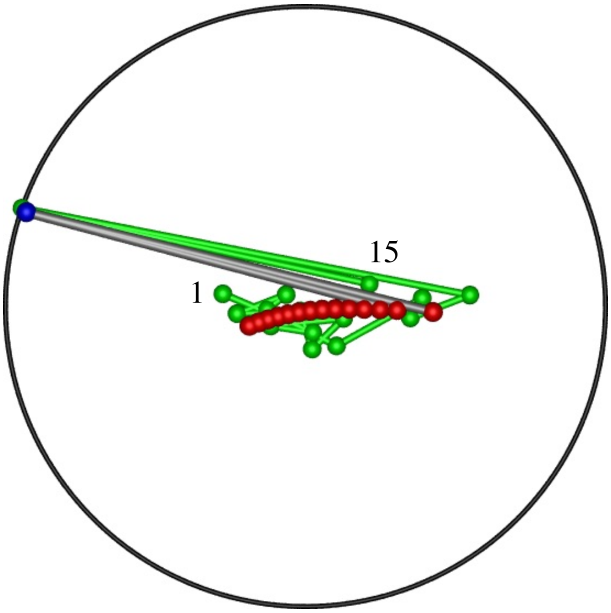

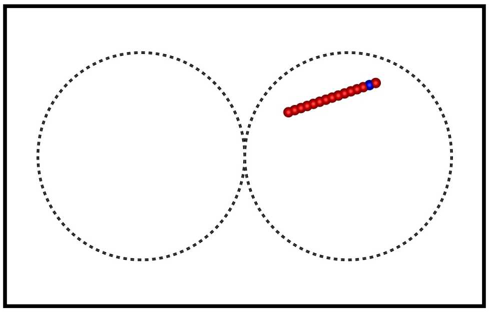

Rotation: 102.7^{o}

-------------+-
Type 2, N = 15, theta: 198.5, yinter: 0.850, std: 0.000, mu: 0.670 > 0.910
zrotate: 102.7, scale: 0.300, stretch (r: 4.605,theta: -0.15), dxy: (-0.850,0.450)

HRP-II-24p7-9/processed
Centroid: (734.798,586.984)
-------------+-
r average: 0.21717, std: 0.04828
a average: -0.154659, std: 2.05389
