# Supplementary material for: Möbius-strip-like columnar functional connections are revealed in somato-sensory receptive field centroids
Source: Front Neuroanat. 2014 Oct 31;8:119. doi: 10.3389/fnana.2014.00119 (PMC4215792; doi:10.3389/fnana.2014.00119)
Supplement: Supplementary file 1 [file SupplementaryMaterial.ZIP › Supplementary/All RF Centroid Plots and Model Best Fits/HRP-II-32p2-12_split1.pdf]

HRP-II-32p2-12 Split 1

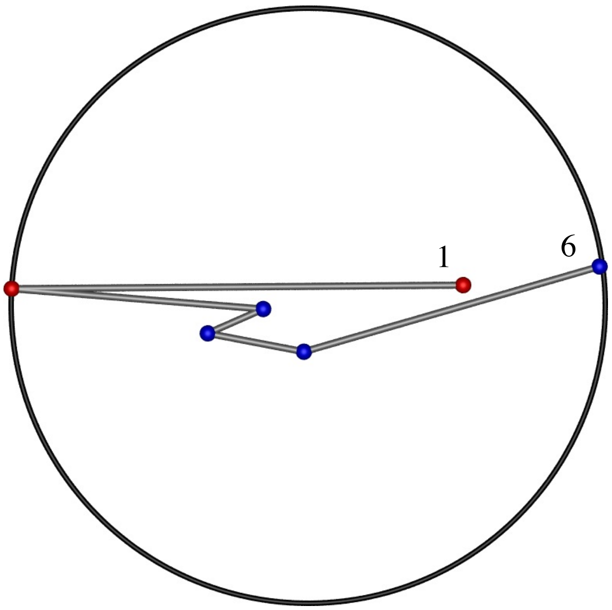


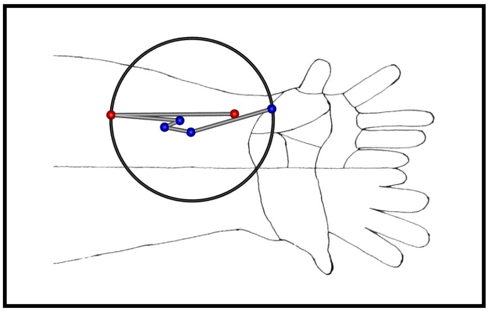

RF anisotropy: 3.697, 6.07$^{o}$

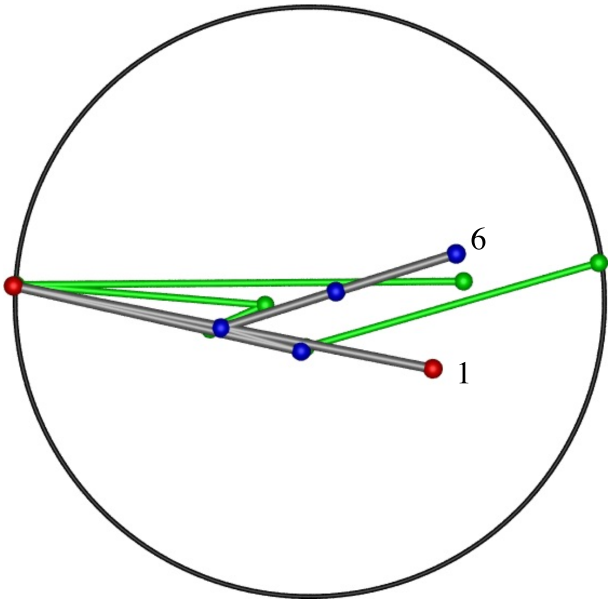


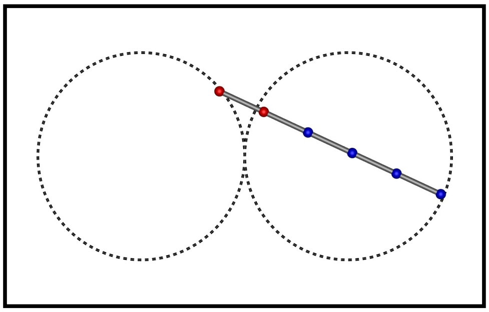

Rotation: 239.8$^{o}$

--++++
Type 2, N – 6, theta: 155.1, yinter: 1.540, std: 0.000, mu: 0.270 > 0.990
zrotate: 239.8, scale: 0.300, stretch (r: 3.697,theta: 6.07), dxy: (-1.070,-0.290)

HRP-II-32p2-12/processed
Centroid: (861.056,698.637)
--++++
r average: 0.270474, std: 0.112651
a average: 6.06969, std: 13.1789
