# Supplementary material for: Möbius-strip-like columnar functional connections are revealed in somato-sensory receptive field centroids
Source: Front Neuroanat. 2014 Oct 31;8:119. doi: 10.3389/fnana.2014.00119 (PMC4215792; doi:10.3389/fnana.2014.00119)
Supplement: Supplementary file 1 [file SupplementaryMaterial.ZIP › Supplementary/All RF Centroid Plots and Model Best Fits/HRP-II-32p2-12_split2.pdf]

HRP-II-32p2-12 Split 2

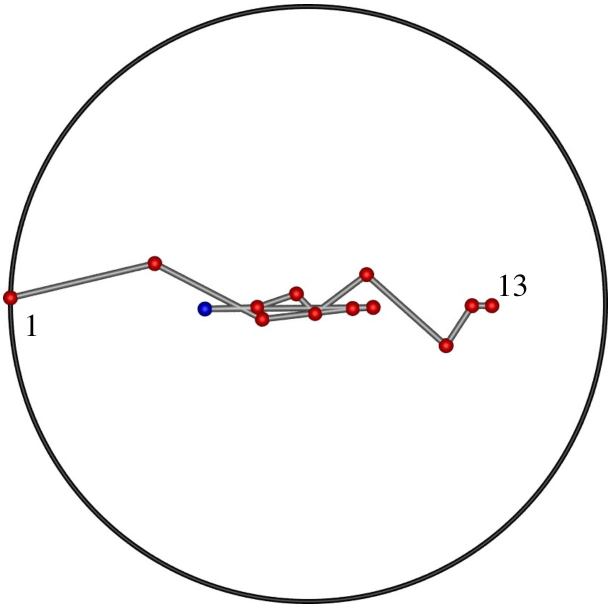

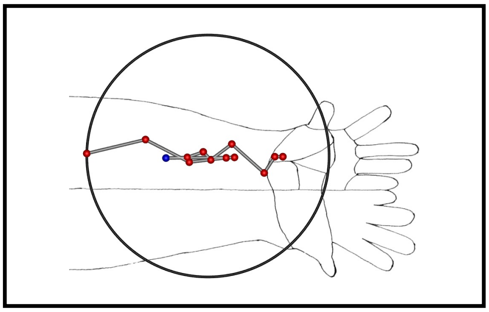

RF anisotropy: 3.503, -0.23^{o}

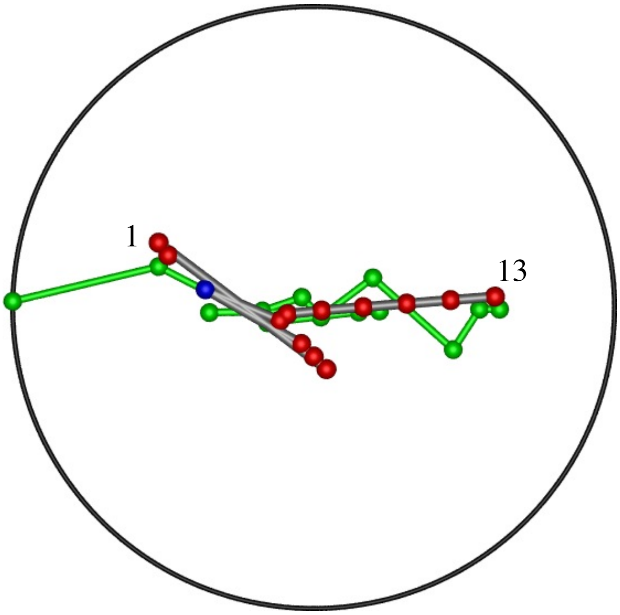

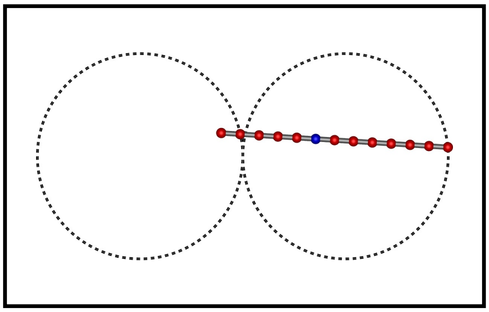

Rotation: 75.8^{o}

-----+--------

Type 2, N = 13, theta: 356.4, yinter: 0.640, std: 0.000, mu: 0.440 > 1.000
zrotate: 75.8, scale: 0.240, stretch (r: 3.503,theta: -0.23), dxy: (-0.660,-0.030)

HRP-II-32p2-12/processed
Centroid: (827.971,684.331)
-----+--------
r average: 0.285538, std: 0.120405
a average: -0.230367, std: 6.91044
