# Supplementary material for: Möbius-strip-like columnar functional connections are revealed in somato-sensory receptive field centroids
Source: Front Neuroanat. 2014 Oct 31;8:119. doi: 10.3389/fnana.2014.00119 (PMC4215792; doi:10.3389/fnana.2014.00119)
Supplement: Supplementary file 1 [file SupplementaryMaterial.ZIP › Supplementary/All RF Centroid Plots and Model Best Fits/HRP-II-34p2.pdf]

HRP-II-34p2

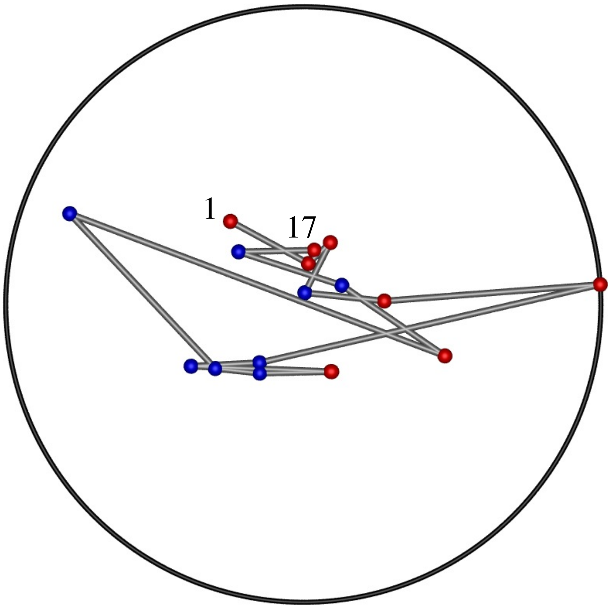

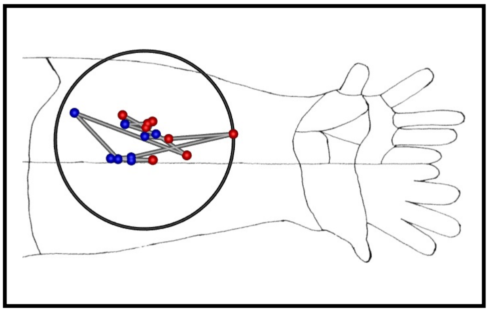

RF anisotropy: 3.331, 1.91^{o}

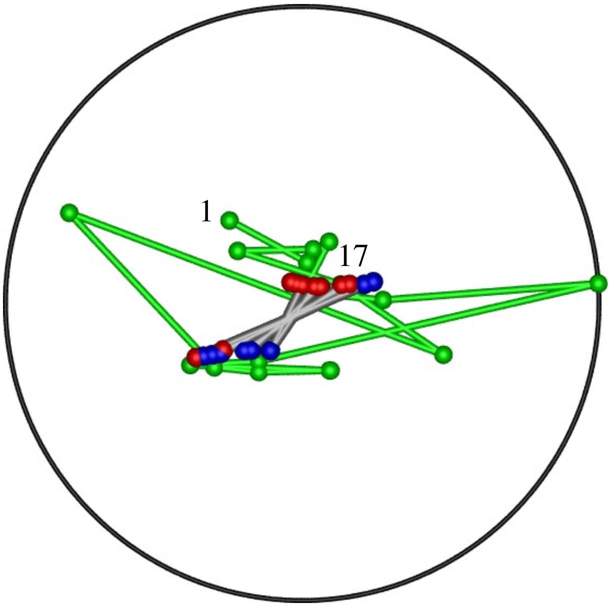

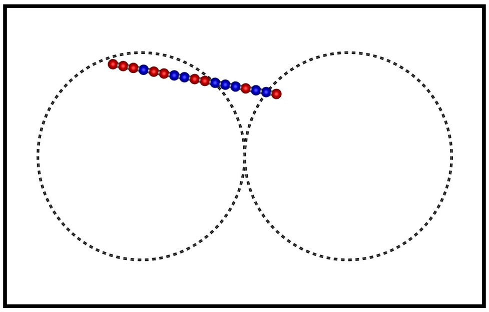

Rotation: 143.1^{o}

---+--++--+++-++-
Type 2, N = 17, theta: 349.7, yinter: 1.970, std: 0.000, mu: 0.040 > 0.510
zrotate: 143.1, scale: 0.130, stretch (r: 3.331,theta: 1.91), dxy: (-0.130,-0.150)

HRP-II-34p2/processed
Centroid: (640.634,650.76)
---+--++--+++-++-
r average: 0.30019, std: 0.136592
a average: 1.90928, std: 12.6945
