# Supplementary material for: Möbius-strip-like columnar functional connections are revealed in somato-sensory receptive field centroids
Source: Front Neuroanat. 2014 Oct 31;8:119. doi: 10.3389/fnana.2014.00119 (PMC4215792; doi:10.3389/fnana.2014.00119)
Supplement: Supplementary file 1 [file SupplementaryMaterial.ZIP › Supplementary/All RF Centroid Plots and Model Best Fits/HRP-II-34p3-1_split1.pdf]

HRP-II-34p3-1 Split 1

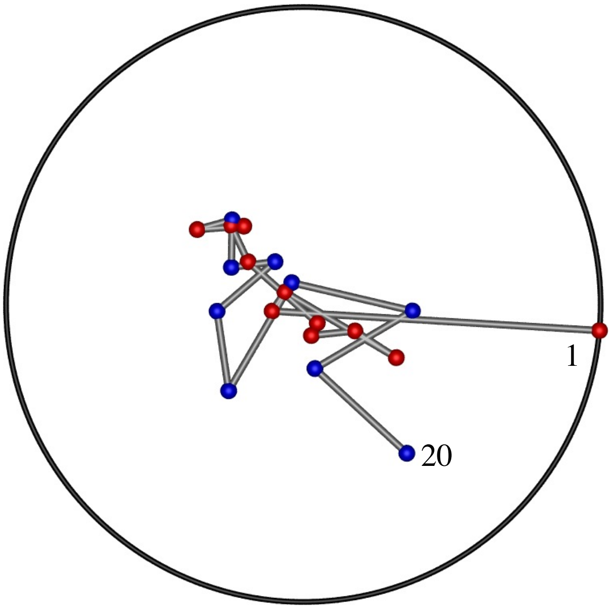

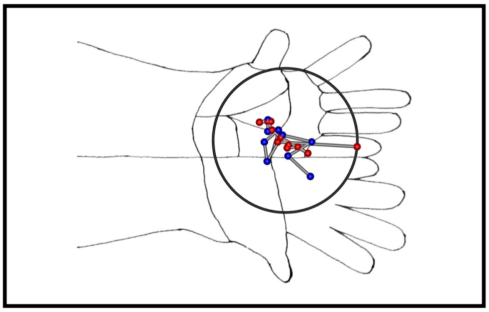

RF anisotropy: 2.785, 3.07^{o}

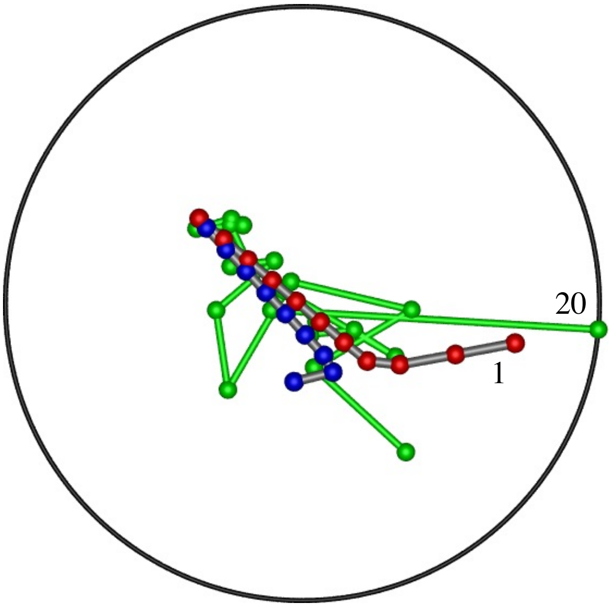

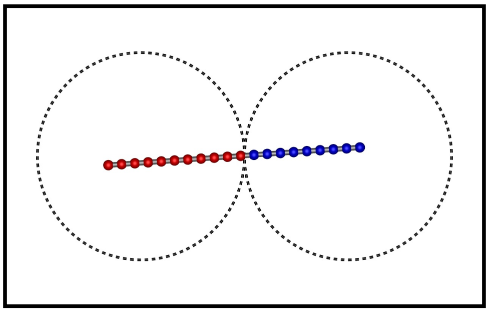

Rotation: 248.3^{o}

-----------+++++++++
Type 2, N – 20, theta: 4.0, yinter: 0.020, std: 0.000, mu: 0.170 > 0.780
zrotate: 248.3, scale: 0.620, stretch (r: 2.785,theta: 3.07), dxy: (0.650,-0.750)

HRP-II-34p3-1/processed
Centroid: (1147.73,626.449)
-----------+++++++++
r average: 0.359057, std: 0.196121
a average: 3.06544, std: 22.5286
