# Supplementary material for: Möbius-strip-like columnar functional connections are revealed in somato-sensory receptive field centroids
Source: Front Neuroanat. 2014 Oct 31;8:119. doi: 10.3389/fnana.2014.00119 (PMC4215792; doi:10.3389/fnana.2014.00119)
Supplement: Supplementary file 1 [file SupplementaryMaterial.ZIP › Supplementary/All RF Centroid Plots and Model Best Fits/HRP-II-34p3-1_split2.pdf]

HRP-II-34p3-1 Split 2

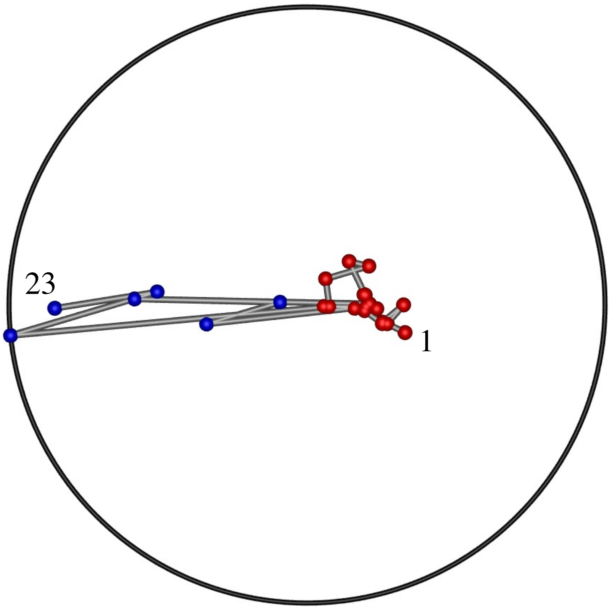

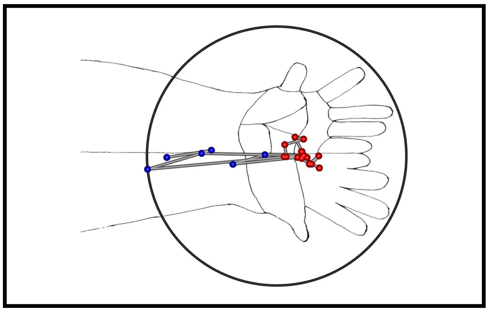

RF anisotropy: 2.976, -6.24°

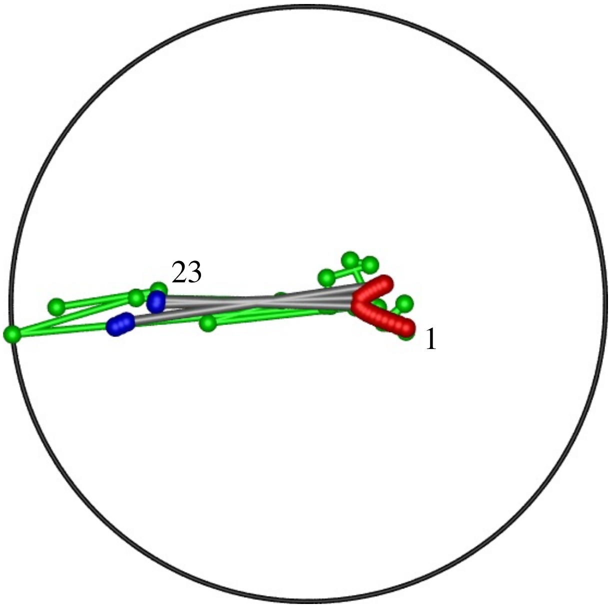

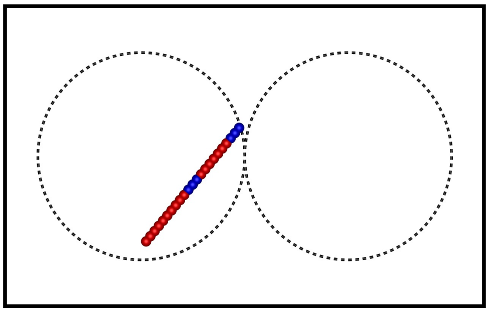

Rotation: 333.6°

----------+++-------+++

Type 2, N – 23, theta: 230.6, yinter: 1.020, std: 0.000, mu: 0.100 > 0.730
zrotate: 333.6, scale: 0.210, stretch (r: 2.976,theta: -6.24), dxy: (-0.510,0.020)

HRP-II-34p3-1/processed
Centroid: (1078.2,588.972)
----------+++-------+++
r average: 0.336025, std: 0.142517
a average: -6.24315, std: 13.4486
