# Supplementary material for: Möbius-strip-like columnar functional connections are revealed in somato-sensory receptive field centroids
Source: Front Neuroanat. 2014 Oct 31;8:119. doi: 10.3389/fnana.2014.00119 (PMC4215792; doi:10.3389/fnana.2014.00119)
Supplement: Supplementary file 1 [file SupplementaryMaterial.ZIP › Supplementary/All RF Centroid Plots and Model Best Fits/HRP-II-34p6-17.pdf]

HRP-II-34p6-17

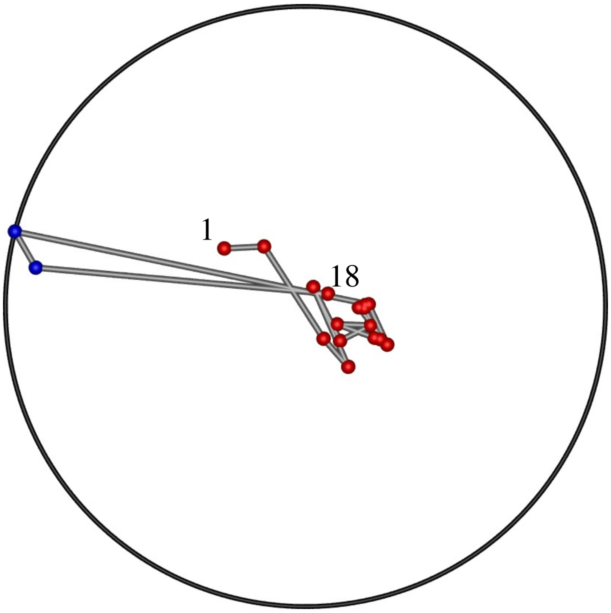

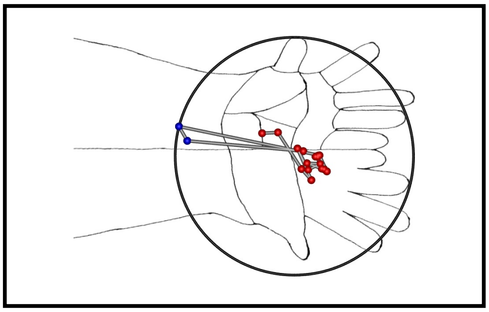

RF anisotropy: 2.233, -21.02^{o}

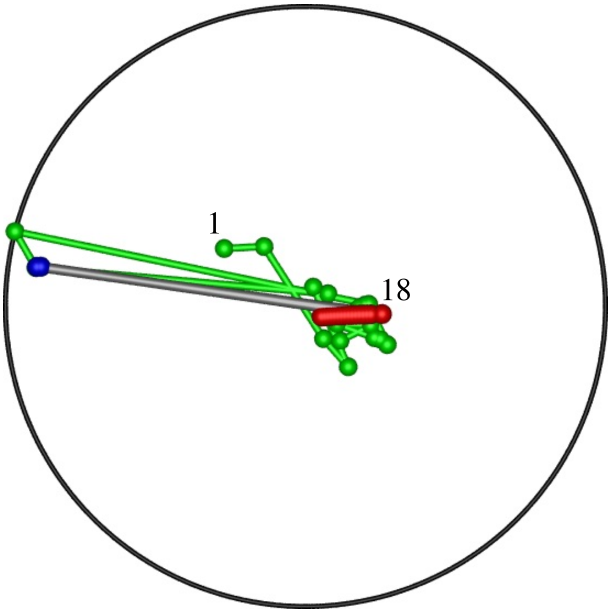

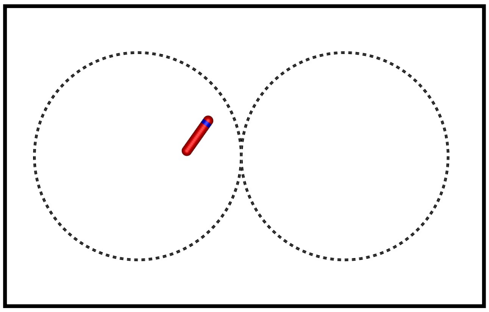

Rotation: 186.5^{o}

---------------++-
Type 2, N – 18, theta: 54.6, yinter: 2.380, std: 0.000, mu: 0.670 > 0.860
zrotate: 186.5, scale: 0.380, stretch (r: 2.233,theta: -21.02), dxy: (-0.980,0.160)

HRP-II-34p6-17/processed
Centroid: (1129.34,582.268)
---------------++-
r average: 0.447903, std: 0.202445
a average: -21.0227, std: 13.8947
