# Supplementary material for: Möbius-strip-like columnar functional connections are revealed in somato-sensory receptive field centroids
Source: Front Neuroanat. 2014 Oct 31;8:119. doi: 10.3389/fnana.2014.00119 (PMC4215792; doi:10.3389/fnana.2014.00119)
Supplement: Supplementary file 1 [file SupplementaryMaterial.ZIP › Supplementary/All RF Centroid Plots and Model Best Fits/HRP-II-34p7_split1.pdf]

HRP-II-34p7 Split 1

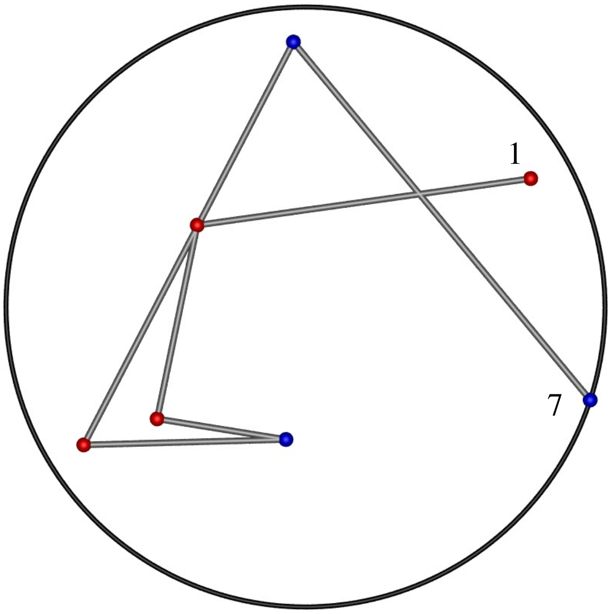

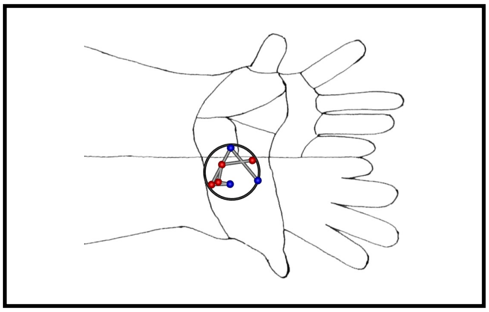

RF anisotropy: 2.887, 1.82^{o}

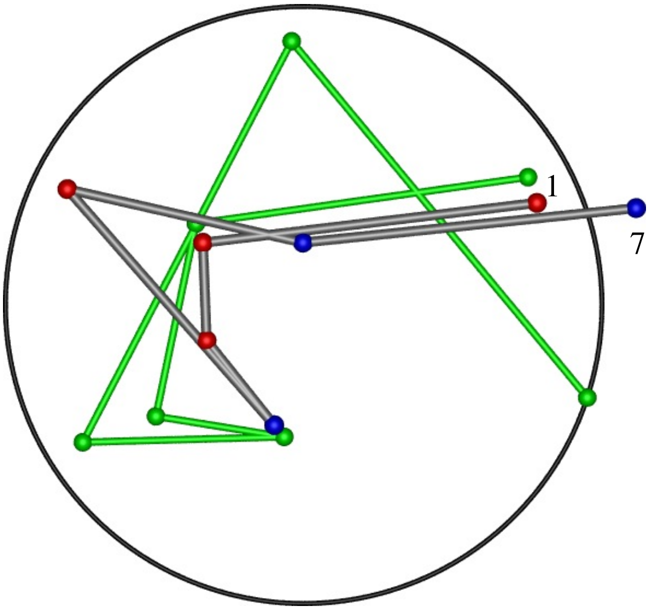

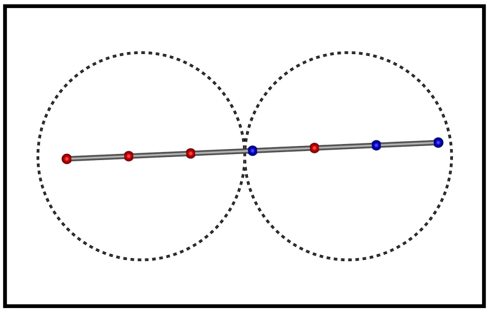

Rotation: 255.3^{o}

---+-++
Type 2, N = 7, theta: 182.5, yinter: 0.150, std: 0.000, mu: 0.070 > 0.970
zrotate: 255.3, scale: 0.670, stretch (r: 2.887,theta: 1.82), dxy: (-1.700,0.550)

HRP-II-34p7/processed
Centroid: (1052.63,568.234)
---+-++
r average: 0.346402, std: 0.0804874
a average: 1.81892, std: 8.84046
