# Supplementary material for: Möbius-strip-like columnar functional connections are revealed in somato-sensory receptive field centroids
Source: Front Neuroanat. 2014 Oct 31;8:119. doi: 10.3389/fnana.2014.00119 (PMC4215792; doi:10.3389/fnana.2014.00119)
Supplement: Supplementary file 1 [file SupplementaryMaterial.ZIP › Supplementary/All RF Centroid Plots and Model Best Fits/HRP-II-34p7_split2.pdf]

HRP-II-34p7 Split 2

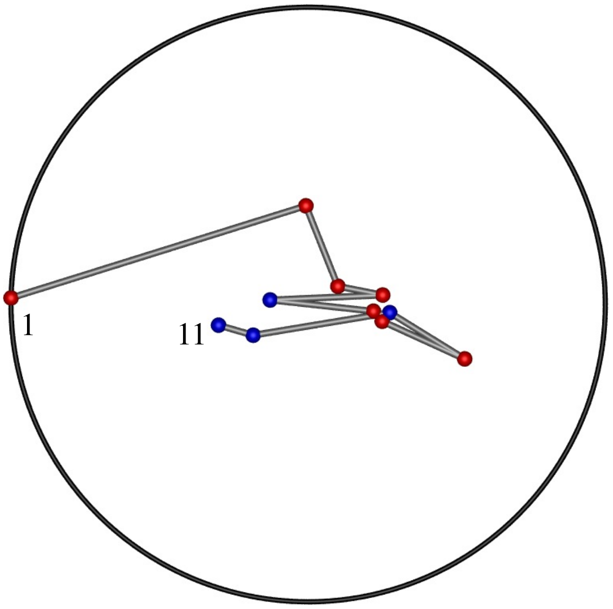

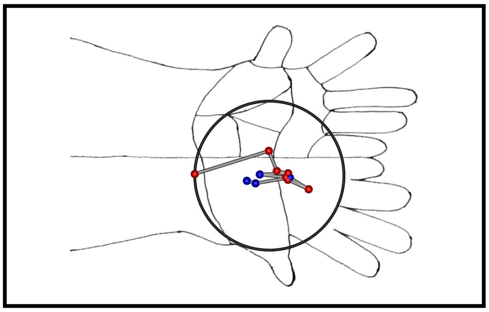

RF anisotropy: 3.037, -1.73°

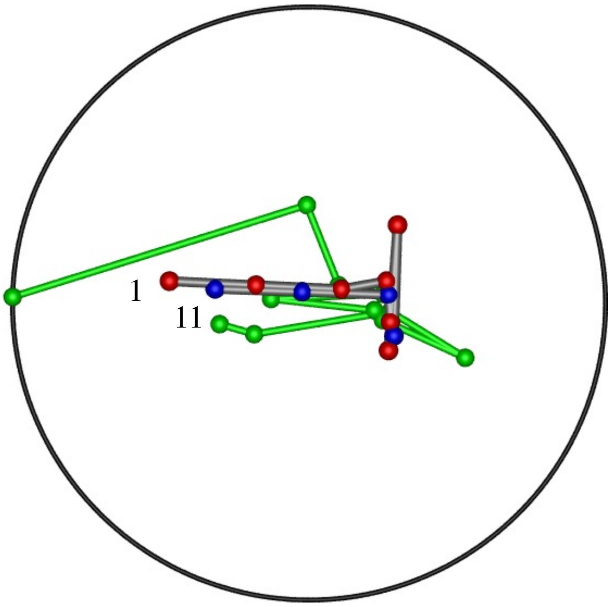

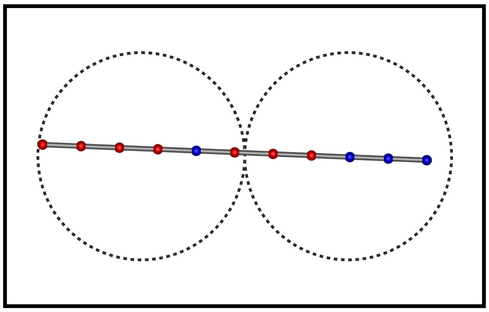

Rotation: 93.6°

----+---+++
Type 2, N – 11, theta: 357.7, yinter: 0.100, std: 0.000, mu: 0.010 > 0.940
zrotate: 93.6, scale: 0.260, stretch (r: 3.037,theta: -1.73), dxy: (0.850,0.090)

HRP-II-34p7/processed
Centroid: (1115.37,564.138)
----+---+++
r average: 0.32929, std: 0.0741983
a average: -1.72789, std: 12.4793
