# Supplementary material for: Möbius-strip-like columnar functional connections are revealed in somato-sensory receptive field centroids
Source: Front Neuroanat. 2014 Oct 31;8:119. doi: 10.3389/fnana.2014.00119 (PMC4215792; doi:10.3389/fnana.2014.00119)
Supplement: Supplementary file 1 [file SupplementaryMaterial.ZIP › Supplementary/All RF Centroid Plots and Model Best Fits/HRP-II-35p1_split1.pdf]

HRP-II-35p1 Split 1

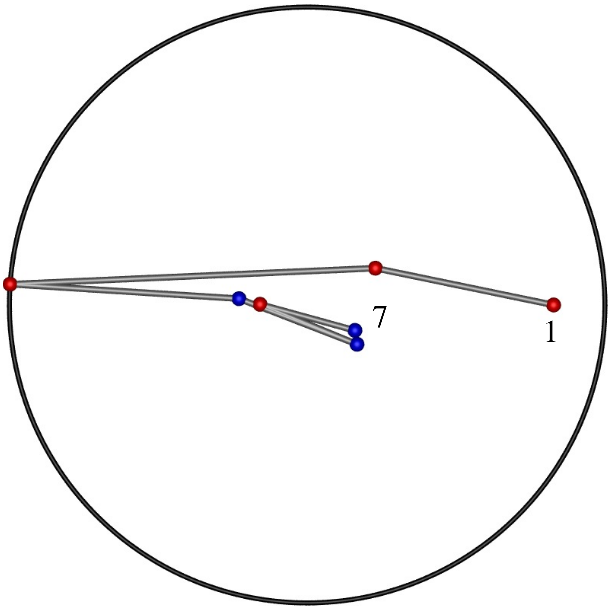

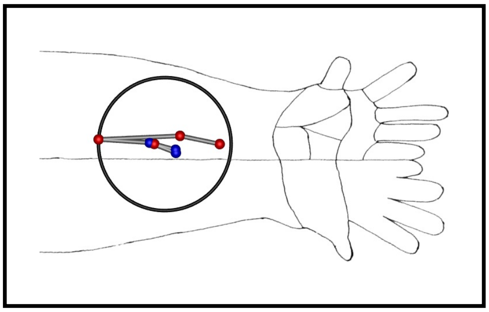

RF anisotropy: 6.609, -2.43^{o}

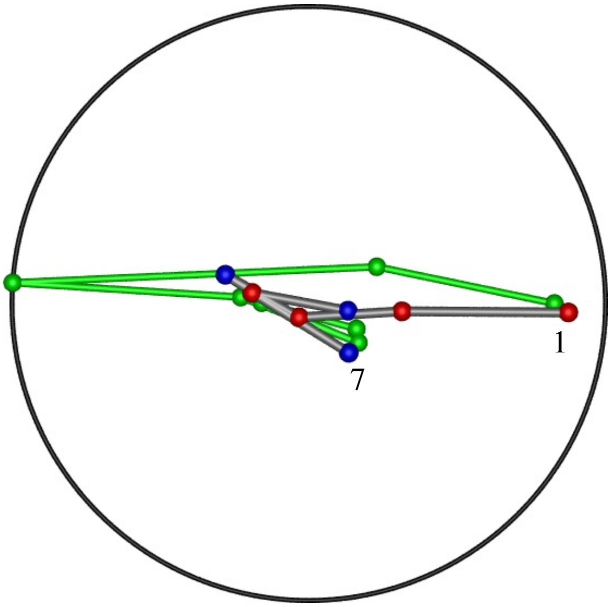

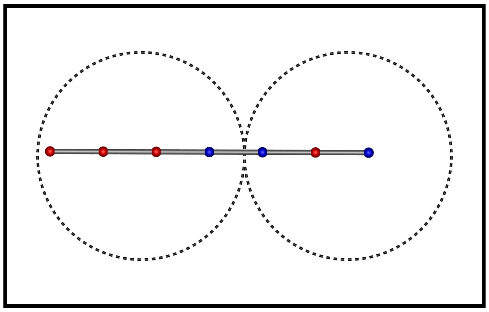

Rotation: 259.1^{o}

---++-+
Type 2, N = 7, theta: 359.8, yinter: 0.110, std: 0.000, mu: 0.030 > 0.800
zrotate: 259.1, scale: 0.170, stretch (r: 6.609,theta: -2.43), dxy: (-0.280,-0.060)

HRP-II-35p1/processed
Centroid: (738.332,631.532)
---++-+
r average: 0.151298, std: 0.0243638
a average: -2.43129, std: 2.17519
