# Supplementary material for: Möbius-strip-like columnar functional connections are revealed in somato-sensory receptive field centroids
Source: Front Neuroanat. 2014 Oct 31;8:119. doi: 10.3389/fnana.2014.00119 (PMC4215792; doi:10.3389/fnana.2014.00119)
Supplement: Supplementary file 1 [file SupplementaryMaterial.ZIP › Supplementary/All RF Centroid Plots and Model Best Fits/HRP-II-35p1_split2.pdf]

HRP-II-35p1 Split 2

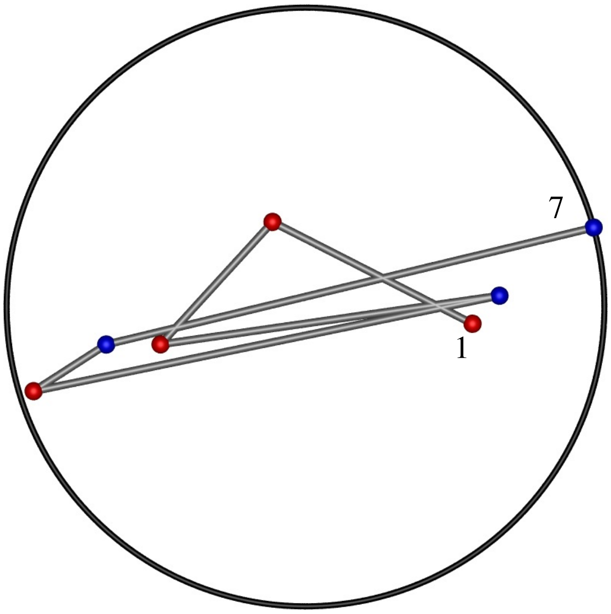

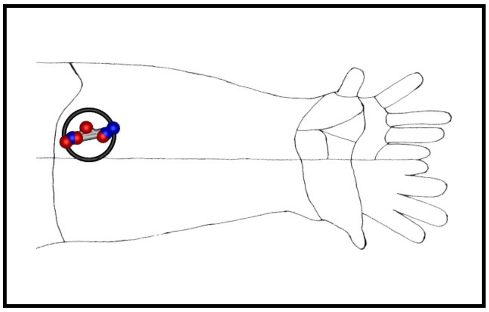

RF anisotropy: 5.172, -1.23°

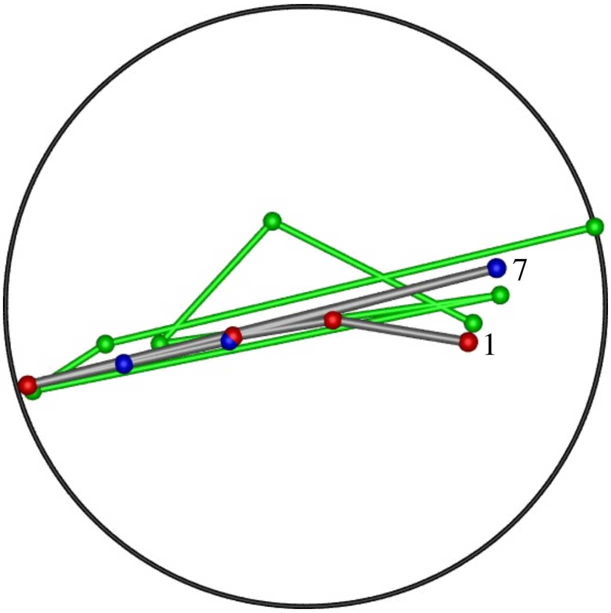

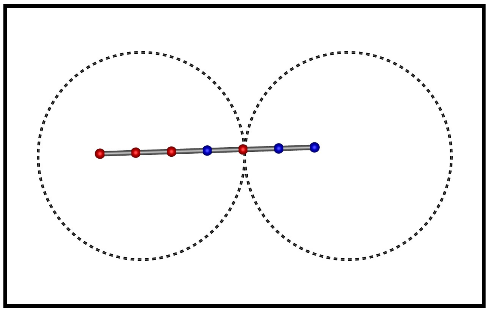

Rotation: 308.6°

---+-++
Type 2, N – 7, theta: 1.7, yinter: 0.190, std: 0.000, mu: 0.150 > 0.670
zrotate: 308.6, scale: 0.320, stretch (r: 5.172,theta: -1.23), dxy: (0.080,-0.080)

HRP-II-35p1/processed
Centroid: (464.493,658.249)
---+-++
r average: 0.193352, std: 0.0622793
a average: -1.23199, std: 4.05499
