# Supplementary material for: Möbius-strip-like columnar functional connections are revealed in somato-sensory receptive field centroids
Source: Front Neuroanat. 2014 Oct 31;8:119. doi: 10.3389/fnana.2014.00119 (PMC4215792; doi:10.3389/fnana.2014.00119)
Supplement: Supplementary file 1 [file SupplementaryMaterial.ZIP › Supplementary/All RF Centroid Plots and Model Best Fits/HRP-II-36p1.pdf]

HRP-II-36p1

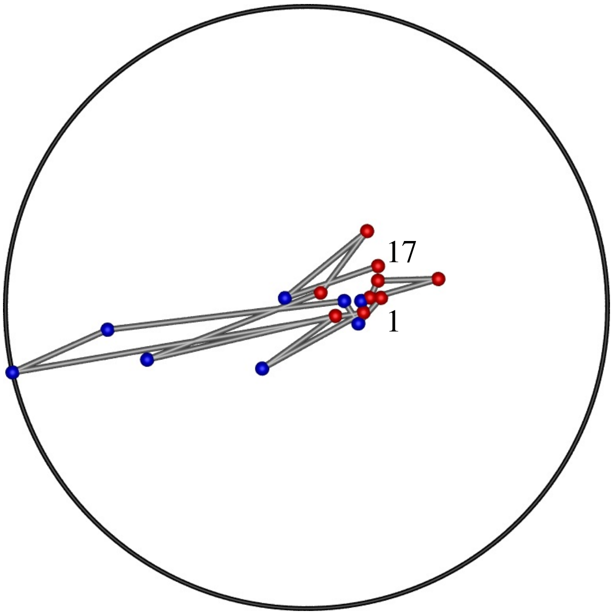

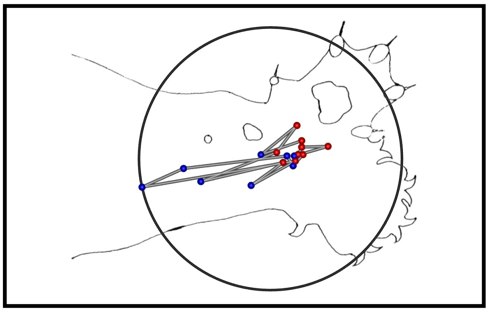

RF anisotropy: 3.226, 8.96^{o}

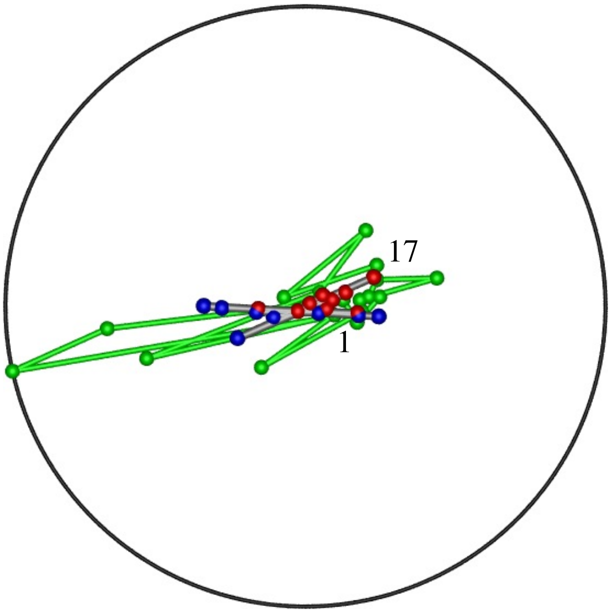

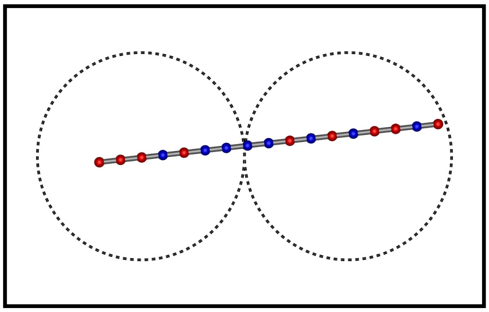

Rotation: 209.2^{o}

---+-++++-+-+--+-
Type 2, N – 17, theta: 186.4, yinter: 0.300, std: 0.000, mu: 0.150 > 0.980
zrotate: 209.2, scale: 0.120, stretch (r: 3.226,theta: 8.96), dxy: (-0.070,-0.050)

HRP-II-36p1/processed/
Centroid: (1011.71,661.937)
---+-++++-+-+--+-
r average: 0.310245, std: 0.171419
a average: 8.96179, std: 12.3041
