# Supplementary material for: Möbius-strip-like columnar functional connections are revealed in somato-sensory receptive field centroids
Source: Front Neuroanat. 2014 Oct 31;8:119. doi: 10.3389/fnana.2014.00119 (PMC4215792; doi:10.3389/fnana.2014.00119)
Supplement: Supplementary file 1 [file SupplementaryMaterial.ZIP › Supplementary/All RF Centroid Plots and Model Best Fits/HRP-II-36p2.pdf]

HRP-II-36p2

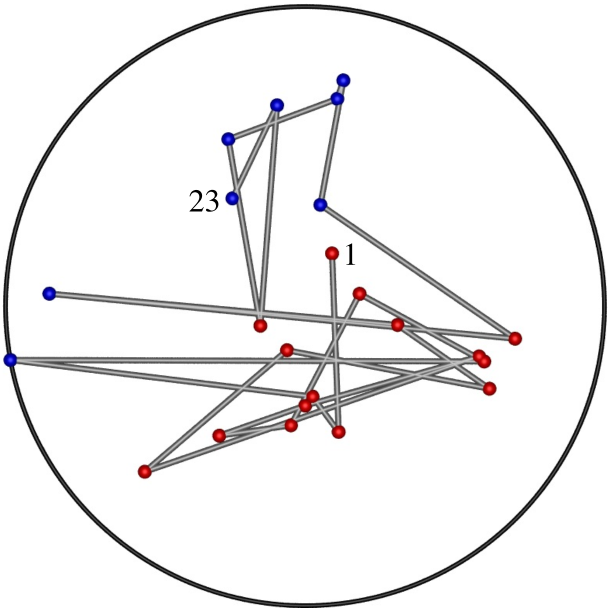

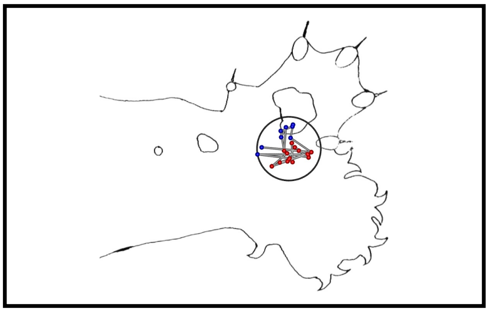

RF anisotropy: 1.612, -7.37°

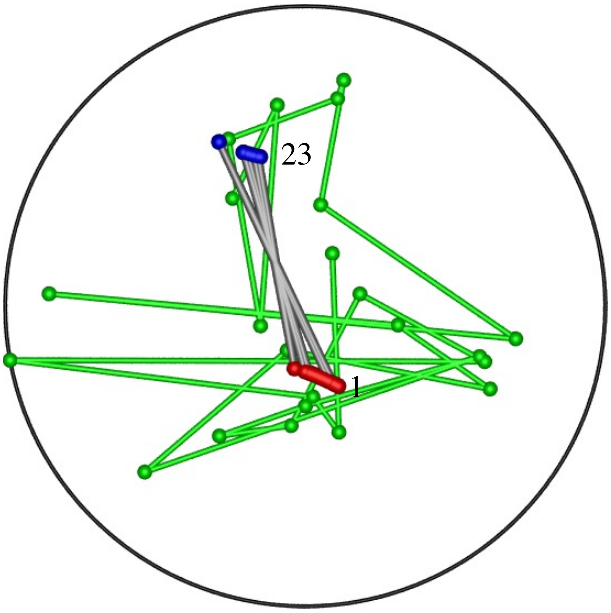

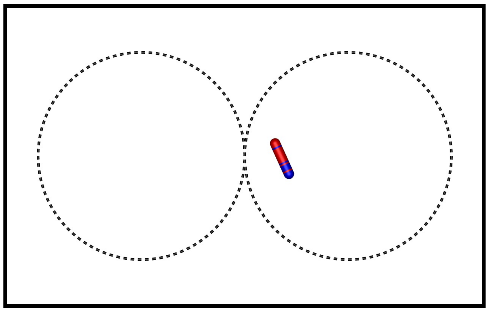

Rotation: 79.2°

---+----------+-++++-++
Type 2, N – 23, theta: 114.5, yinter: 2.300, std: 0.000, mu: 0.250 > 0.450
zrotate: 79.2, scale: 0.580, stretch (r: 1.612,theta: -7.37), dxy: (-0.300,0.440)

HRP-II-36p2/processed
Centroid: (1123.91,705.346)
---+----------+-++++-++
r average: 0.609179, std: 0.169677
a average: -7.37248, std: 45.9395
