# Supplementary material for: Möbius-strip-like columnar functional connections are revealed in somato-sensory receptive field centroids
Source: Front Neuroanat. 2014 Oct 31;8:119. doi: 10.3389/fnana.2014.00119 (PMC4215792; doi:10.3389/fnana.2014.00119)
Supplement: Supplementary file 1 [file SupplementaryMaterial.ZIP › Supplementary/All RF Centroid Plots and Model Best Fits/HRP-II-36_split1.pdf]

HRP-II-36 Split 1

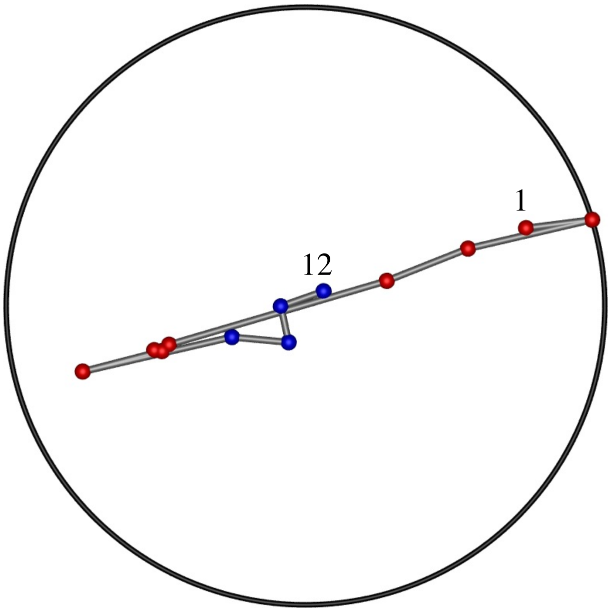

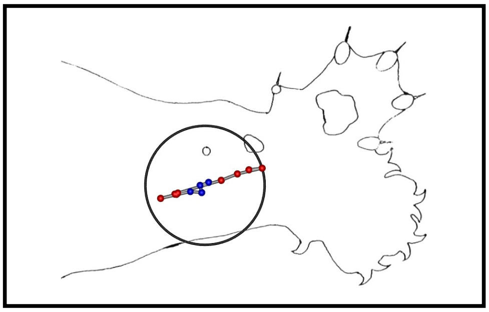

RF anisotropy: 3.25, 8.13^{o}

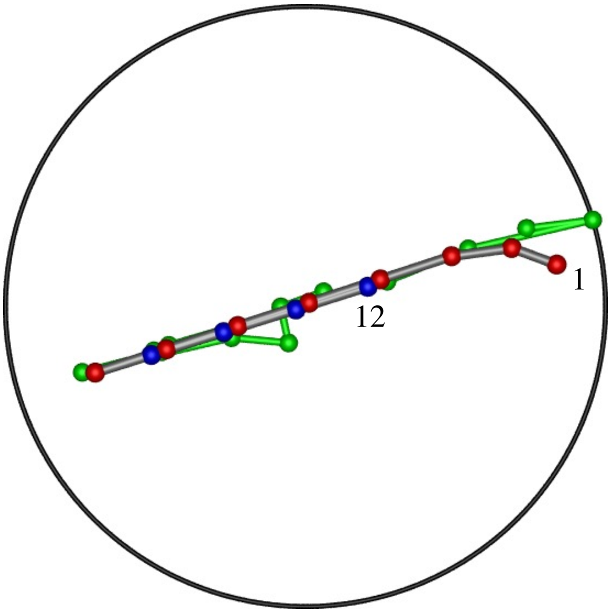

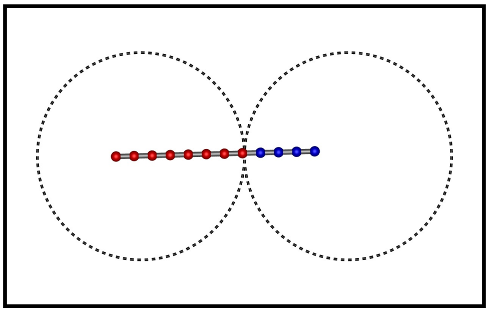

Rotation: 322.9^{o}

--------++++
Type 2, N – 12, theta: 1.5, yinter: 0.090, std: 0.000, mu: 0.190 > 0.670
zrotate: 322.9, scale: 0.500, stretch (r: 3.250,theta: 8.13), dxy: (1.900,0.650)

HRP-II-36/processed
Centroid: (878.314,633.836)
--------++++
r average: 0.307549, std: 0.13298
a average: 8.13083, std: 5.68991
