# Supplementary material for: Möbius-strip-like columnar functional connections are revealed in somato-sensory receptive field centroids
Source: Front Neuroanat. 2014 Oct 31;8:119. doi: 10.3389/fnana.2014.00119 (PMC4215792; doi:10.3389/fnana.2014.00119)
Supplement: Supplementary file 1 [file SupplementaryMaterial.ZIP › Supplementary/All RF Centroid Plots and Model Best Fits/HRP-II-36_split2.pdf]

HRP-II-36 Split 2

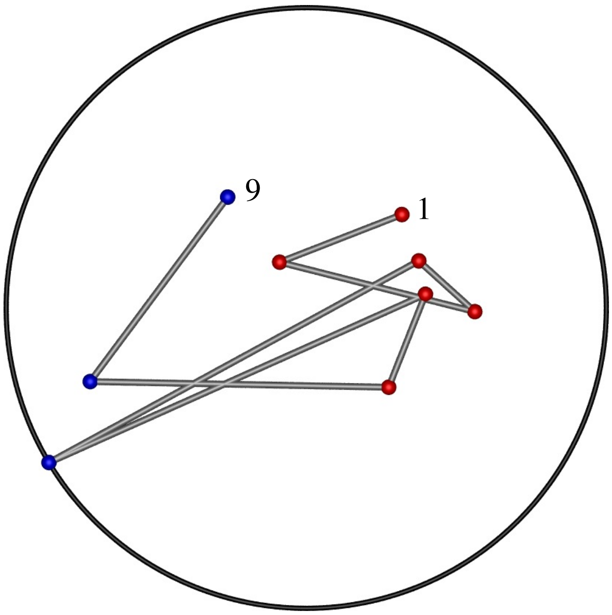

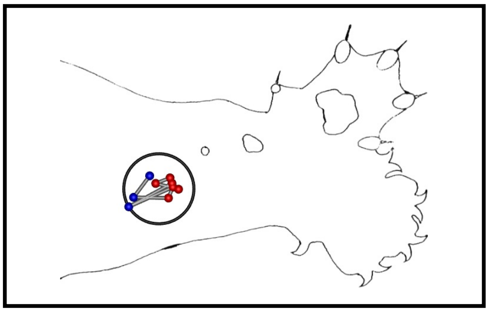

RF anisotropy: 3.34, 2.59°

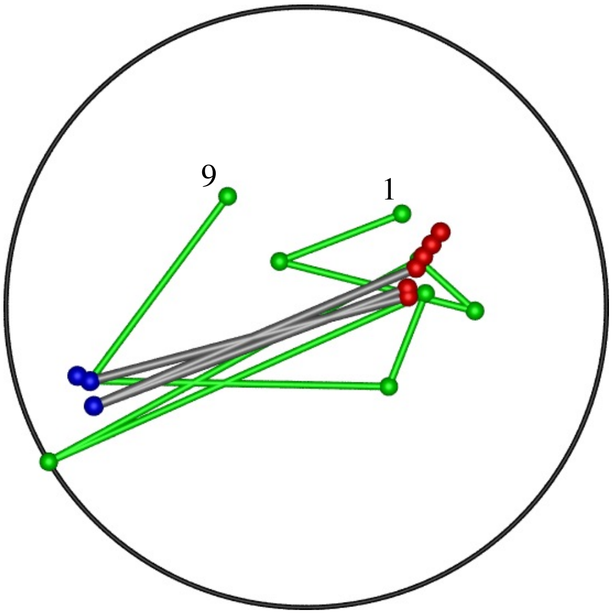

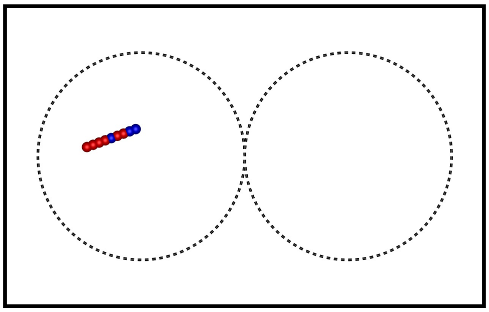

Rotation: 202.3°

----+--++
Type 2, N – 9, theta: 200.2, yinter: 1.950, std: 0.000, mu: 0.160 > 0.320
zrotate: 202.3, scale: 0.730, stretch (r: 3.340,theta: 2.59), dxy: (-0.540,-0.350)

HRP-II-36/processed
Centroid: (794.989,628.05)
----+--++
r average: 0.298412, std: 0.0963542
a average: 3.07572, std: 5.47806
