# Supplementary material for: Möbius-strip-like columnar functional connections are revealed in somato-sensory receptive field centroids
Source: Front Neuroanat. 2014 Oct 31;8:119. doi: 10.3389/fnana.2014.00119 (PMC4215792; doi:10.3389/fnana.2014.00119)
Supplement: Supplementary file 1 [file SupplementaryMaterial.ZIP › Supplementary/All RF Centroid Plots and Model Best Fits/index.html]

Supplementary material


|  |
| --- |
| Supplementary material |
| **RF centroid plots and results of model fitting for all datasets** |  |  | | --- | --- | | **Cats: Type I** CAT8615-p1  CAT8615-p2  HRP-II-36p2  HRP-II-36 Split 2  CAT\_874\_p\_2 | **Cats: Type II** HRP-II-36 Split 1  HRP-II-36p1 | |  |  | | **Monkeys: Type I** HRP-II-24p2  HRP-II-34p6-17  HRP-II-34p3-1 Split 2  HRP-II-24p5  HRP-II-24p7-9 | **Monkeys: Type II** HRP-II-24p4 Split 1  HRP-II-34p3-1 Split 1  HRP-II-32p2-12 Split 1  HRP-II-32p2-12 Split 2  HRP-II-34p2  HRP-II-34p7 Split 1  HRP-II-34p7 Split 2  HRP-II-35p1 Split 1  HRP-II-35p1 Split 2  HRP-II-24p4 Split 2 | |
| **Animations of vertical, horizontal, and oblique electrode penetrations.** Figure3a  Figure3b  Figure3c |
| **RF Nest analysis for all datasets** |  |  | | --- | --- | | **Cats: Type I** CAT8615-p1  CAT8615-p2  HRP-II-36p2  HRP-II-36 Split 2  CAT\_874\_p\_2 | **Cats: Type II** HRP-II-36 Split 1  HRP-II-36p1 | |  |  | | **Monkeys: Type I** HRP-II-24p2  HRP-II-34p6-17  HRP-II-34p3-1 Split 2  HRP-II-24p5  HRP-II-24p7-9 | **Monkeys: Type II** HRP-II-24p4 Split 1  HRP-II-34p3-1 Split 1  HRP-II-32p2-12 Split 1  HRP-II-32p2-12 Split 2  HRP-II-34p2  HRP-II-34p7 Split 1  HRP-II-34p7 Split 2  HRP-II-35p1 Split 1  HRP-II-35p1 Split 2  HRP-II-24p4 Split 2 | |
| **Statistics** **Noise-to-signal ratios (goodness of fit) summary** |
