# Supplementary material for: Möbius-strip-like columnar functional connections are revealed in somato-sensory receptive field centroids
Source: Front Neuroanat. 2014 Oct 31;8:119. doi: 10.3389/fnana.2014.00119 (PMC4215792; doi:10.3389/fnana.2014.00119)
Supplement: Supplementary file 1 [file SupplementaryMaterial.ZIP › Supplementary/Statistics/statistics.pdf]

Dataset name	Number of neurons	Sum squared distance from origin	Sum squared distance between experimental and simulation	RMS Noise/signal ratio
CAT8615-p1	21	40.07	44.72	1.06
CAT8615-p2	11	16.40	4.30	0.51
CAT_874_p_2	13	19.23	9.43	0.70
HRP-II-24p2	15	12.47	4.85	0.62
HRP-II-24p4_split1	11	45.05	2.48	0.23
HRP-II-24p4_split2	14	39.56	23.00	0.76
HRP-II-24p5	17	17.15	3.59	0.46
HRP-II-24p7-9	15	16.86	3.89	0.48
HRP-II-32p2-12_split1	6	21.84	3.79	0.42
HRP-II-32p2-12_split2	13	22.54	5.12	0.48
HRP-II-34p2	17	26.61	25.92	0.99
HRP-II-34p3-1_split1	20	24.51	11.39	0.68
HRP-II-34p3-1_split2	23	29.40	5.01	0.41
HRP-II-34p6-17	18	23.01	3.86	0.41
HRP-II-34p7_split1	7	36.71	15.44	0.65
HRP-II-34p7_split2	11	16.62	7.35	0.67
HRP-II-35p1_split1	7	17.18	8.95	0.72
HRP-II-35p1_split2	7	31.04	16.72	0.73
HRP-II-36_split1	12	31.34	5.26	0.41
HRP-II-36_split2	9	25.19	9.95	0.63
HRP-II-36p1	17	22.56	13.31	0.77
HRP-II-36p2	23	65.70	41.72	0.80
**Average**		27.32	12.28	0.62
