# Supplementary material for: Möbius-strip-like columnar functional connections are revealed in somato-sensory receptive field centroids
Source: Front Neuroanat. 2014 Oct 31;8:119. doi: 10.3389/fnana.2014.00119 (PMC4215792; doi:10.3389/fnana.2014.00119)
Supplement: Supplementary file 1 [file SupplementaryMaterial.ZIP › Supplementary/Variations of Best Fit/Variations of Best Fit.PDF]

COMPARISON OF RMS ERROR OBTAINED WITH CORRECT DESIGNATION OF POINTS (TOP ROW), WITH ALTERNATIVE SEQUENCES.

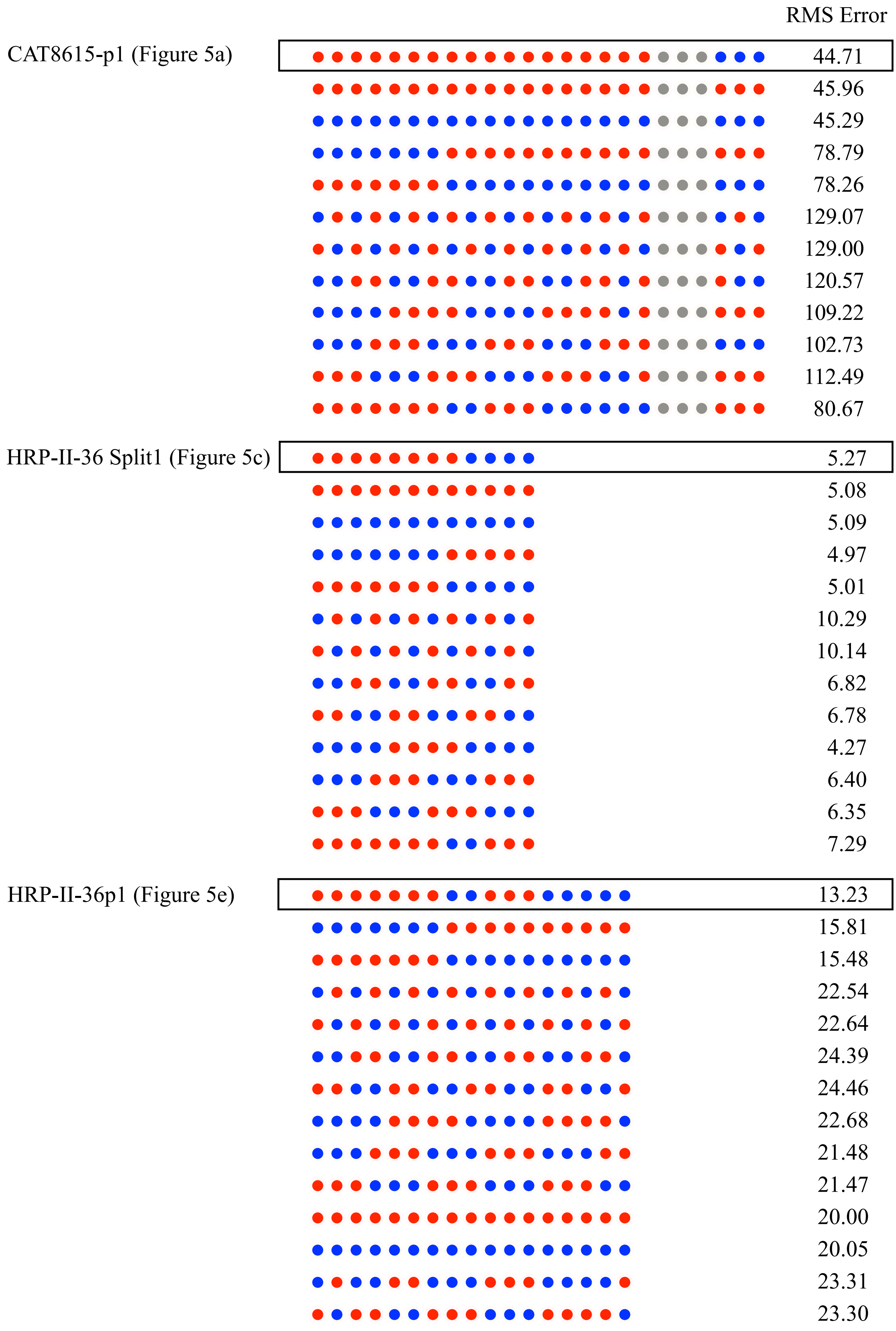

Notes

- Best fit is generally obtained for those red/blue designations of RF centroid positions that follow the patterns predicted in Figures 3a,b,c.
- Alternative designations close to those predicted have better fit than designations far from the prediction.
- Equivalent fits are obtained for inverses, where centroids are labelled in reverse colours.
- HRP-II-36 Split1 offers an example where reversal and overlap of the track of sequentially recorded centroid positions introduces a model ambiguity, permitting some alternative interpretations to have slightly higher goodness-of-fit than the predicted case.
