# Supplementary figures and images for: Möbius-strip-like columnar functional connections are revealed in somato-sensory receptive field centroids
Source: Front Neuroanat. 2014 Oct 31;8:119. doi: 10.3389/fnana.2014.00119 (PMC4215792; doi:10.3389/fnana.2014.00119)

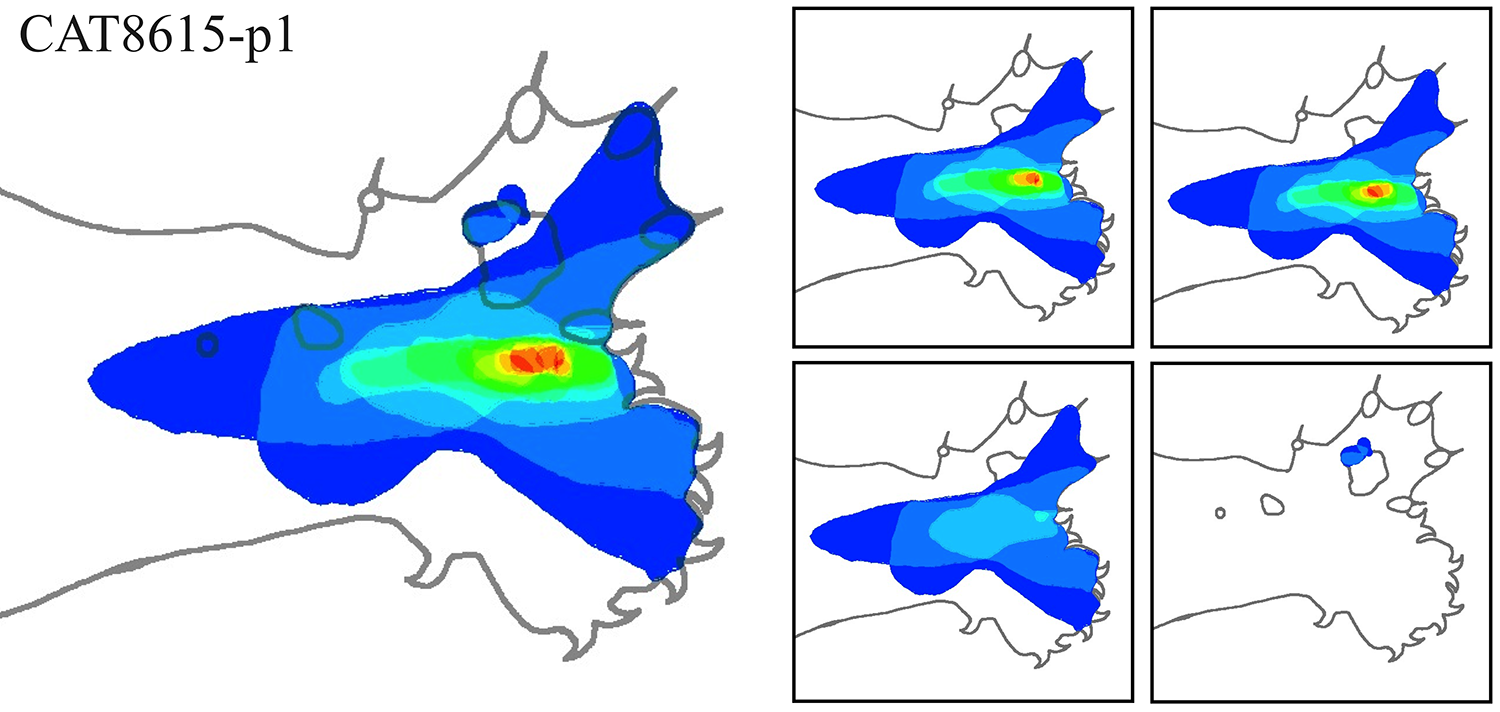

Supplement: Supplementary file 1 [file SupplementaryMaterial.ZIP › Supplementary/RF nests/seg_CAT8615-p1.png]

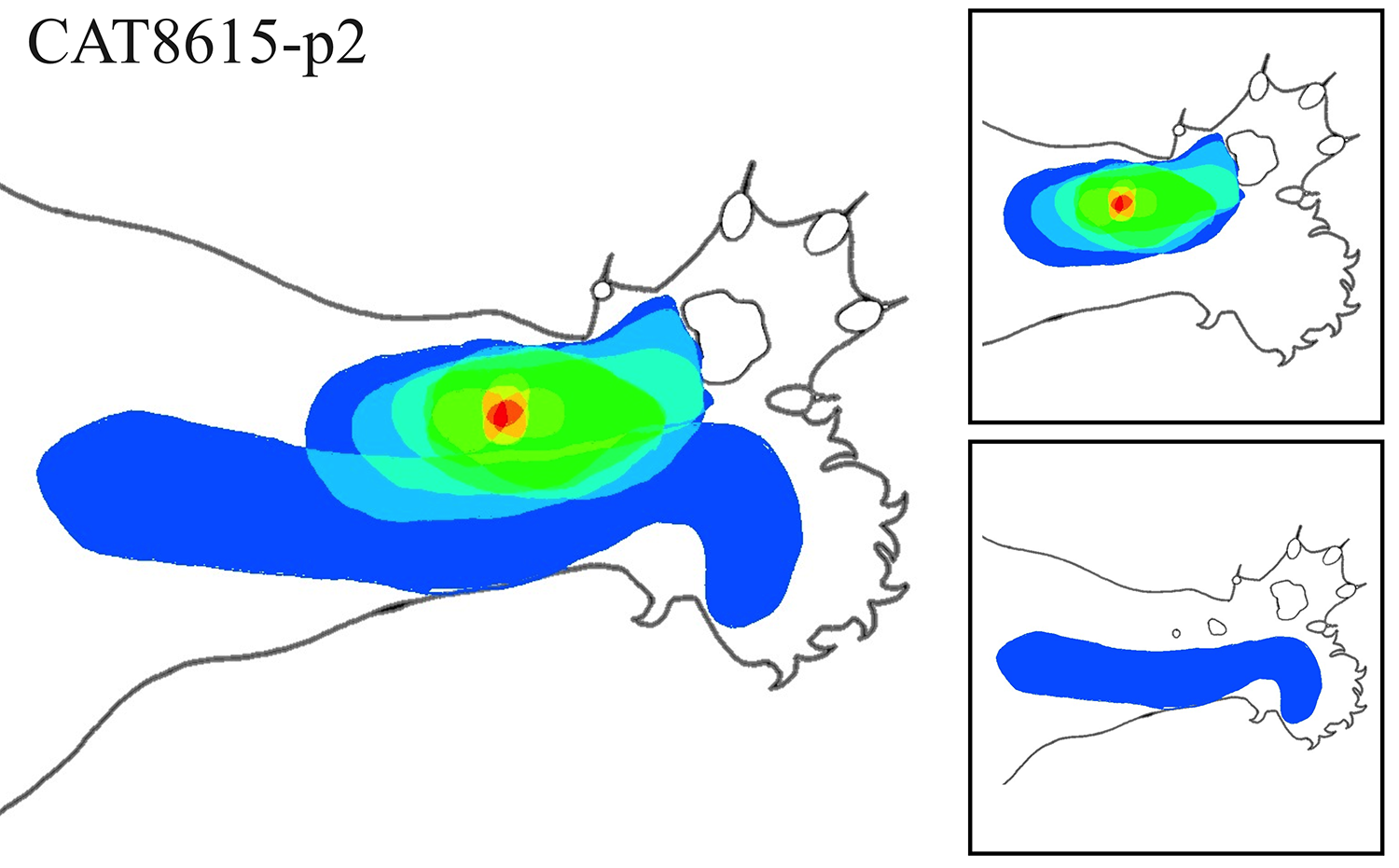

Supplement: Supplementary file 1 [file SupplementaryMaterial.ZIP › Supplementary/RF nests/seg_CAT8615-p2.png]

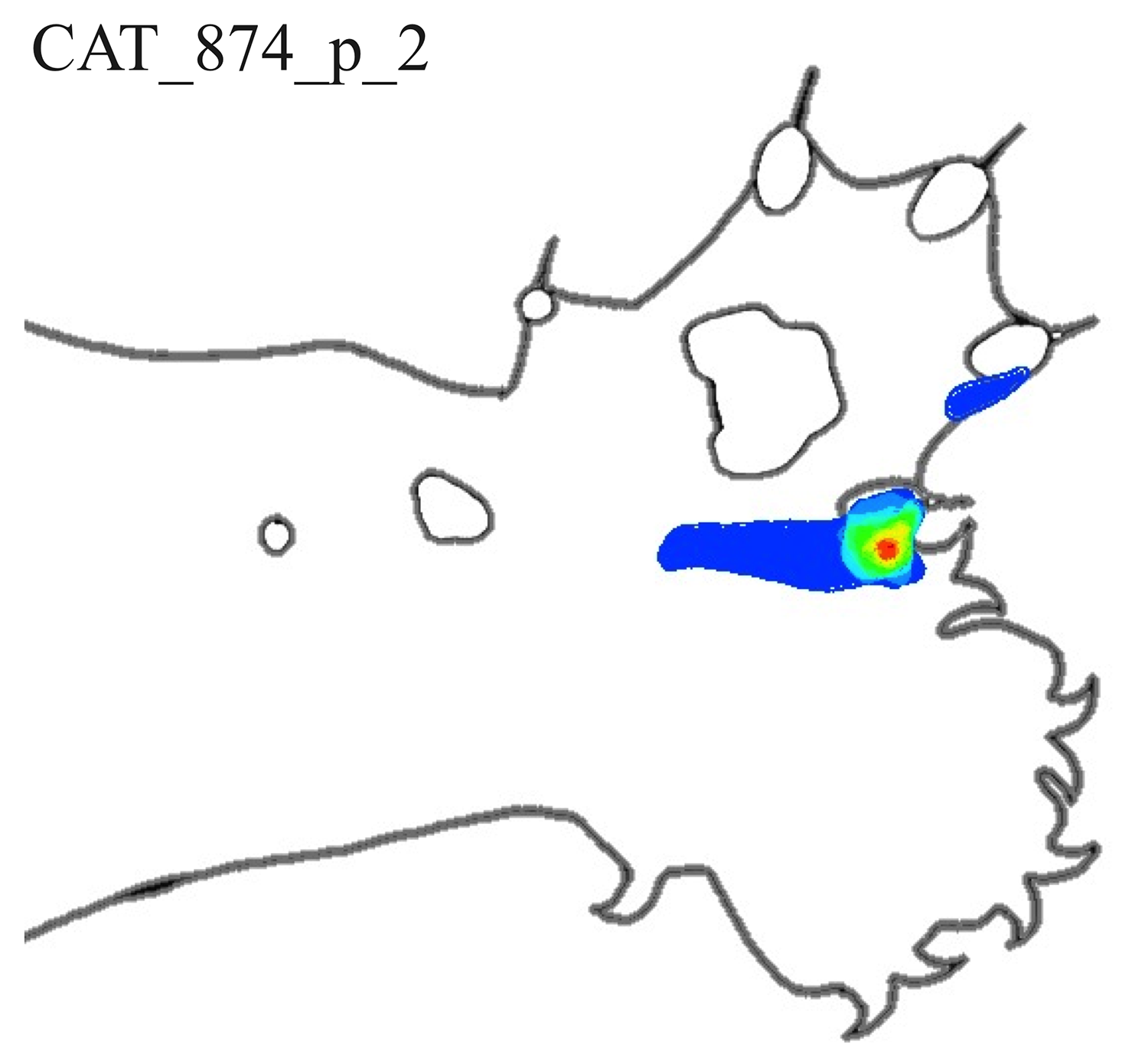

Supplement: Supplementary file 1 [file SupplementaryMaterial.ZIP › Supplementary/RF nests/seg_CAT_874_p_2.png]

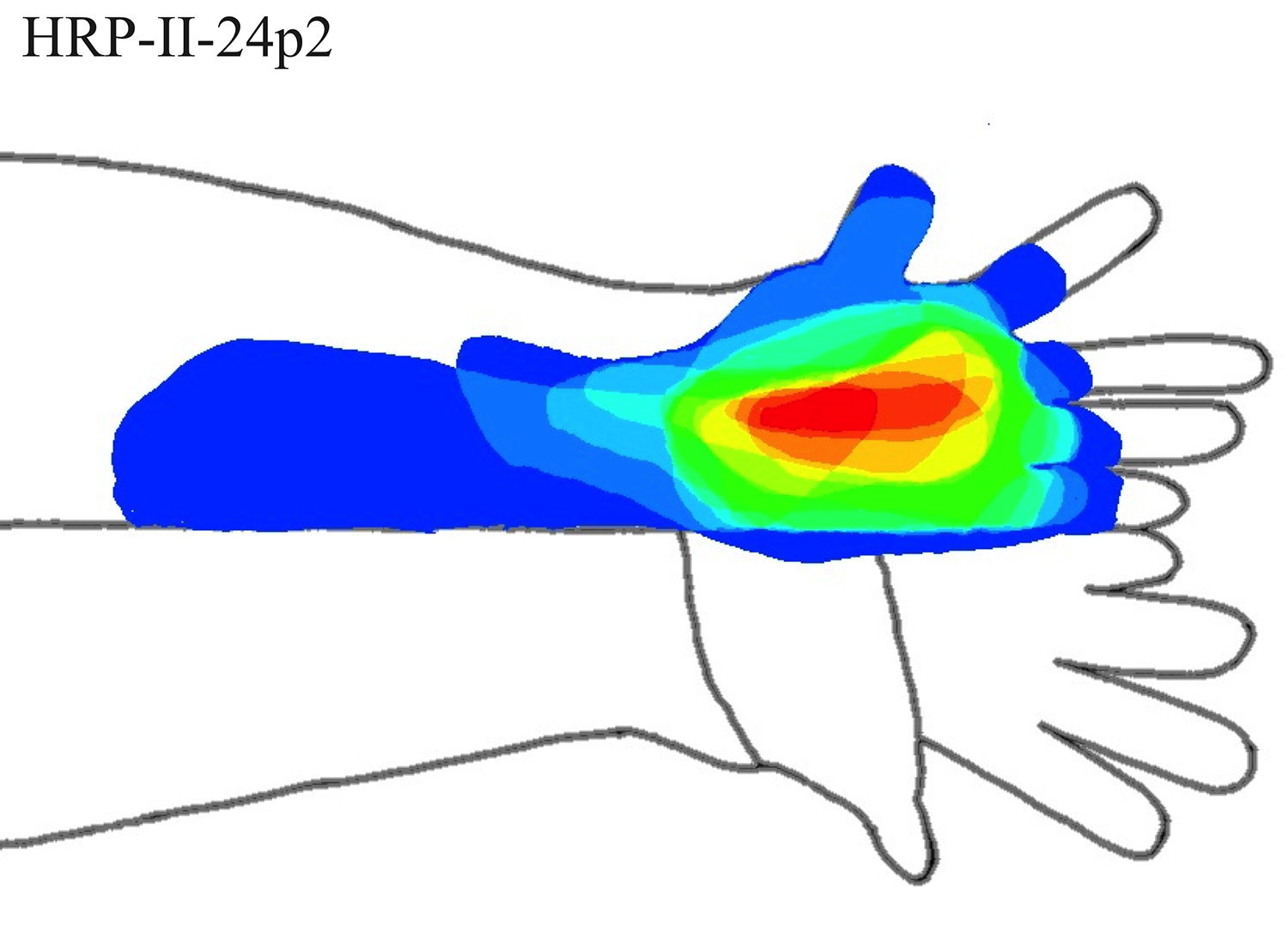

Supplement: Supplementary file 1 [file SupplementaryMaterial.ZIP › Supplementary/RF nests/seg_HRP-II-24p2.png]

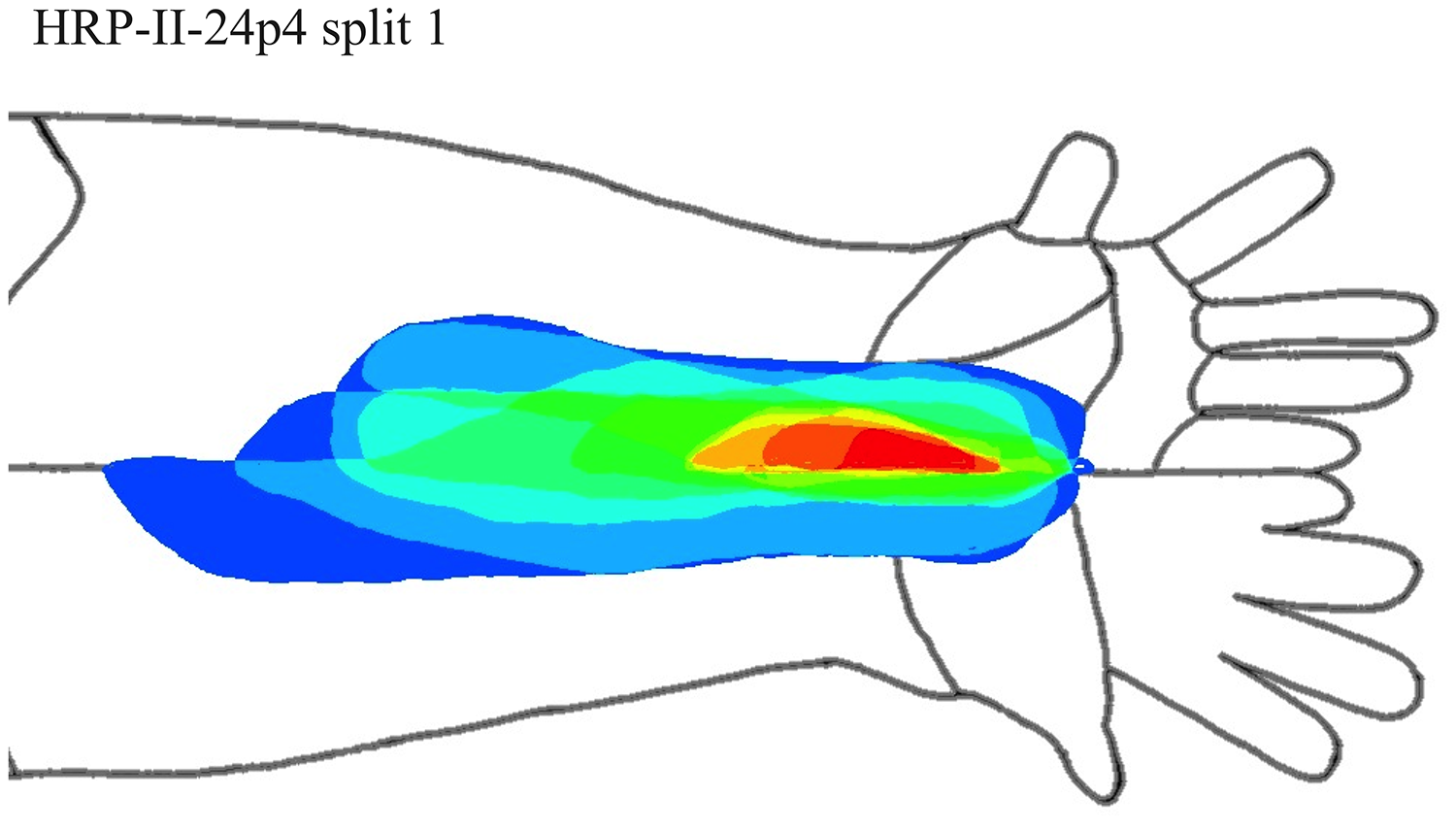

Supplement: Supplementary file 1 [file SupplementaryMaterial.ZIP › Supplementary/RF nests/seg_HRP-II-24p4_split1.png]

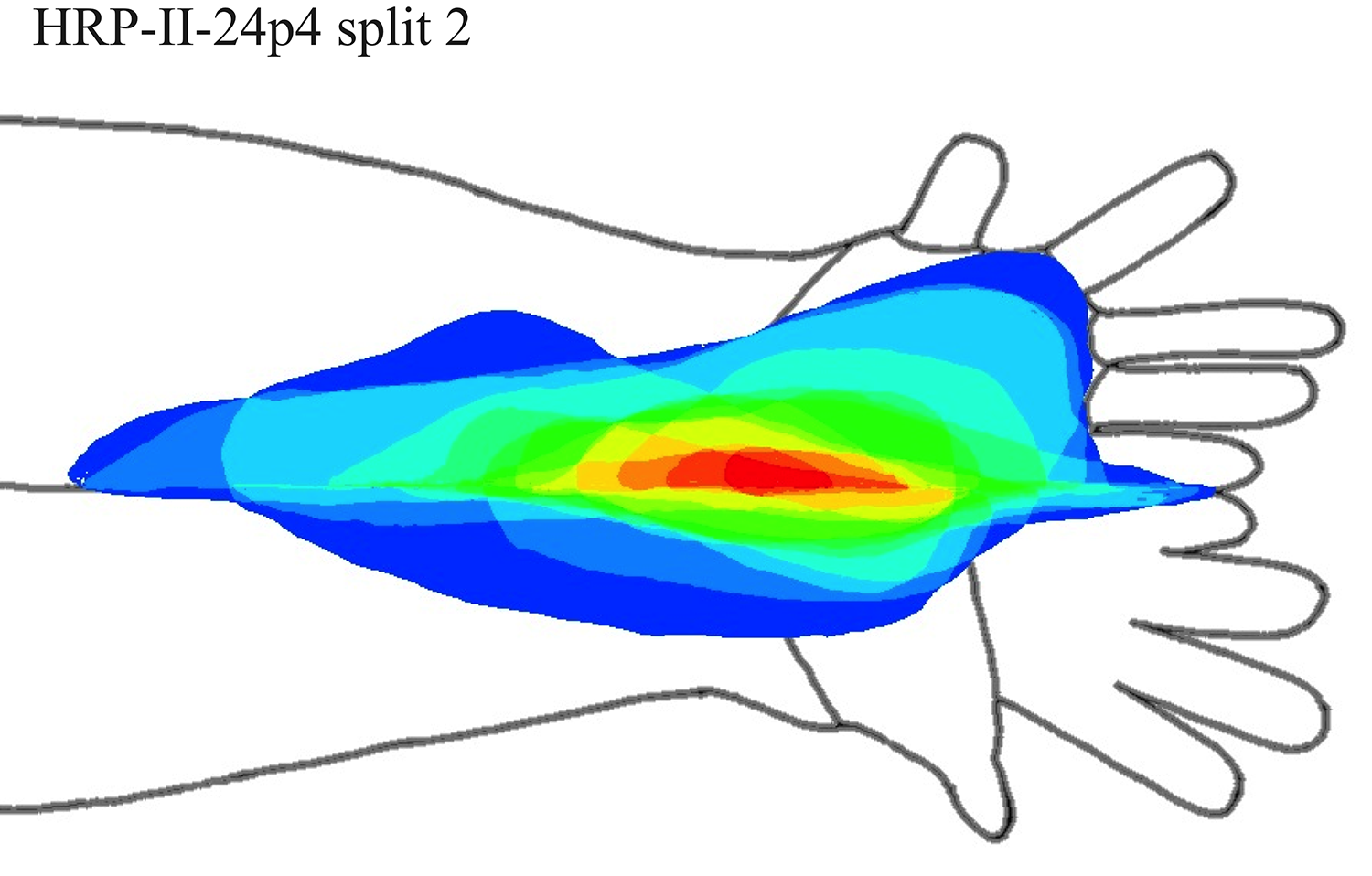

Supplement: Supplementary file 1 [file SupplementaryMaterial.ZIP › Supplementary/RF nests/seg_HRP-II-24p4_split2.png]

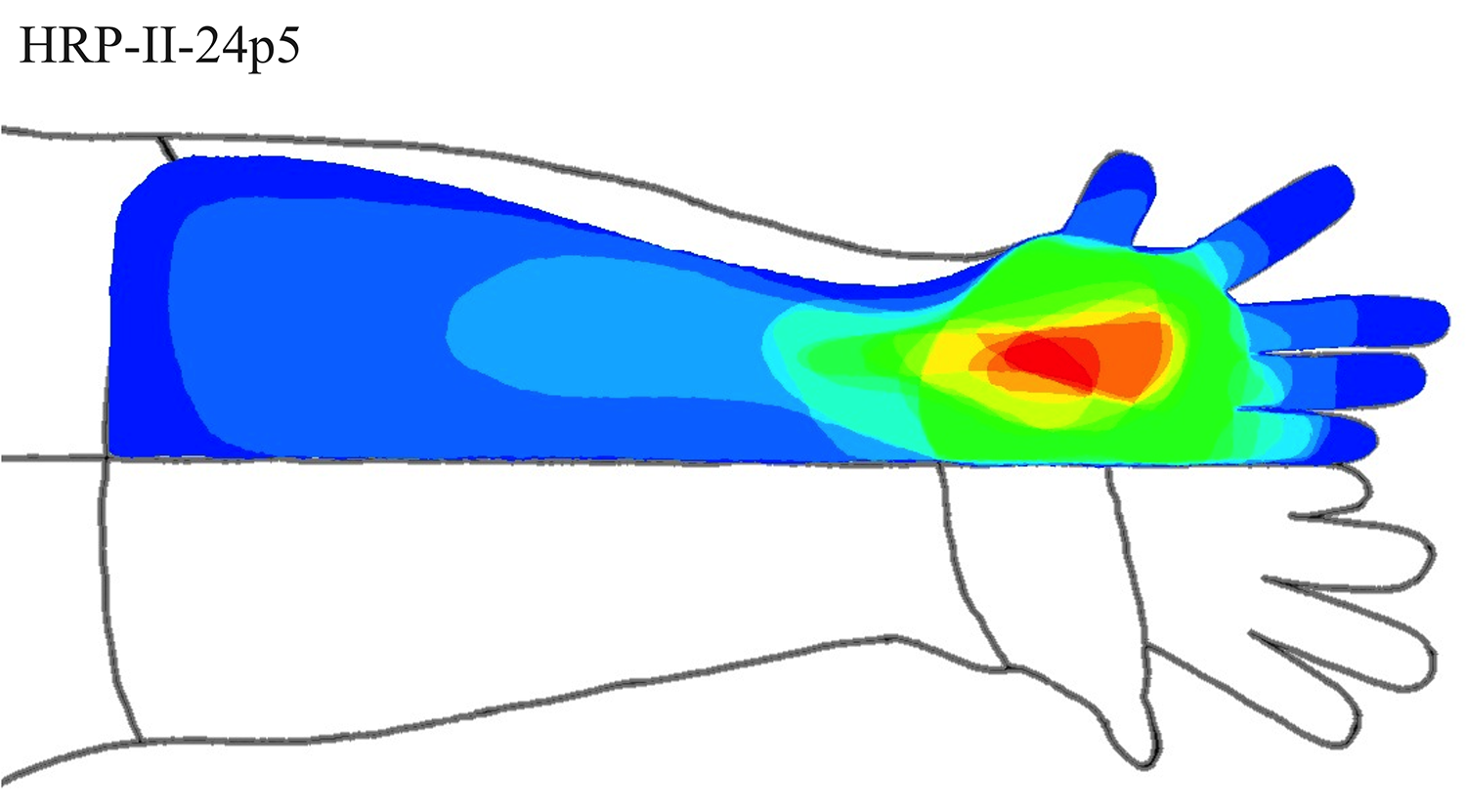

Supplement: Supplementary file 1 [file SupplementaryMaterial.ZIP › Supplementary/RF nests/seg_HRP-II-24p5.png]

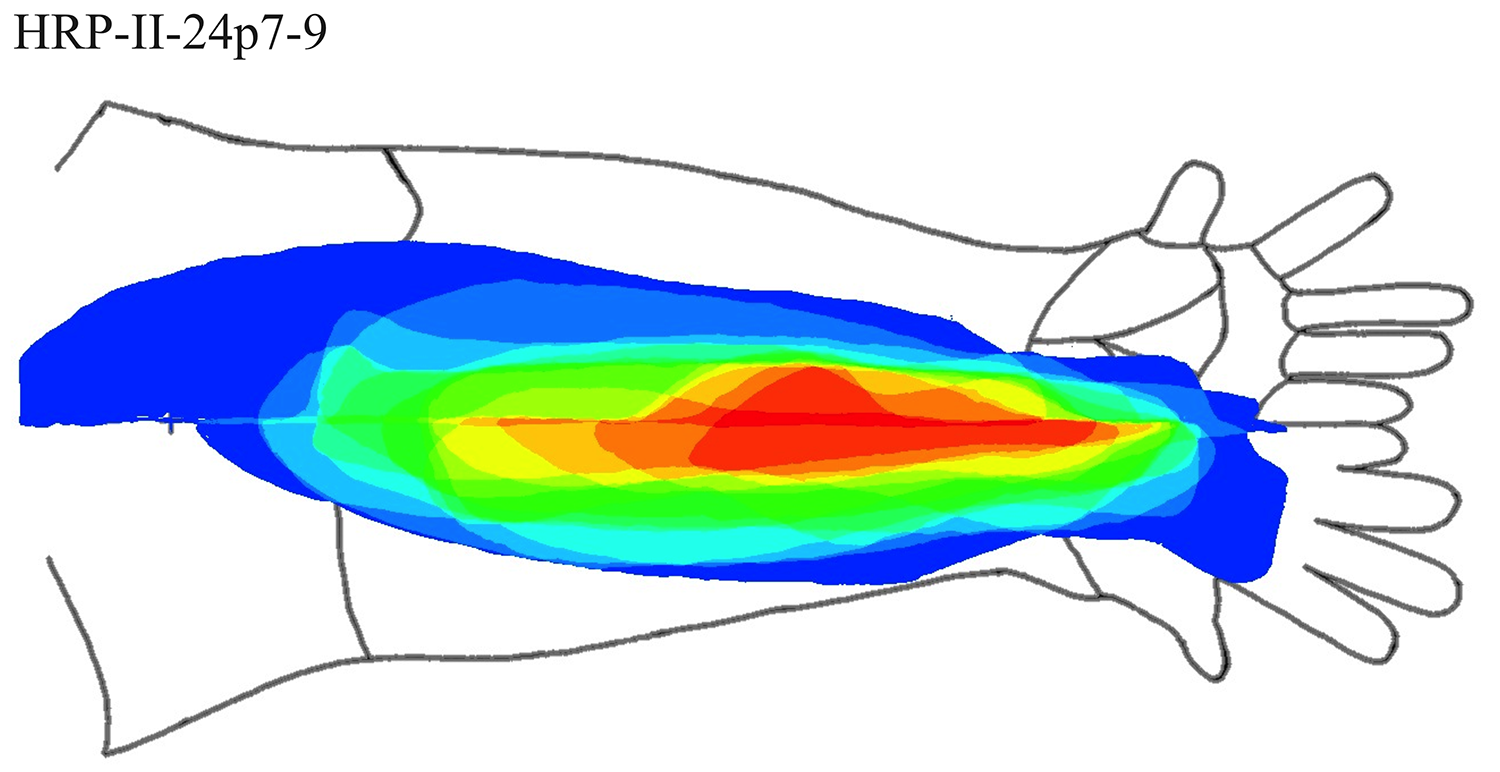

Supplement: Supplementary file 1 [file SupplementaryMaterial.ZIP › Supplementary/RF nests/seg_HRP-II-24p7-9.png]

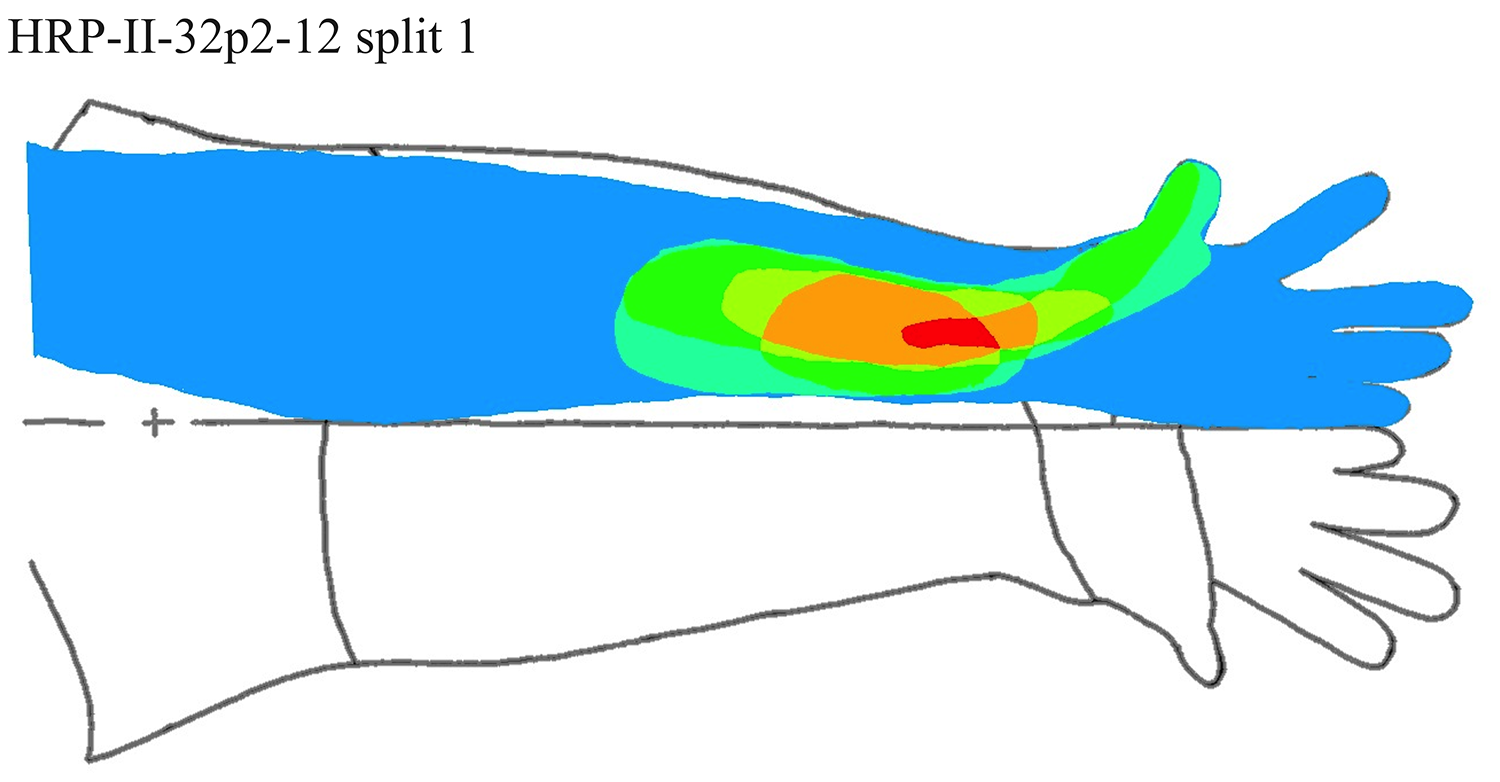

Supplement: Supplementary file 1 [file SupplementaryMaterial.ZIP › Supplementary/RF nests/seg_HRP-II-32p2-12_split1.png]

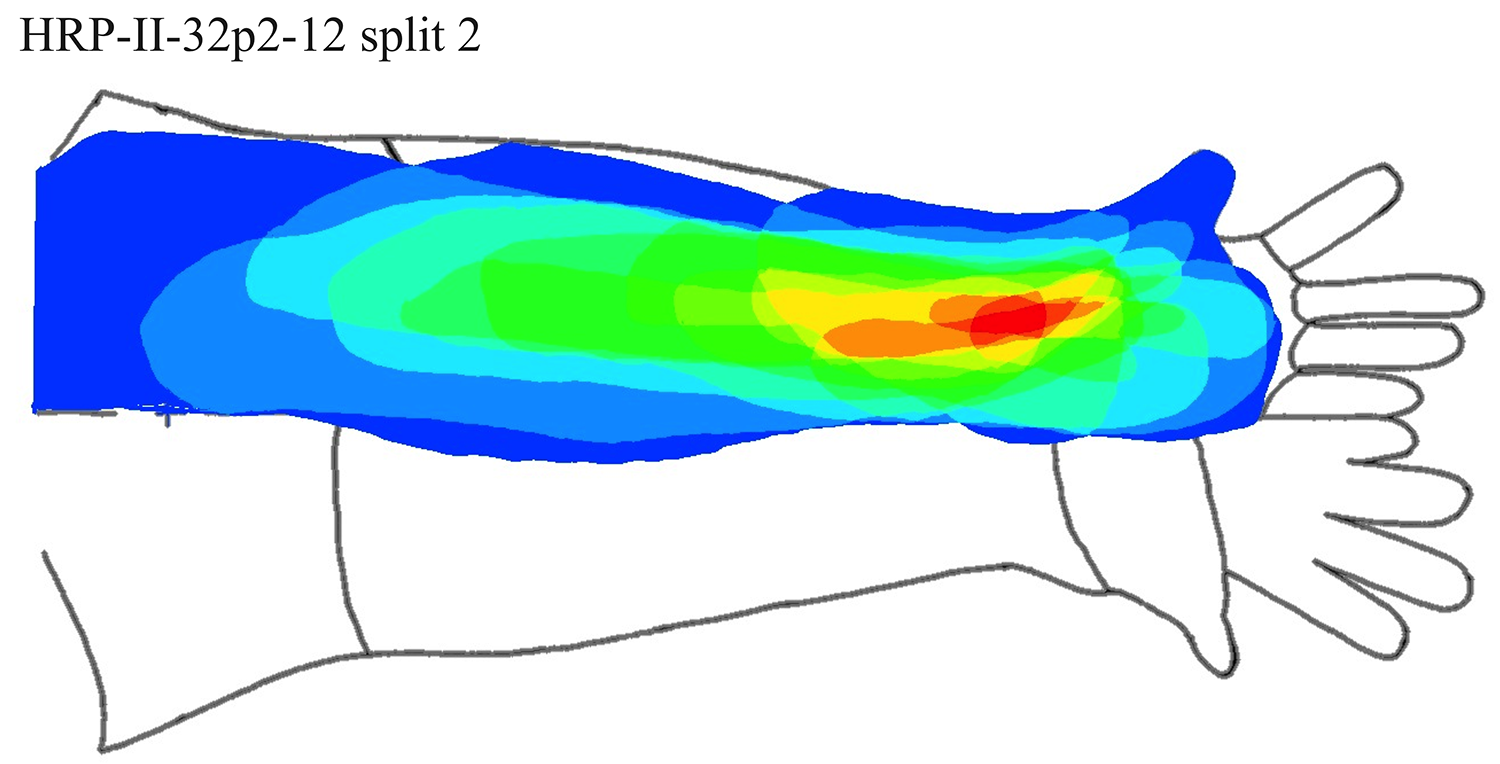

Supplement: Supplementary file 1 [file SupplementaryMaterial.ZIP › Supplementary/RF nests/seg_HRP-II-32p2-12_split2.png]

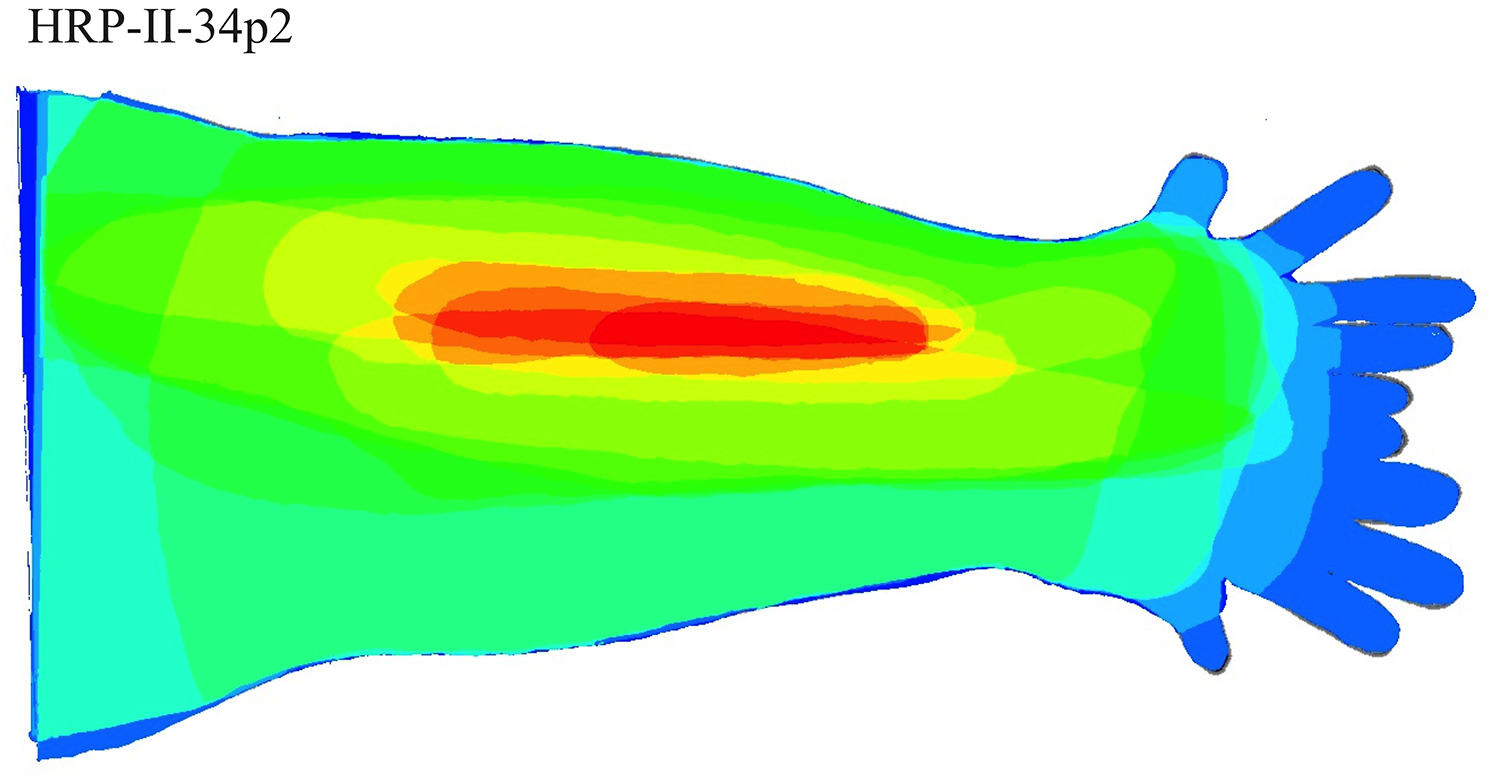

Supplement: Supplementary file 1 [file SupplementaryMaterial.ZIP › Supplementary/RF nests/seg_HRP-II-34p2.png]

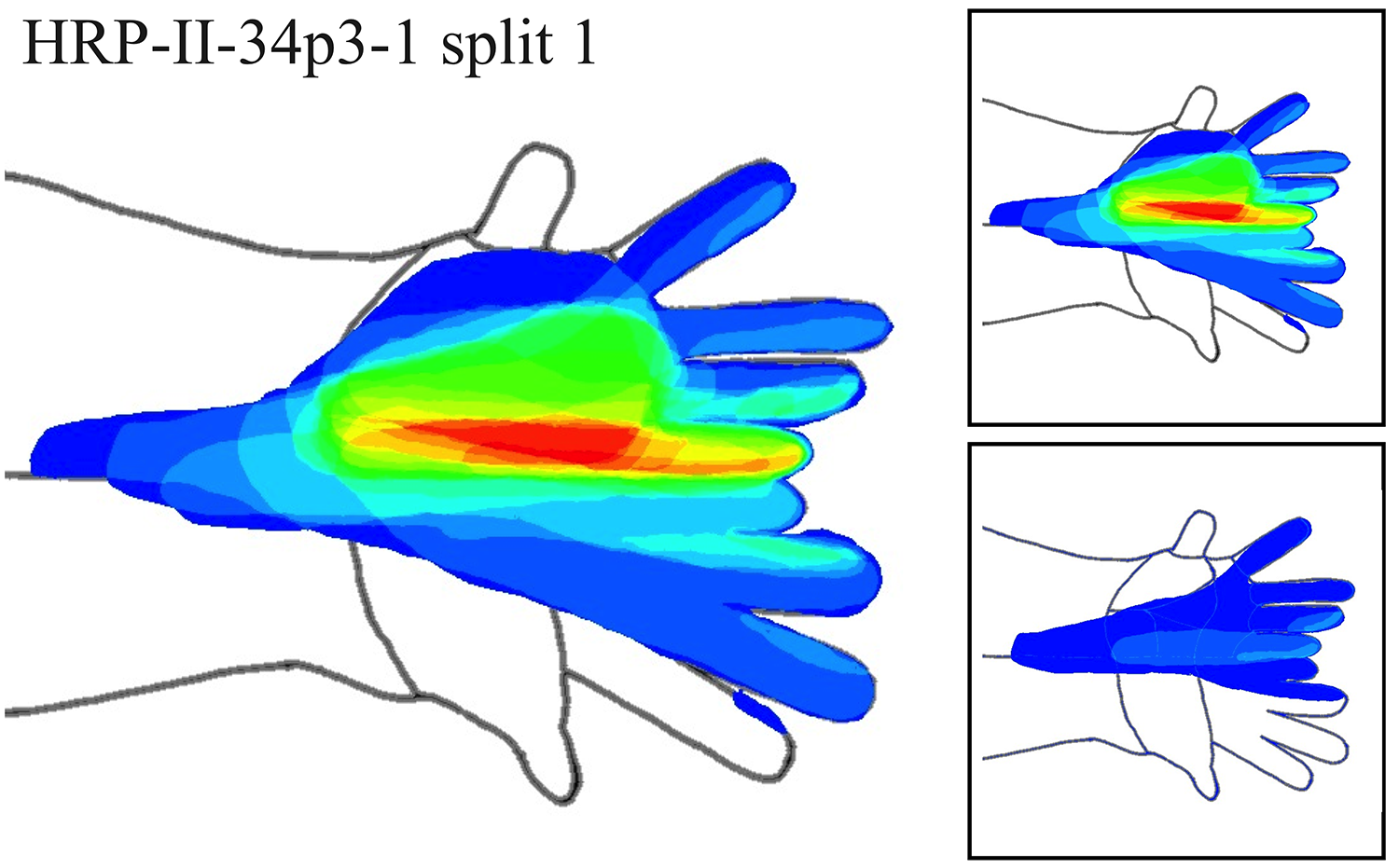

Supplement: Supplementary file 1 [file SupplementaryMaterial.ZIP › Supplementary/RF nests/seg_HRP-II-34p3-1_split1.png]

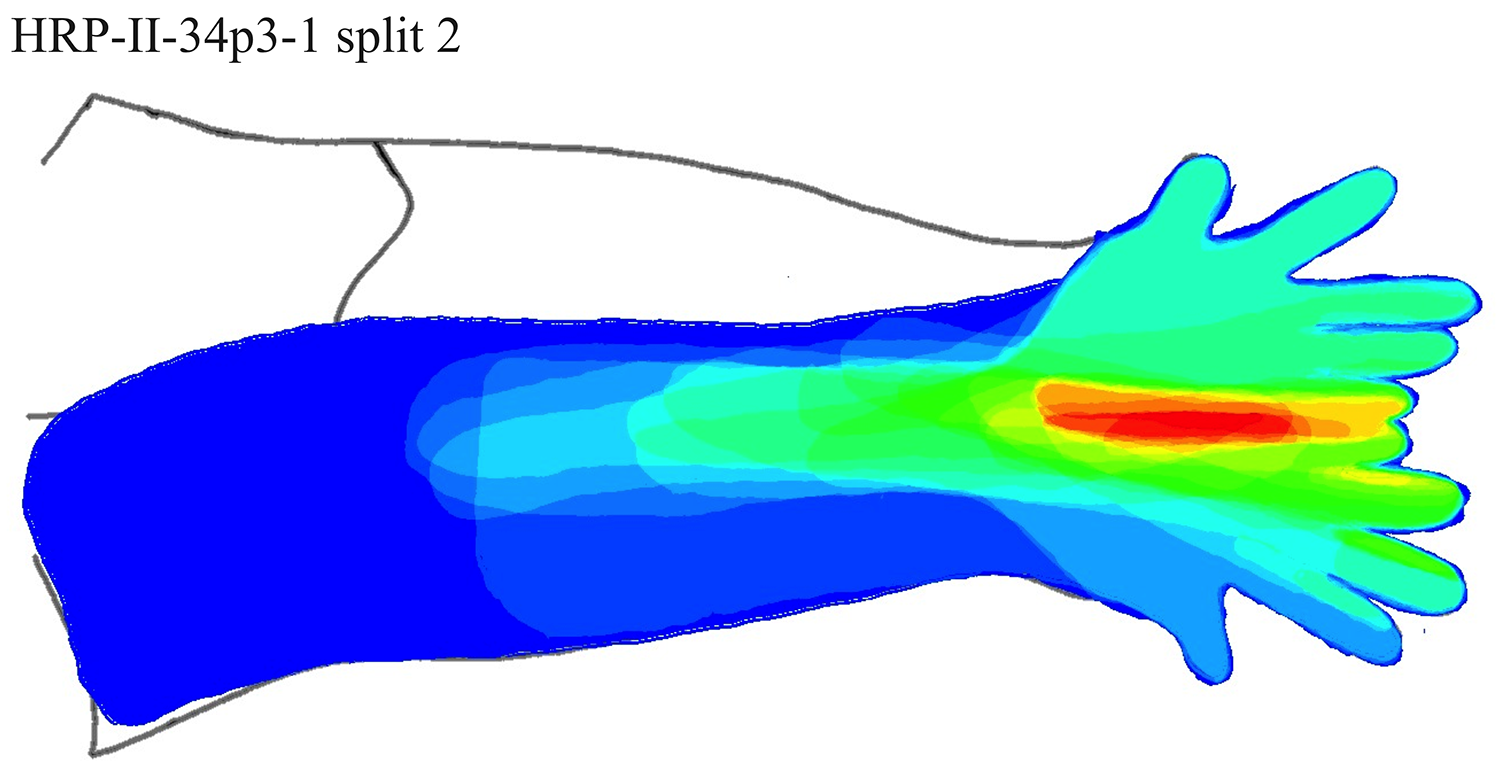

Supplement: Supplementary file 1 [file SupplementaryMaterial.ZIP › Supplementary/RF nests/seg_HRP-II-34p3-1_split2.png]

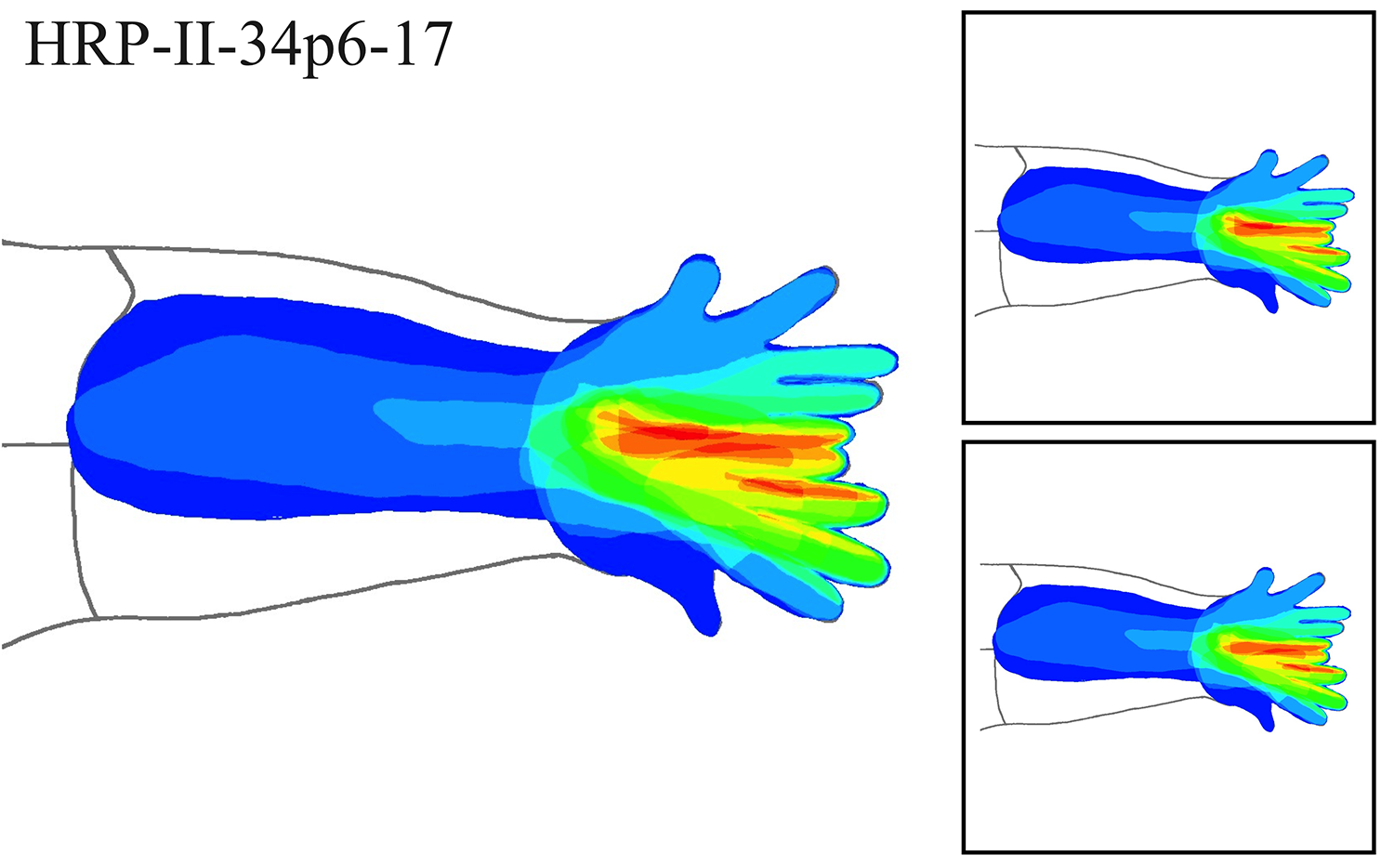

Supplement: Supplementary file 1 [file SupplementaryMaterial.ZIP › Supplementary/RF nests/seg_HRP-II-34p6-17.png]

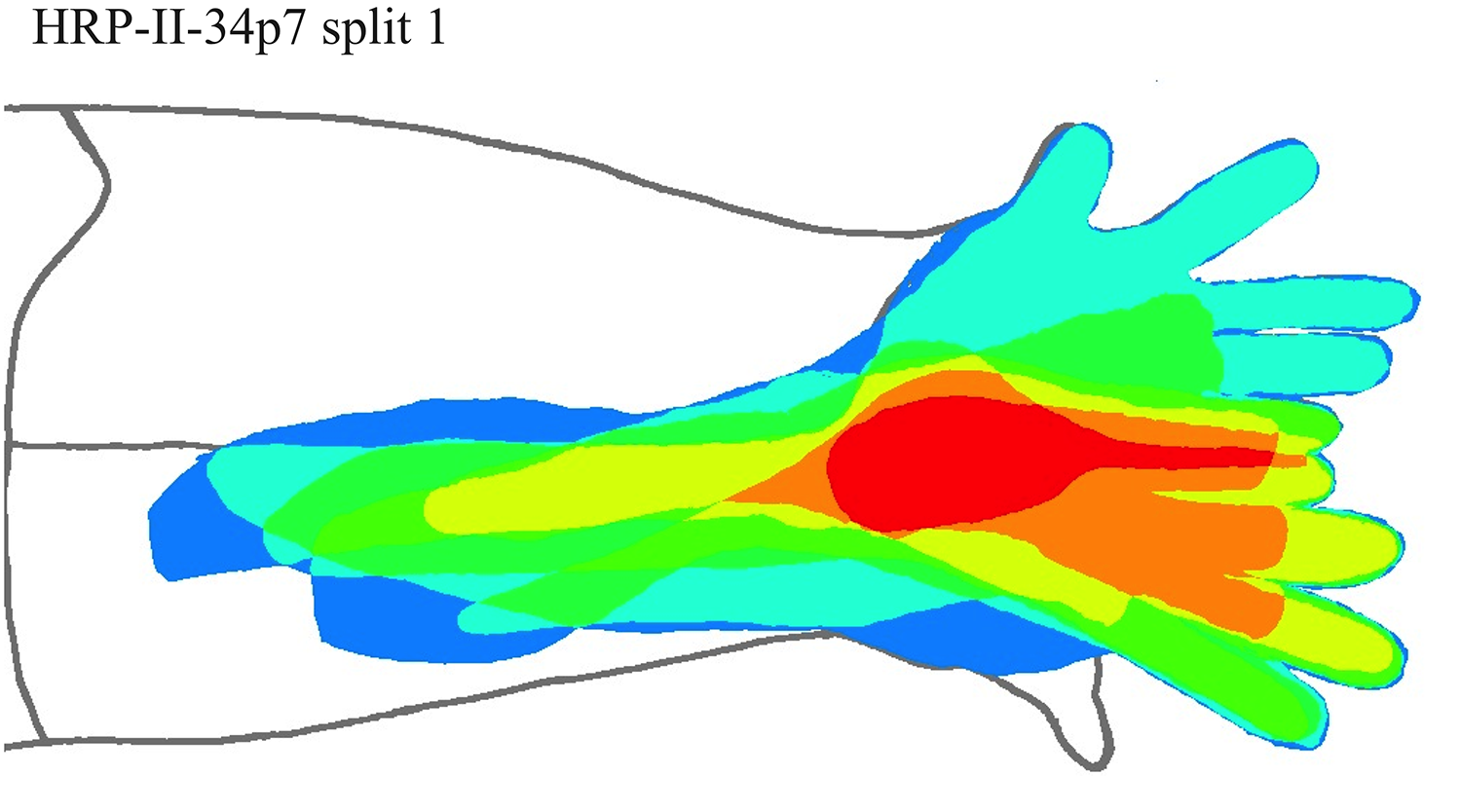

Supplement: Supplementary file 1 [file SupplementaryMaterial.ZIP › Supplementary/RF nests/seg_HRP-II-34p7_split1.png]

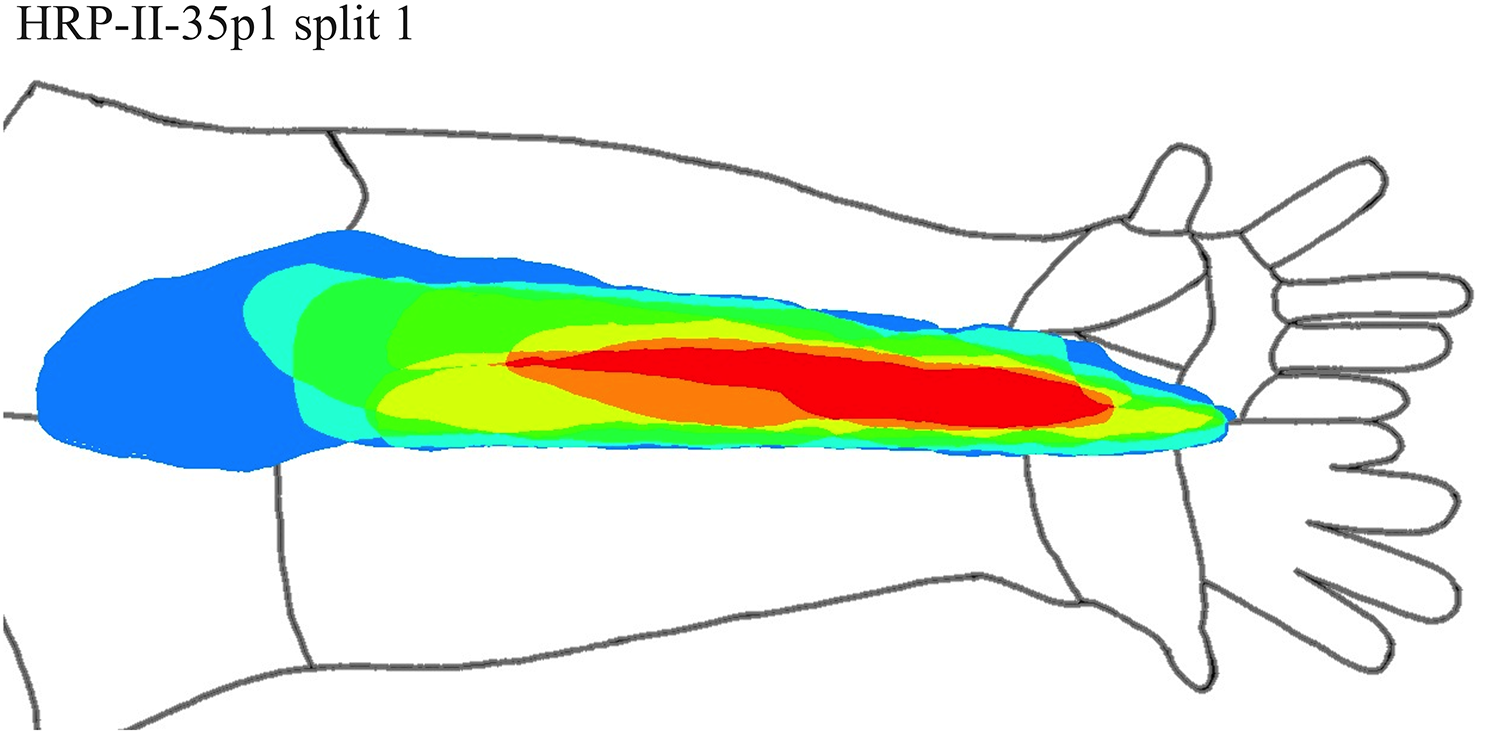

Supplement: Supplementary file 1 [file SupplementaryMaterial.ZIP › Supplementary/RF nests/seg_HRP-II-35p1_split1.png]

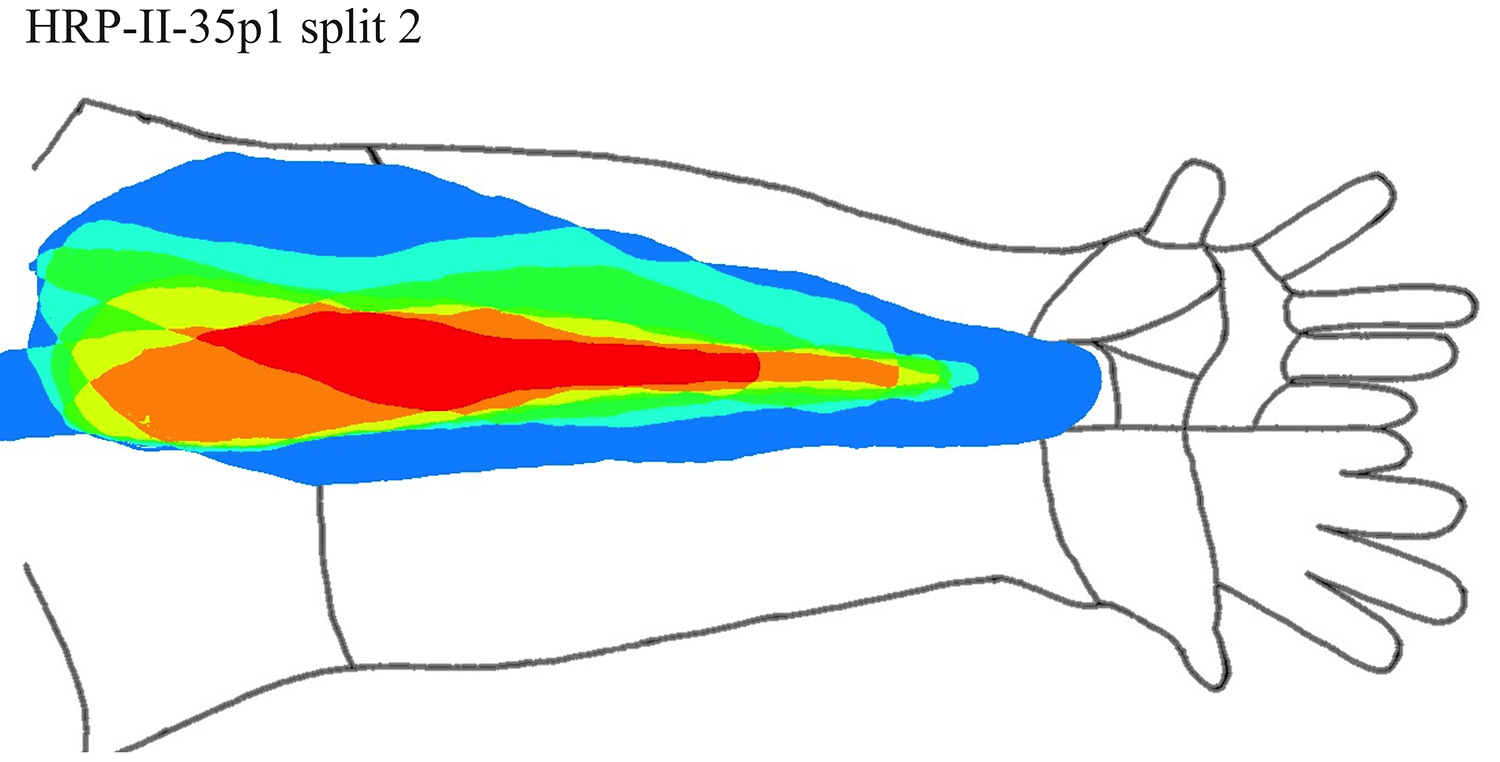

Supplement: Supplementary file 1 [file SupplementaryMaterial.ZIP › Supplementary/RF nests/seg_HRP-II-35p1_split2.png]

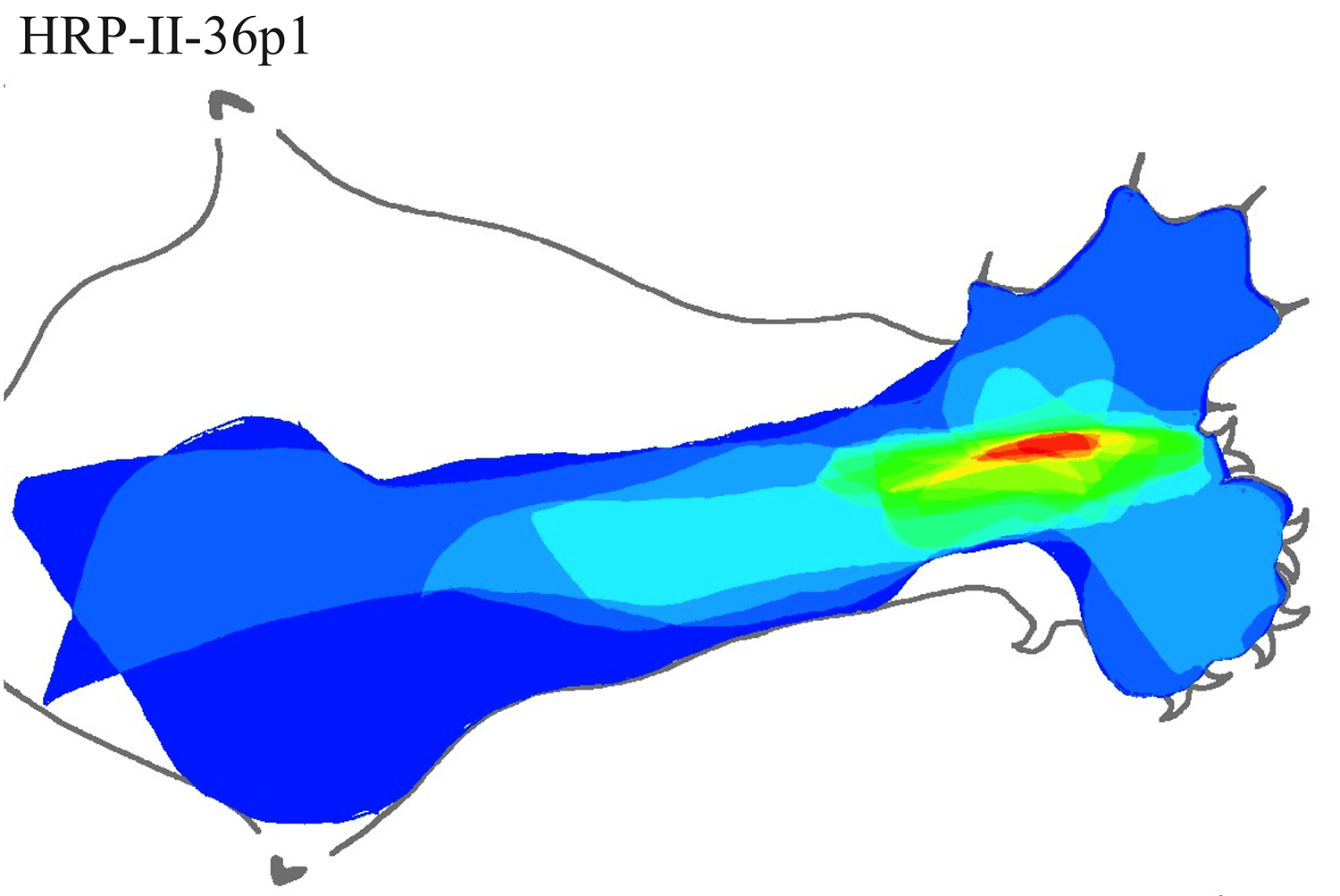

Supplement: Supplementary file 1 [file SupplementaryMaterial.ZIP › Supplementary/RF nests/seg_HRP-II-36p1.png]

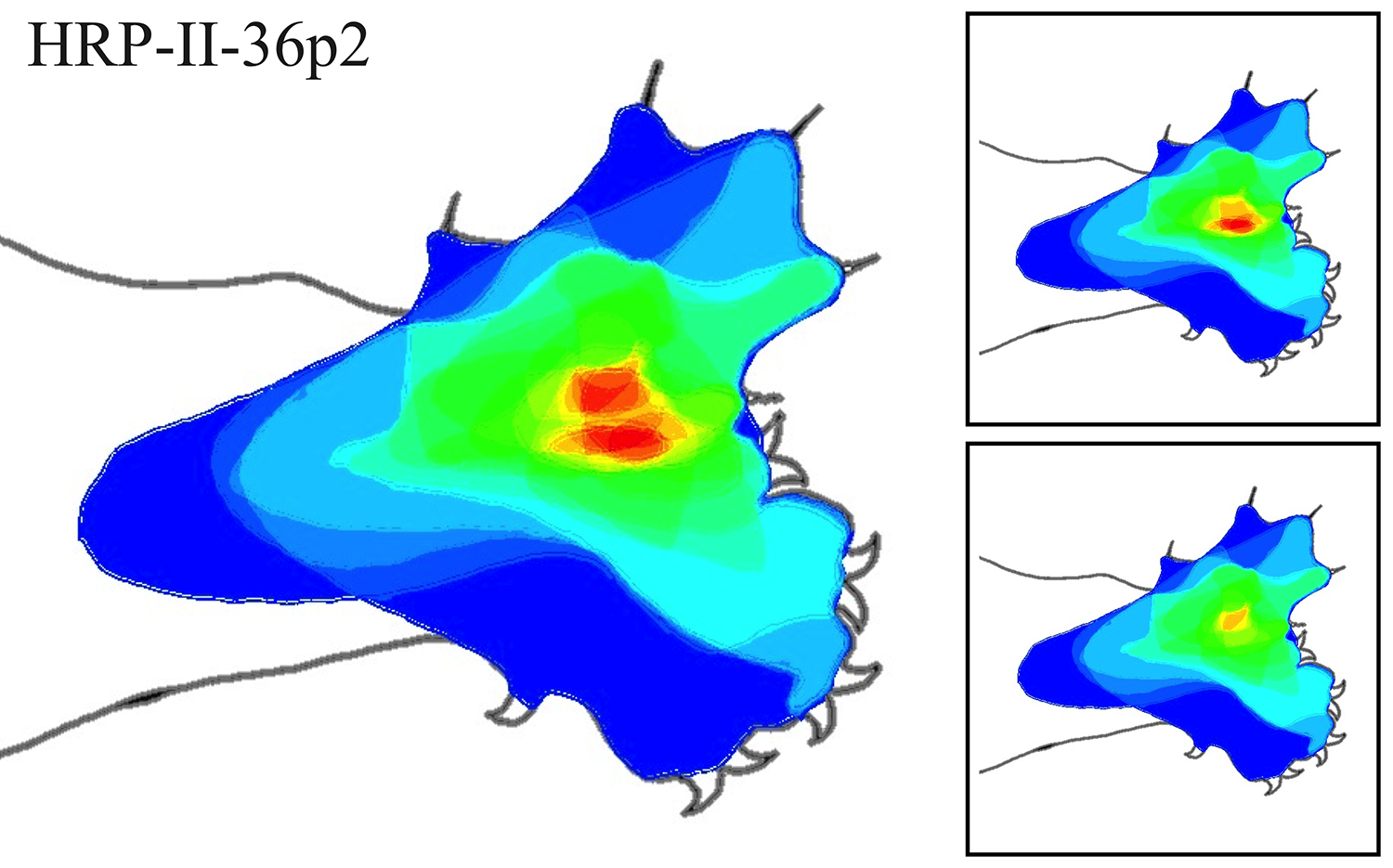

Supplement: Supplementary file 1 [file SupplementaryMaterial.ZIP › Supplementary/RF nests/seg_HRP-II-36p2.png]

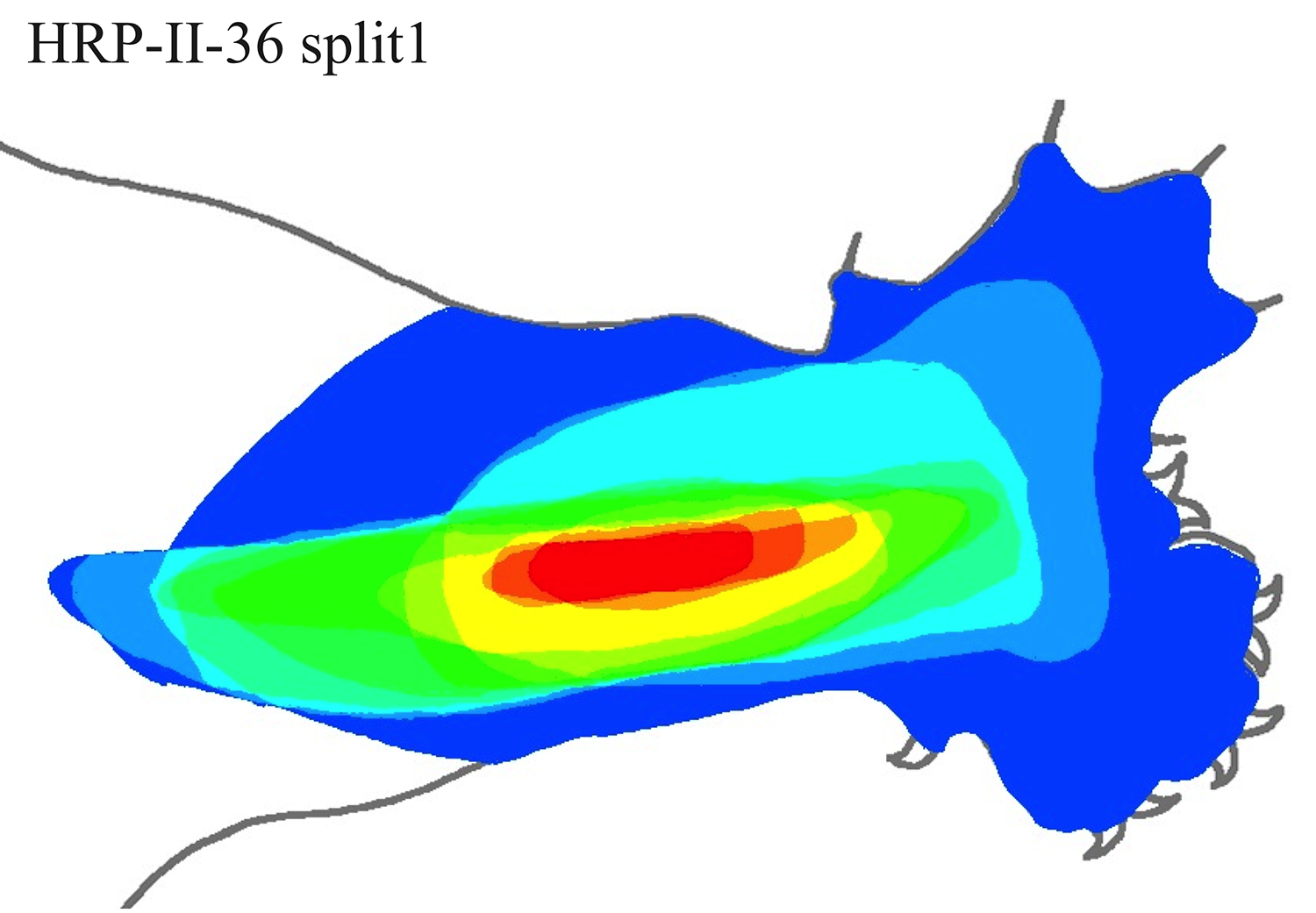

Supplement: Supplementary file 1 [file SupplementaryMaterial.ZIP › Supplementary/RF nests/seg_HRP-II-36_split1.png]

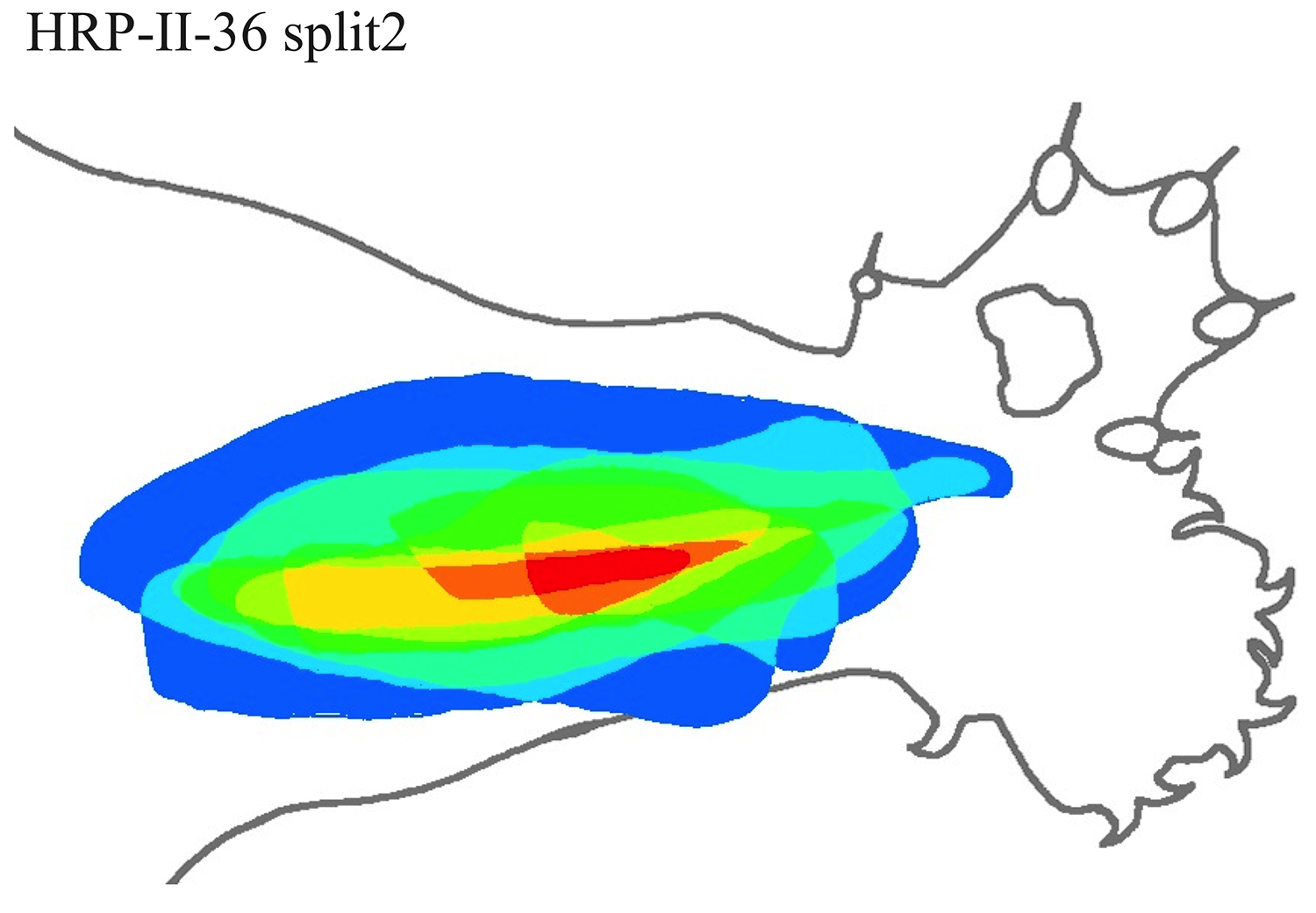

Supplement: Supplementary file 1 [file SupplementaryMaterial.ZIP › Supplementary/RF nests/seg_HRP-II-36_split2.png]
